# A systematic review to identify biomarkers of intake for fermented food products

**DOI:** 10.1186/s12263-021-00686-4

**Published:** 2021-04-21

**Authors:** Katherine J. Li, Elske M. Brouwer-Brolsma, Kathryn J. Burton-Pimentel, Guy Vergères, Edith J. M. Feskens

**Affiliations:** 1 Division of Human Nutrition and Health, Department of Agrotechnology and Food Science, Wageningen University & Research, Wageningen, Netherlands; 2 Food Microbial Systems Research Division, Federal Department of Economic Affairs, Education and Research (EAER), Federal Office for Agriculture (FOAG), Agroscope, Bern, Switzerland

**Keywords:** Fermented foods, Dietary biomarkers, Metabolites, Dietary assessment, Food intake biomarkers

## Abstract

**Background:**

Fermented foods are ubiquitous in human diets and often lauded for their sensory, nutritious, and health-promoting qualities. However, precise associations between the intake of fermented foods and health have not been well-established. This is in part due to the limitations of current dietary assessment tools that rely on subjective reporting, making them prone to memory-related errors and reporting bias. The identification of food intake biomarkers (FIBs) bypasses this challenge by providing an objective measure of intake. Despite numerous studies reporting on FIBs for various types of fermented foods and drinks, unique biomarkers associated with the fermentation process (“fermentation-dependent” biomarkers) have not been well documented. We therefore conducted a comprehensive, systematic review of the literature to identify biomarkers of fermented foods commonly consumed in diets across the world.

**Results:**

After title, abstract, and full-text screening, extraction of data from 301 articles resulted in an extensive list of compounds that were detected in human biofluids following the consumption of various fermented foods, with the majority of articles focusing on coffee (69), wine (69 articles), cocoa (62), beer (34), and bread (29). The identified compounds from all included papers were consolidated and sorted into FIBs proposed for a specific food, for a food group, or for the fermentation process. Alongside food-specific markers (e.g., trigonelline for coffee), and food-group markers (e.g., pentadecanoic acid for dairy intake), several fermentation-dependent markers were revealed. These comprised compounds related to the fermentation process of a particular food, such as mannitol (wine), 2-ethylmalate (beer), methionine (sourdough bread, cheese), theabrownins (tea), and gallic acid (tea, wine), while others were indicative of more general fermentation processes (e.g., ethanol from alcoholic fermentation, 3-phenyllactic acid from lactic fermentation).

**Conclusions:**

Fermented foods comprise a heterogeneous group of foods. While many of the candidate FIBs identified were found to be non-specific, greater specificity may be observed when considering a combination of compounds identified for individual fermented foods, food groups, and from fermentation processes. Future studies that focus on how fermentation impacts the composition and nutritional quality of food substrates could help to identify novel biomarkers of fermented food intake.

**Supplementary Information:**

The online version contains supplementary material available at 10.1186/s12263-021-00686-4.

## Background

Fermentation as a food processing technology has been used for millennia to enhance the flavor, texture, and nutritive value of foods, as well as to improve their transportability, storage time, and/or safety [1, 2]. Fermentation techniques continue to be refined and applied to a wide range of foods, including milk, grains, legumes, fruits, vegetables, fish, and meat products. Common types of fermented foods vary by region; for example, fermented dairy products (e.g., cheese, yoghurt, buttermilk) are produced and consumed abundantly in Europe, fermented pulses and cereals (e.g., dosai, idli, injera) are mostly indigenous to South Asia and Africa, and fermented soy products (e.g., natto, miso, soy sauce, doubanjiang) are particularly common in East Asia [3–5]. Other products are less regionally or culturally dependent, such as fermented fish products that are consumed in Korea (sikhae) and Japan (narezushi), as well as Sweden (surströmming) and Norway (rakfisk) [4, 6]. The endless combination of foods (or “substrates”), microorganisms, and fermentation techniques results in global fermented products with vastly different sensory and nutritional profiles. Currently, over 5000 types of fermented foods and beverages exist worldwide [7], and continued growth of the fermented food market is anticipated and fuelled by health food trends and rejuvenated artisanal practices.

Fermented foods are produced by the controlled growth and enzymatic activities of microorganisms, through four main fermentation processes (lactic, acetic, alcoholic, and alkaline) [4, 8]. Lactic fermentations are carried out by lactic acid bacteria (LAB) (predominantly *Lactobacillus*, *Streptococcus*, *Pediococcus*, and *Leuconostoc*) for the production of fermented dairy, meat, and vegetable products, whereas acetic acid bacteria (e.g., *Gluconacetobacter*) are responsible for the fermentation of cocoa, vinegar, and kombucha [9, 10]. Alcoholic fermentations are driven by yeasts (e.g., *Saccharomyces cerevisiae)* for the production of beer, wine, and breads, while alkaline fermentations make use of fungi (e.g., *Penicillium* spp. and *Aspergillus* spp.) during the production and maturation of cheese, fermented meats, and fermented soy products [11, 12]. Irrespective of the type of fermentation, microbial enzymes interact with the food matrix to produce novel metabolites, which can affect the sensory and functional profile of foods [13–20]. and have also been suggested to possess bioactive qualities that can help prevent chronic diseases such as diabetes and cardiovascular disease [17].

Fundamental research suggests that the protective effects of fermented foods may be explained by fermentation-induced increases in the bioavailability of certain macro- and micronutrients (e.g., protein, vitamins) [21], fermentation-induced decreases in anti-nutritional compounds [22], or driven by novel bioactive compounds of microbial metabolism [7]. While several human observational studies have indicated a possible beneficial association between fermented food consumption and cardiometabolic health, specifically in terms of weight maintenance, diabetes/glucose homeostasis, and overall cardiovascular disease risk [23–25], the evidence is still inconclusive, in part due to the limitations of tools used to quantify fermented food intake.

Currently, self-report food frequency questionnaires (FFQs), 24-h recalls, and food diaries (weighed or unweighed) are the most commonly used dietary assessment tools to quantify food intake. The FFQ is usually the method of choice in observational cohort studies. In contrast to diaries and recalls, FFQs are relatively easy to administer and process, but their accuracy relies on the memory and devotion of respondents. Consequently, random and systematic errors, including memory-related bias, incorrect estimates of portion sizes, and/or bias towards socially desirable answers, are inevitable [26, 27]. There is also no FFQ that has been specifically designed to estimate fermented food intake, and food lists in existing FFQs may not comprehensively cover the intakes of this diverse food group, or distinguish the nuances within specific foods that affect their fermentation status (e.g., fermented pickles vs. acidified pickles). Moreover, none of the self-report dietary assessment methods takes into account differences in food metabolism between individuals, which can have a significant bearing on the immediate effects of diet and subsequent health consequences. The importance of accurately assessing food intake across diverse populations has propelled food intake biomarkers (FIBs), as promising “objective” measures of intake and metabolism, to the forefront of dietary assessment research [26, 28].

While the identification of a single specific biomarker is ideal, this is not always possible due to the overlapping characteristics shared by many foods. A number of combination biomarkers have thus been proposed, for example in the case of red wine (tartaric acid reflecting the grape raw material, plus ethyl glucuronide reflecting alcoholic fermentation and phase II metabolism) [29]. A similar approach is expected to be suitable for identifying FIBs for other fermented food products, such that a group of compounds, although not unique to the food themselves, might be useful in combination to stratify between high and low consumers of different fermented foods in intervention studies and epidemiological cohorts.

The Food Biomarker Alliance (FoodBAll) [28], a project funded by the Joint Programming Initiative a Healthy Diet for a Healthy Life, has set guidelines for identifying and validating FIBs [30] (http://foodmetabolome.org) [31]. This effort has resulted in a systematic documentation of FIBs for major food groups, including fruit and vegetables, meats, fish, and other seafood, dairy products, cereals and whole grains, alcoholic and non-alcoholic beverages, vegetable oils, nuts, and spices and herbs [32–40]. The purpose of the current systematic review is to present a comprehensive overview of compounds reported in the literature that could, alone or in combination, represent FIBs for various fermented foods. We anticipated that identified compounds could be stratified into FIBs at the food level, food group level, and fermentation level, to discriminate a dietary pattern of fermented food consumption.

## Methods

### Primary database search

**Fig. 1:**
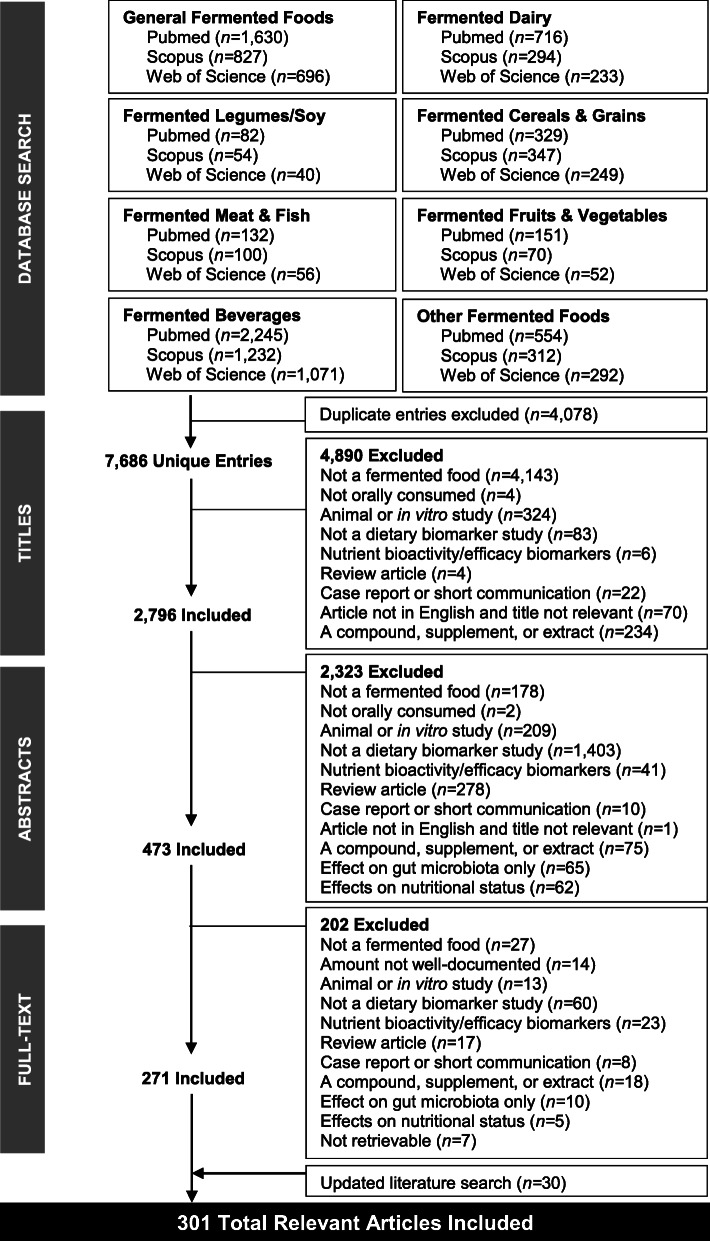
Schematic outline of the systematic literature search

The literature search strategy and search terms were developed in accordance with the guidelines previously proposed by the FoodBAll consortium [30], and all elements of the PRISMA (Preferred Reporting Items for Systematic reviews and Meta-Analyses) statement relevant for a literature search on biomarkers were reported [41]. Primary articles were identified from PubMed, Scopus, and ISI Web of Knowledge. In order to obtain a broad coverage of fermented food products consumed globally, eight food groups were defined for the search strategy, specifically: (i) general fermented foods, (ii) fermented dairy, (iii) fermented meats and fish, (iv) fermented fruits and vegetables, (v) fermented legumes (including soy), (vi) fermented cereals and grains, (vii) fermented beverages, and (viii) other fermented products (e.g., chocolate, condiments, and sauces). These food groups were loosely based on the food-based dietary guidelines in The Netherlands, Switzerland, and the USA [42–44], but were inclusive of fermented food items consumed worldwide. Individual fermented foods were further specified within each food group, as detailed in Additional File 1. Exclusion terms were individually applied to each search to limit the number of false-positive hits. Each of the eight food group terms was searched for in conjunction with a combination of search operators, as detailed in Additional File 2. The search fields applied were [Title/Abstract] for PudMed, [Title/Abstract/Keywords] for Scopus, and [Topic] for ISI Web of Knowledge. All searches were conducted in October 2018, and an updated literature search was performed in September 2020. No restrictions were applied on the publication date. Furthermore, the reference lists of relevant systematic reviews and meta-analyses [32–34] were scanned for relevant articles for inclusion. The full literature search process is outlined in Fig. 1.

### Inclusion and exclusion criteria

Search inclusion and exclusion criteria were defined a priori. Studies were included if the primary exposure was oral consumption of a fermented food, where “fermented food” was defined according to the definition given by Marco et al.: “foods or beverages made through controlled microbial growth and enzymatic conversions of major and minor food components” [8]. The study had to be conducted in humans and report on compounds that could be detected in biosamples following consumption of a fermented food. Studies were excluded if the food being considered was not fermented or if it was unclear if the food was fermented; the route of exposure to the food was not oral consumption; the amount of food consumed was not well-documented (e.g., a gram amount, or categorization to distinguish the fermented food from other foods consumed was not provided); the study was conducted in animals or in vitro; compounds in biological samples that could represent food biomarkers were not described; the aim of the study was either to review nutrient bioactivity and nutritional status using the fermented food as a delivery matrix or to assess the impact of a fermented food on the bioavailability of another food/compound; the study focused on a compound, supplement, or extract rather than a whole food; or the study only investigated alterations on the composition of the gut or fecal microbiota. Review articles, case reports or short communications (e.g., comment, editorial, conference abstract), and articles in a language other than English were also excluded.

### Strategy to identify and select the most discriminant compounds

Since our goal in this review was to evaluate a combination of candidate biomarkers for fermented foods (both specific and non-specific), rather than a single specific biomarker, we slightly deviated from the assessment of validity for putative/candidate biomarkers that was previously proposed by Dragsted et al. [31]. Following the selection of relevant full-text articles for inclusion, a series of steps were applied to select the most discriminant candidate biomarkers from the literature search. These included compounds that were highly discriminant for (i) the food (“food-level” biomarkers — i.e., FIBs specific for the intake of a particular food), (ii) food group (“food group-level” biomarkers — i.e., FIBs specific for the intake of a group of foods with a common raw material substrate or characteristic), or (iii) a dietary pattern of fermented food consumption (“fermentation-dependent” biomarkers — i.e., FIBs arising from the fermentation process of a food), from other non-fermented foods and food groups. In order to capture both specific markers that can discriminate the intake of a fermented food as well as non-specific markers that may act in combination to discriminate the intake of a fermented food, we firstly focused on summarizing in detail the compounds that were identified in discovery-driven “untargeted” studies that typically employed metabolomics tools (58 articles), and supplemented this information with “targeted” studies investigating a particular compound or set of compounds (243 articles), analyzed using metabolomics tools or other biochemical assays. Information from the untargeted studies was expected to identify biomarkers associated with a dietary pattern of consuming fermented foods, while information from both the untargeted and targeted studies was expected to help to further identify and verify biomarkers at the food level (e.g., cheese) and food group level (e.g., fermented dairy).

Compounds were selected if they were statistically significantly increased following consumption of the fermented food compared to baseline or control, and/or have been detected in multiple studies. For these selected compounds, we further consulted the study text to assess the biological plausibility, along with previous FIB reviews for their validation status. In addition, three food/metabolite databases (HMDB, Exposome-Explorer, FooDB) were searched in May 2019 and updated in September 2020 as an additional step for verifying that a compound appearing in a biosample has a food origin (or was transformed during metabolism) and to check the specificity of the compound for a fermented food. Information from food databases and the wider literature were also used to identify and confirm metabolites of fermentation. Compounds that were not discriminative of these classifiers but were associated with the fermented food or food group (e.g., detected but not significantly increased in biosamples following consumption), or compounds which have a ubiquitous presence across many other non-fermented foods, were not selected and not further discussed.

## Results and discussion

### Database search

**Fig. 2:**
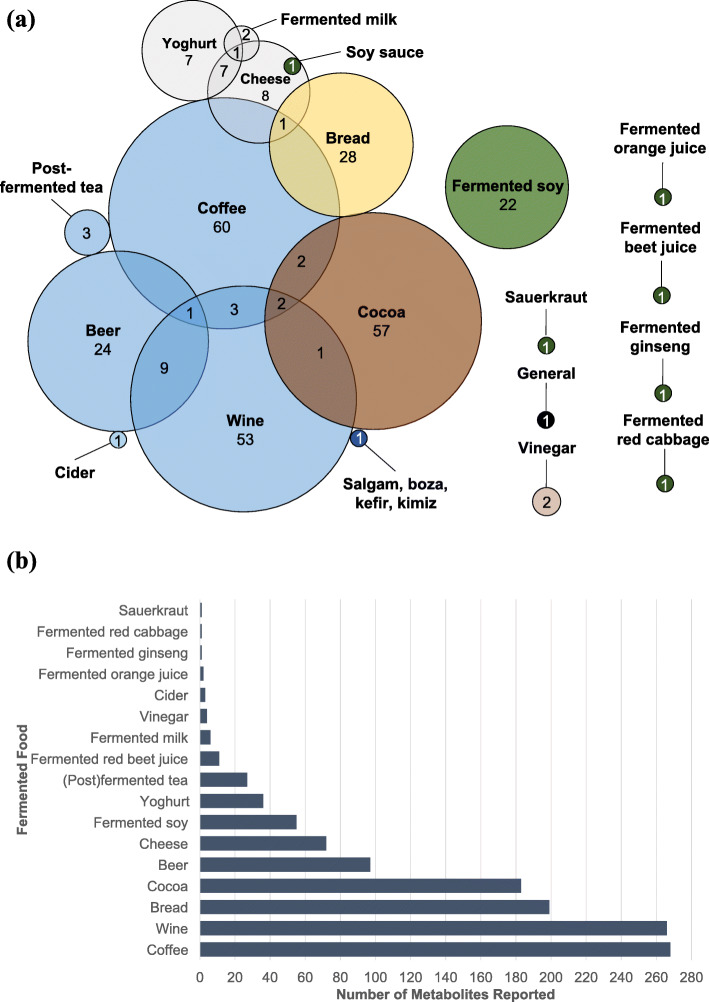
Overview of 301 included publications from the systematic literature search. **a** Number of publications identified for each type of fermented food (colored by food group). No articles were identified for fermented meat or fish products. **b** Number of identified metabolites reported in the included articles for the fermented food described

From the initial primary database search, a total of 11,764 records were identified, of which 7686 unique entries remained following the removal of duplicates (Fig. 1). After filtering the 7686 titles, 4890 were excluded and 2794 were deemed relevant for further review and their abstracts were retrieved. Following abstract review, 473 relevant entries remained, and their full-text articles were retrieved (2323 were excluded for various reasons outlined in the exclusion criteria). Further application of exclusion criteria to full-text articles (202 articles removed), and an updated search (30 articles added), resolved in 301 relevant full-text articles with information on compounds associated with intake of various fermented foods. The fermented foods investigated in the *n* = 301 studies were coffee (*n =* 69), wine (*n =* 69), cocoa (*n =* 62), beer (*n =* 34), bread (*n =* 29), fermented soy (*n =* 22), cheese (*n =* 18), yoghurt (*n =* 15), fermented milk (*n =* 3), post-fermented tea (*n =* 3), vinegar (*n =* 2), cider (*n =* 1), traditional Turkish beverages (salgam, boza, kefir, and kimiz) (*n =* 1), fermented orange juice (*n =* 1), fermented ginseng (*n =* 1), fermented beet juice (*n =* 1), fermented red cabbage (*n =* 1), soy sauce (*n* = 1), sauerkraut (*n* = 1), and general fermented products (*n =* 1) (Fig. 2a). The numbers of identified metabolites reported for each food across these studies are presented in Fig. 2b, and detailed lists of all of the included articles are presented in Additional File 3 (untargeted studies) and Additional File 4 (targeted studies). No studies reported on potential FIBs for fermented meat or fish products. Biological samples in which putative FIBs were identified or measured included serum (14 untargeted, 28 targeted studies), plasma (15 untargeted, 120 targeted studies), whole blood (13 targeted studies), urine (33 untargeted, 125 targeted studies), feces (5 untargeted, 7 targeted studies), ileal fluid (5 targeted studies), subcutaneous adipose tissue (2 targeted studies), oral fluid (3 targeted studies), plasma lipoproteins (2 targeted studies), erythrocytes (2 untargeted studies), capillary blood (1 targeted study), breast milk (1 targeted study), hair (1 targeted study), and breath (1 targeted study). The majority of studies were postprandial intervention studies (*n* = 183). The remainder comprised short-term and long-term intervention studies (*n* = 83) and observational studies where participants followed their habitual diet (where the diet was assessed by self-report tools such as FFQ, recall, food record, or dietary history) (*n* = 53).

From these relevant publications, compounds that could represent FIBs for various fermented foods are discussed based on their classification into three categories: food-level, food group-level, and fermentation-dependent FIBs (as described in the “Methods” section). An overview of the main FIBs identified, selected, and classified in this search is provided in Table 1. Although the aim of this review was not to identify a complete list of food-level or food group-level biomarkers, their inclusion in this review alongside fermentation-dependent markers provides the basis to help to facilitate a so-called multi-marker approach in estimating fermented food intake. Such a multi-marker approach could help confirm fermented food intake, or help distinguish between the intake of fermented and non-fermented versions of the same food.

**Table 1:** Candidate FIBs identified for various fermented foods from the systematic literature search

Fermented food(s)	Discriminant compounds/candidate biomarker level^**a**^				
	Food-level	Food group-level^**b**^		Fermentation-dependent	
**Wine**	• Tartaric acid/tartrate • Resveratrol and metabolites (trans-piceid, glucuronides and sulfates)	• (Epi)catechin and metabolites (also see cocoa, coffee, tea)		• Ethanol • Ethyl glucuronide • Ethyl sulfate	• Mannitol • Gallic acid
**Beer**	• (Iso)xanthohumol • Iso-alpha-acids (isohumulones) • 8-Prenylnaringenin	• Alkylresorcinols and metabolites (3,5-dihydroxybenzoic acid, DHPPA, C17:0 to C21:0 ratio) • Benzoxazinoids and related compounds (2-hydroxyl-1,4-benzoxazin-3-one, hydroxylated phenylacetamides and derivatives, HHPAA glucuronide and sulfate)	• Ferulic acid, dihydroferulic acid, and derivatives		• 2-Ethyl malate
**Bread**	• 2,4-Dihydroxybutanoic acid* • 2,8-Dihydroxyquinoline glucuronide*			• Methionine	
**Cocoa**	• None identified	• Caffeine and metabolites (theophylline, 1-methylxanthine, 3-methylxanthine, 7-methylxanthine, paraxanthine, theobromine, AAMU, AMMU) • 1-, 3-, or 7-Methyluric acid, 1,3-, 1,7-, or 3,7-dimethyluric acid, 1,3,7-trimethyluric acid • Chlorogenic acid, caffeic acids, quinic acids • Nicotinic acid, hydroxynicotinic acid • (Epi)catechin, (epi)catechin glucuronide and metabolites (3-hydroxyhippurate, MHPV, MHPV sulfate, glucuronide, 4-hydroxy-5-(3,4-dihydroxyphenyl) valeric acid, 4-hydroxy-5-(hydroxyphenyl) valeric acid sulfate, DHPV glucuronide, sulfoglucuronide)		• Acetate/acetic acid	
**Coffee**	• Trigonelline • N-methylpyridinium • Cyclo(isoleucyl-prolyl) • Atractyligenin glucuronide • 2-Furoylglycine • 4-Ethylguaiacol • 4-Vinylguaiacol				
**Tea**^**c**^	• C-linked dihydrochalcone and flavanone glucosides			• Theabrownins • Gallic acid	
**Soy**^**c**^	• None identified	• Pinitol • Isoflavones (daidzein, genistein, glycitein), glycoside-enriched		• Aglycone-enriched isoflavones and certain 4′ and 7′ isoflavone metabolites • Threonine, Tryptophan, Tyrosine, Valine • Vitamin B12 • Indole-3-lactic acid	• 4-Methylspinacemine • Menaquinone-7 (vitamin K2)
**Cheese**	• Isovalerylglutamic acid • Isovalerylglycine • Triglylglycine • Isobutyrylglycine	• Pentadecanoic acid (C15:0) • Heptadecanoic acid (C17:0) • 10Z-Heptadecenoic acid (C17:1) • Myristoyl-sphingomyelin SM(d18:1/14:0) • Lactose • Galactitol • Galactonate • Galactono-1,5-lactone • Galactose		• 3-Phenyllactic acid • Methionine	
					• Lactic acid
**Yoghurt**	• None identified			• Indole-3-lactic acid, indole-3-acetaldehyde, indole-3-propionic acid	

*AAMU* 5-acetylamino-6-amino-3-methyluracil, *AMMU* 5-acetylamino-6-formylamino-3-methyluracil, *DHPV* 5-(3,4-dihydroxyphenyl)-γ-valerolactone, *DHPPA* 3-(3,5-dihydroxyphenyl)-1-propanoic acid, *HHPAA* 2-hydroxy-N-(2-hydroxyphenyl) acetamide, *LAB* lactic acid bacteria, *MHPV* 3′-methoxy-4′-hydroxyphenylvalerolactone

^a^Wherever possible, the raw material from which the metabolite is derived from, the chemical class, or the fermentation or metabolic process by which the metabolite is generated from, is indicated in square brackets. A full list of references from which these metabolites were derived is provided in Additional Files 3 and 4. The specificity of food-level FIBs for each fermented food (or raw material) was verified through food database searches. Where specificity could not be confirmed, the metabolite is marked with a “*” and further expanded upon in the text

^b^A group of foods with a common raw material substrate or characteristic

^c^Post-fermented tea and fermented soy products

### Food-level biomarkers

Due to the overlapping compositional profiles of many foods, identification of specific FIBs for individual foods is challenging. In this review, food-level biomarkers were identified for beer, bread, wine, coffee, cheese, and fermented (rooibos) tea (Table 1). These compounds were largely derived from the (unfermented) raw materials. For example, isoxanthohumol, 8-prenylnaringenin, and iso-alpha-acids originate from beer hops that are used in the brewing process; tartaric acid and resveratrol are found at high concentrations in the grapes used for wine production [45–48]; and trigonelline and 2-furoylglycine originate from coffee beans and the coffee roasting process [49, 50]. For bread, the organic acids 2,4-dihydroxybutanoic acid and 2,8-dihydroxyquinoline glucuronide were identified following the intake of fermented sourdough endosperm rye and white wheat bread [51]. While these organic acids have seemingly not yet been detected/quantified in other foods (from food database searches), future validation would be useful in determining their usefulness as specific biomarkers for bread intake.

Food-specific biomarkers for fermented dairy products (cheese, yoghurt, buttermilk) [34], coffee [33], and cocoa products [32, 52] have also been the subject of previous systematic reviews. Notably, isovalerylglutamic acid, isovalerylglycine, triglylglycine, and isobutyrylglycine were previously identified as specific FIBs for cheese [34]. A large number of phenolic acid, alkaloid, and terpene derivatives have been identified as FIBs of coffee, with trigonelline and cyclo(isoleucylprolyl) emerging as the most specific biomarkers [33]. No specific biomarkers were identified, previously or in the current review, for yoghurt, buttermilk, or cocoa; however, several non-specific biomarkers at the food group level were found.

### Food group-level biomarkers

A number of FIBs previously proposed as food-specific markers have been re-classified as discriminant for a group of foods in light of evolving research. For instance, while caffeine has been consistently linked to coffee intake, it is also detected at fairly high concentrations in tea and chocolate [33, 52]. In addition, a growing range of products can be artificially caffeinated, which also obscures the use of caffeine as a FIB for only naturally caffeinated foods. Similarly, the hydroxycinnamate ferulic acid has been detected in high levels in coffee [53], but it is also an antioxidant that is ubiquitously found in plant tissues [54]. Unsurprisingly, increased levels of ferulic acid and its derivatives have been detected in blood and urine following the consumption of multiple plant-based foods [55, 56], indicating that the sole use of this compound as a FIB for coffee would be inappropriate. These compounds however may still be useful as food group-level biomarkers in conjunction with food-level and/or fermentation-dependent biomarkers for evaluating the intake of fermented foods.

The biomarkers identified at the food group level for different fermented foods are summarized in Table 1 and their relevance discussed below. It is important to note that while we defined “food groups” in the literature search by those conventionally used in dietary recommendation guidelines, food group-level biomarkers may be common across multiple foods based on common substrates of fermentation (e.g., wheat in both beer and bread production), or multiple biomarkers may apply in the case of multiple substrates for a single fermented food (e.g., tarhana, a fermented mixture of cereals and yoghurt). Since fermented beverages encompass a broad, heterogenous group, different fermented beverages are discussed in the context of their fermentation raw material, which includes milk (fermented milks, kefir, and yoghurt-based drinks), fruits (cider, wine, fermented orange juice, fermented beet juice), cereals and grains (beer), and others (coffee, post-fermented tea). In many cases, compounds identified at the food group level represented FIBs of unfermented raw material rather than a fermented food group, but nonetheless, their description is important as part of a combined model of fermented food intake.

#### Fermented dairy

Several compounds identified in our search were associated with the intake of cheese and yoghurt, including the widely discussed fatty acids heptadecanoic acid (C17:0) and pentadecanoic acid (C15:0). These fatty acids were also captured in the systematic review of egg and dairy biomarkers by Munger et al. [34] as dairy biomarkers, where additional FIBs were proposed for general dairy fat/dairy products, including 10Z-heptadecenoic acid (C17:1), myristoyl-sphingomyelin SM(d18:1/14:0), and galactonate. A handful of additional compounds that could represent biomarkers for milk (as compared to fermented milk) were additionally identified in the current search, including galactitol, galactonate, galactono-1,5-lactone, galactose, and lactose [57–59]. Collectively, these compounds represent FIBs that may be useful for estimating total dairy intake, including both fermented and non-fermented dairy. As the degree of transformation of lactose (and similar metabolites) greatly varies among dairy products, the profile of these combined metabolites could provide specific insights into the degree of fermentation of the ingested dairy products.

It has been reported that fermentation of milk products may increase the bioavailability of nutritionally important and bioactive compounds of milk [60]. Major milk proteins include caseins (αs1, αs2, β, and k), β-lactoglobulin, α-lactalbumin (precursor of serotonin), immunoglobulins (IgA, IgG, IgM), glycomacropeptide, lactoferrin, lactoperoxidase, lysozyme, and serum albumin [7]. Milk proteins are easily hydrolyzed to free amino acids during fermentation, and a large group of amino acids (alpha-amino butyric acid, alanine, asparagine, cysteine, glycine, glutamine, histidine, isoleucine, leucine, lysine, methionine, ornithine, phenylalanine, proline, serine, threonine, tryptophan, tyrosine, and valine) were found to be increased in plasma following yoghurt and cheese intake compared to control (milk or water) [61–63]. Dairy proteins are also a source of bioactive peptides that can be released during fermentation or during digestion [64, 65]. The bioactive peptides derived from these milk proteins during fermentation, such as Isoleucine-Proline-Proline (IPP) and Valine-Proline-Proline (VPP), are reported to possess antimicrobial, antioxidative, immunomodulatory, angiotensin-1-converting enzyme (ACE-1) inhibitory, and renin inhibitory activities [7]. While these peptides were not identified in our search, their presence in fermented dairy products warrants further investigation in a combination biomarker approach for this food group.

#### Fermented cereals and grains

Cereals and grains are a staple agricultural product around the world, and their fermentation results in an array of rice-based (idli, dosa), wheat-based (bread, kishk), corn-based (ogi, pozol), or sorghum-based (injera, kisra) dishes [66]. To date, the vast majority of research activity on FIBs of this fermented food group has centered around wheat-based bread products (whole or refined grain) that are leavened with baker’s yeast (*Saccharomyces cerevisiae*), with little to no reports on FIBs for other fermented grains. In the current review, alkylresorcinols and their primary metabolites 3,5-dihydroxybenzoic acid (DHBA) and 3-(3,5-dihydroxyphenyl)-propanoic acid (DHPPA), as well as benzoxazinoids and their metabolites (2-hydroxyl-1,4-benzoxazin-3-one, hydroxylated phenylacetamides and derivatives thereof), were identified as FIBs of wholegrain wheat/rye [67–72]. Since these compounds are derived from wholegrain wheat and rye, they are present at a higher abundance in biosamples following consumption of wholegrain breads, rather than refined-wheat bread [67]. A recent review focusing on mass spectrometry analysis of whole grains revealed the presence of hundreds of molecules in various wheat, barley, oat, and rye products, including alk(en)ylresorcinols, benzoxazinoids, avenanthramides, flavonoids, lignans, phytosterols, carotenoids, phenolic acids (hydroxybenzoic and hydroxycinnamic acids), sphingolipids, tocols, and glycine betaine [73]. While these compounds have been primarily reported in raw grains and leavened bread products, they may also be useful as FIBs for fermented food products in which grains are used as a starting raw material (e.g., wheat/barley in beer production). It has not yet been investigated whether these compounds can also be detected in soy sauce, which is a fermented mixture of soybeans and wheat [66].

A further distinction should also be made for breads that are leavened solely by yeast, and sourdough breads, which are both leavened by yeast and fermented by LAB. Sourdough-fermented rye has also been shown to contain higher levels of organic acids compared to rye bread, which can reduce starch digestibility and gastric emptying rate, leading to reduced insulin and glucose responses [74, 75]. In one study, consumption of sourdough fermented bread increased total free amino acids in plasma compared to bread fermented solely with yeast, indicating improved digestibility of protein [76].

#### Fermented meats and fish

Fermented meat products are broadly produced and consumed in Germany, France, Spain, Italy, the Balkans, Hungary, Australia, the USA, and Japan [7]. Despite their widespread consumption, no studies were identified in the current search that reported on candidate FIBs of fermented meat or fish. However, a number of studies have identified FIB of raw or unfermented meat and fish products. For example, a study in free-living individuals previously identified candidate biomarkers for chicken (anserine), meat (chicken, red meat, processed meat) (carnosine), fish (trimethylamine-N-oxide), and meat and fish intake (3-acetylcarnitines, including acetylcarnitine, propionylcarnitine, and 2-methylbutyrylcarnitine) [77]. In another study, 1- and 3-methylhistidine were determined to be urinary biomarkers for meat intake [78]. Furthermore, raw meat is known to contain the histidyl dipeptides, carnosine and anserine m[79]. FIBs for meat intake were comprehensively evaluated in a review, in which urea, creatine, creatinine, carnitine, carnosine, anserine, ophidine, 1- and 3-methylhistidine, and sulfate or sulfite were described as the most discriminant compounds [80].

For fermented meats, nitrites that are used as curing agents might also be present in some final products [81]. In addition, some fermented sausages have been reported to contain high levels of the biogenic amine tyramine [82] and the antioxidant taurine (2-aminoethane sulfonic acid) [79, 83], both of which warrant confirmation as FIBs for fermented meat in human studies. Similarly to fermentation of other high-protein foods, fermentation of meat products also releases bioactive peptides from proteolytic protein degradation. ACE-1 inhibitory peptides and antioxidant peptides have been identified in cured ham and fermented sausages, such as Serbian Petrovac sausage [84] and Spanish dry-cured ham [85–87]. While detected in the foods themselves, no studies were identified in the literature search in which these peptides were identified in biosamples following consumption of fermented meat products. On the other hand, biogenic amines [88] as well as ACE-1 inhibitory peptides [89] are well described in cheese, indicating that the distribution of these molecules extends beyond fermented meat products.

#### Fermented fruits and vegetables

While, in theory all fruits and vegetables could be fermented, those most commonly fermented include cabbage (sauerkraut, kimchi), cucumbers, olives, onions, carrots, caper berries, and garlic [90, 91]. Fruits and vegetables are commonly fermented using LAB and yeasts via techniques such as dry salting or storage in a brine [90]. During the lactic fermentation of cucumbers, cabbage, and olives, glucose and fructose are broken down to produce lactic acid, acetic acid, ethanol, and carbon dioxide [91]. The production of organic acids plays a critical role in food safety by limiting the growth of pathogenic microorganisms [90]. Slight differences in the fermentation process can also alter the final metabolite composition and quantities between food products. For instance, fermentation of cabbage into sauerkraut degrades glucosinolates to isothiocyanates, indole-3-carbinol, goitrin, allyl cyanide, and nitriles. While the degradation products allyl isothiocyaniate, allyl cyanide, and goitrin were higher in the spontaneously fermented product consisting of salted raw cabbage, methyl isothiocyanate and indole-3-carbinol were higher following in sauerkraut fermented with a starter culture containing LAB [92].

Plasma β-cryptoxanthin and lutein have been previously proposed as robust biomarkers for general fruit and vegetable intake [93] and have been used to measure dietary compliance in multiple human intervention studies. Untargeted metabolomics studies have further revealed a wide range of compounds associated with the intake of plant-based foods [94]. However, from our systematic search, only five studies investigating fermented fruits and vegetables were identified. In one study, D-phenyllactic acid, a LAB metabolite, increased in the serum and urine of four volunteers following acute consumption of sauerkraut [95]. In another study, 20-O-beta-D-glucopyranosyl-20(S)-protopanaxadiol, a novel ginseng saponin metabolite, was increased following intake of fermented ginseng. However, it was reported that the formation of this compound is likely attributable to the action of human intestinal bacteria [96]. Further, it was reported that fermented red cabbage has lower bioavailability of anthocyanins compared to fresh red cabbage [97]. Contrary results were reported in Hornero-Mendez et al. [98], where bioavailability of beta-cryptoxanthin and lutein (both attributed to oranges) were higher following consumption of fermented orange juice, and in Sawicki et al. [99], where increased levels of betalain and derivatives in plasma and urine following the consumption of fermented red beet juice were all attributed to red beetroot. However, despite the higher bioavailability afforded by the fermented products, high intake of unfermented forms of these foods would greatly obscure their use as FIBs in dietary assessment.

#### Fermented legumes and soy

Although the current search focused on identifying FIBs for all fermented legumes including soy products, only studies on fermented soy products were identified. Soybean products are commonly produced and consumed in East and Southeast Asia and West Africa [4]. Plasma and urinary isoflavones have long been used as markers of soy exposure [100, 101], and more recently, pinitol was identified as a candidate biomarker of soy intake in an untargeted metabolomics study [59]. Although most soy products are characterized by their isoflavone content (which are also present at moderate levels in other legumes), fermented soybeans are comparatively richer than non-fermented soybeans in the isoflavone genestein, as well as gamma-polyglutamic acid (PGA) which is produced by some strains of *Bacillus subtilis* during fermentation [4]. In addition, the natural isoflavones present in soybeans and unfermented soy products are glucose-conjugated and converted to the aglycone-isoflavones following hydrolysis during digestion prior to absorption [102]. Aglycone-enriched isoflavones that are present in fermented soy products have been reported to be more efficiently absorbed and therefore more bioavailable [102]. In a study by Jang et al. [103], comparing levels of soy isoflavones following ingestion of test meals containing fermented or unfermented soybean, the metabolites daidzein 7-O-glucuronide-4′-O-sulfate and genistein 4′,7-di-O-glucuronide were significantly higher in plasma, and genistein 7-Osulfate, glycitein 7-O-glucuronide-4′-O-sulfate, and genistein 4′-O-sulfate were significantly higher in urine, following fermented soy consumption, indicating these metabolites may be useful in distinguishing soy products with different fermentation status. In another acute intervention study, it has been demonstrated that fermentation of soybean increases the urinary recovery of soy isoflavones by 52% [104]. Analysis of several fermented soy products, including Chungkookjang, tempeh, doenjang, and miso, revealed higher levels of isoflavones (genistin, daidzin, glycitin, genstein, daidzein) and/or amino acids (in particular glutamate) compared to unfermented soybean [105]. In addition to soy isoflavones and aglycones, vitamins B2 and B12, and gamma aminobutyric acid (GABA), are increased in fermented soy products [106], and a variety of bioactive peptides have been identified, such as F2-2-2 and Fr-2-3 in chungkujang, Arginine-Proline in doenjang, Phenylalanine-Isoleucine-Glycine (1:2:5) in dou-chi, and Valine-Proline-Proline and Isoleucine-Proline-Proline in miso paste containing casein [107–109]. While many of these compounds are present across other foods as well (e.g., vitamin B12), which limits their usefulness as FIBs for fermented soy intake, their combination in a multi-marker approach warrants investigation.

#### Other fermented products

Coffee, tea, and chocolate are consumed worldwide, but largely unbeknownst to consumers as “fermented” food products. Unlike yoghurt and cheeses, where the final food products are subject to fermentation and are typically carriers of live microorganisms, fermentation of coffee, tea, and cocoa occurs upstream in the food manufacturing process [110]. Following their harvest, cocoa seeds are intentionally fermented for 7 days [111, 112], raw coffee berries for 10 to 25 days [113], and in the case of post-fermented teas, fresh tea leaves may be fermented from several months up to several years [114]. These foods rely on spontaneous fermentation via the actions of endogenous microbes, and depending on the duration and conditions of the fermentation, different compositional and flavor profiles are attained.

Along with the food-level biomarkers identified for coffee, post-fermented tea, and cocoa as described above, our systematic search revealed several overlapping candidate biomarkers for these foods based on a common raw material characteristic other than a shared substrate. These included caffeine and its metabolites (theobromine, theophylline, methylxanthines, methylurates), nicotinic acid, and multiple phenolic acids, including (epi)catechin, chlorogenic acid, caffeic acids, and quinic acids [94, 115–118] (Table 1). Polyphenols are a group of chemically diverse compounds with high abundance in the diet [115, 116]. Despite the widespread prevalence of polyphenols in a variety of plant-based foods (i.e., coffee, wine, citrus, apples, pears, tea, chocolate), which renders them non-specific biomarkers, distinct polyphenols have been shown to be more closely associated with certain foods than others. For example, methyl-(epi)catechin sulfate has been associated with chocolate intake, hydroxytyrosol, resveratrol, and gallic acid with red wine intake, and (dihydro)ferulic acid and caffeic acid with coffee intake [115, 116]. Quantification of these polyphenols in biofluids may assist in determining cut-offs or ratios as an indication of their usefulness as biomarkers of acute or habitual intake of these foods. Furthermore, enterolactone, a phytoestrogenic compound formed via gut microbial transformation of plant lignans, has been detected in the blood or urine of individuals following consumption of breads, cocoa, coffee and tea, and soy products. The non-specific nature of this compound limits its usefulness as a specific FIB, but may be interesting to explore as a food group-level biomarker.

### Fermentation-dependent biomarkers

Fermentation of foods is used in part to improve the bioavailability of dietary compounds, or release novel metabolites generated via microbial enzymes [8]. These metabolites that can be considered as potential fermentation-dependent FIBs associated with a dietary pattern of fermented food consumption have not been previously documented in a systematic manner. In this review, we identified several compounds that arise from the fermentation process of a particular food, food group, or different fermented foods possibly indicating fermentation with common microbes.

Several of the potential FIBs identified in this search correspond to specific features of the type of fermentation process or the food that is fermented. Notably, the presence of high levels of the sugar-alcohol mannitol in wine is indicative of fructose degradation during fermentation with LAB [29, 119, 120], while 2-ethylmalate detected in beer is indicative of yeast fermentation [121]. A significant increase in methionine following sourdough bread [51] and cheese consumption [58] is in line with previous reports of methionine detected in fermented foods, and methionine (and lysine) production by some cultures of *Lactobacillus* and yeasts used in the fermentation of cereals [122, 123].

For tea, an increase in theabrownins (phenolic pigment compounds) reflects the fermentation of catechins and gallate derivatives. Along with acting as a possible FIB for the fermentation process, theabrownins may serve a dual role as health biomarkers as well, as it was recently demonstrated that theabrownins from post-fermented pu-erh tea exerts cholesterol- and lipid-lowering effects via modulation of gut microbiota and bile acid metabolism [124]. Furthermore, increased levels of gallic acid for tea and wine result from the fermentation of the polyphenol EGCG, which is a common food group-level metabolite for these foods [125, 126].

Similarly, as a major byproduct of alcoholic fermentation with yeast, ethanol and its metabolites (e.g., ethyl glucuronide, ethyl sulfate) could be considered fermentation biomarkers for alcoholic beverages such as wine, beer, and distilled liquor [127]. Ethanol has been widely used by food and forensic scientists alike to detect and monitor levels of alcohol, typically in blood or expired breath. However, ethanol was not increased in blood following consumption of Şalgam, boza, kimiz, or kefir, which have low alcoholic content due to mainly being fermented with LAB [128]. As such, the utility of ethanol as a biomarker may not extend to low-alcohol beers, dealcoholized wine, or similar variations of these beverages, due to differences in the fermentation process (e.g., selection of yeast strains that do not consume or produce ethanol) or the removal of alcohol from the fermented product.

LAB are used for the fermentation of many food substrates [129], and several classes of compounds are produced via lactic fermentation processes. During fermentation, LAB can convert amino acids into amine-containing compounds referred to as biogenic amines [130], which can be detected at fairly high concentrations in the final fermented foods. Fermented sausages, for example, have been reported to contain high concentrations of biogenic amines (spermine, spermidine) since their production is primarily attributed to the action of decarboxylase-positive bacteria and meat enzymes during fermentation and ripening [131]. Biogenic amines serve a critical physiological role as precursors for the synthesis of hormones, alkaloids, nucleic acids, and proteins; act as neurotransmitters; and play a role in other central biological functions [132, 133]. While accumulation of biogenic amines in the body has toxicological consequences [130], moderate levels are generally detoxified by amino oxidases in the gut. Despite extensive reports describing the presence of biogenic amines in fermented foods such as cheese, fermented vegetables, wine, and fermented meats, in the current search, only one study reported an increase in spermidine levels (fecal) following yoghurt consumption for 2 to 4mweeks [134, 135], and it is unknown whether this increase is a result of food consumption or synthesis by the gut microbiota. A review of biogenic amines in food products further indicates that biogenic amines are also naturally present in grapes, raw meat and seafood, and fresh milk [136], which offers an explanation of why biogenic amines have not served a prominent role as FIBs for fermented foods. Additionally, biogenic amines are notoriously chemically unstable, as well as light- and pH-sensitive, which makes their analysis difficult [137]. However, some research has indicated that further chemical reactions of indoleamines with acetaldehyde can produce novel metabolites during fermentation, ripening, and storage that could be more specific for fermented foods. In a study by Ohya et al. [138], 4-methylspinacemine and its metabolite, 1,4-dimethylspinacemine (Pictet-Spengler condensation reaction products of histamine with acetaldehyde), were increased in the urine of volunteers following consumption of soy sauce (with a meal) or Appenzeller cheese. Analysis of various fermented foodstuffs, including soy sauce, fish sauce, cheese, and shao hsing wine, confirmed the presence of both compounds in these foods [138].

A number of vitamins such as folate, vitamin B12, riboflavin, and vitamin K are produced from fermentation of dairy products, elevating the nutritional quality of the product [7]. In particular, many foods fermented using *B. subtilis* give rise to menaquinone 7 (MK-7, or vitamin K2), which is a long-chain menaquinone primarily synthesized by bacteria and detected abundantly in cheese, as well as fermented soybean products. However, MK-7 can also be synthesized by the gut microbiota, indicating a dual exogenous/endogenous origin of this compound [139]. In the current search, increased MK-7 in serum or plasma was reported following consumption of the fermented soy product, natto [140–143], and validated in cross-sectional studies based on the frequency of natto consumption [141, 143].

Indoles, metabolites derived from tryptophan which act as endogenous ligands for the aryl hydrocarbon receptor, are also known to be produced from LAB via the tryptophanase pathway [144]. In the current review, indoles (especially indole-3-lactic acid) have been detected in biosamples following the consumption of multiple fermented foods, including yoghurt, cheese, beer, coffee, and bread. In addition, multiple strains of LAB produce phenyllactic and 4-hydroxyphenyllactic acids, and these metabolites have been shown to play a role in the quality and preservation of foods [145]. D-phenyllactic acid was increased in serum and urine following the acute consumption of Gruyère cheese [58, 59] and in plasma and urine following the acute consumption of sauerkraut [95]. Given that D-phenyllactic acid has also been confirmed to be present in other LAB-fermented foods including kimchi and sourdough [146–148], further investigation is warranted for this metabolite as a promising “fermentation-dependent” FIB for lactic-fermented foods.

### Heterogeneity of fermented foods and impact on FIBs

An inherent challenge in searching for FIBs of fermented foods is attributable to the heterogeneity of this food group. As evident in this review, virtually all food substrates can be fermented, and differences in fermentation conditions, such as type of microorganisms involved, duration of fermentation, even minute changes in environmental conditions, further contribute to producing foods with vastly different compositional profiles. To illustrate, consider the fermentation of milk to produce different types of cheeses. The common starting substrate, milk, can originate from cows, goats, sheep, water buffalo, or a combination of these [149]. Some cheeses are ripened with internal (Grana Padano) or surface bacteria (Havarti, Limburger), others with internal molds (Roquefort) or surface (Brie, Camembert) molds [149]. Even within bacteria-ripened cheese, interestingly, the “holes” in the cheese are created via different processes: in Swiss Emmental cheese by fermentation of lactate by *Propionibacterium freudenreichii*, and in Dutch Gouda cheese by fermentation of citrate by LAB [149].

Aside from the use of different microorganisms, the abundance of microbes can vary widely, and in some cases, the microorganisms are intentionally removed (e.g., heat inactivation, filtration). Even in the absence of a heat or separation step, the number of microbes present at the time of consumption depends on multiple factors, such as the initial composition, storage conditions, and the age of the food [150]. In a review by Rezac et al. [151], levels of live microorganisms in fermented foods were found to be dependent on geographical region and age of the food. For instance, microbial counts were undetectable (< 10^3^ cfu/g) in Swiss Gruyère or Grana Padano cheeses aged greater than 1 year, while high counts (10^9^ cfu/g) were found in Tilsit cheese aged for 2 to 4 months [151]. Given that fermentation relies on the enzymatic activities of microorganisms to convert food components, the chances of fermentation-dependent metabolites being detected in biosamples to be identified as FIBs are inevitably linked to the amount of microorganism present in the food product. Furthermore, while industrial fermentations are typically conducted using predefined starter cultures to guarantee consistency, safety, and specific metabolic activities, artisanal fermentations (which are gaining in popularity) rely on mixed sources or microbes endogenous to the raw food [152]. This further complicates the generalizability of any FIBs identified for industrially fermented foods and necessitates careful documentation of fermentation procedures and metabolic products in all cases.

### Impact of gut microbiota on FIBs for fermented foods

A second complexity in exploring FIBs for fermented foods involves the food-gut microbiota interface. Many fermented foods can act as a delivery vehicle for live microorganisms that can subsequently contribute to a changed gut microbiota landscape and altered metabolite appearance [8, 22]. The diversity of microorganisms found in various fermented foods, as well as their functional properties, have been reviewed previously [22, 153]. Both the gut and food microbiota can “ferment” food components, and it has recently been documented that over 40% of the LAB consumed via the ingestion of fermented foods (mainly cheese and other fermented milk products) become members of the gut microbiome [152]. Interestingly, the species of LAB colonized in the gut was found to be regionally dependent, with *S. thermophiles* and lactobacilli linked to yoghurt and dairy product consumption in Western diets, and heterofermentative *Leuconostoc* and *Weissela* linked to fermented vegetables and cereal-based foods predominant in non-Western diets [152]. A further study by Taylor et al. [154] indicated that gut microbiome composition and functional profile are also affected by the frequency of consumption of fermented plants, with several microbes (*L. brevis*, *L. kefiranofaciens*, *L. parabuchneri*, *L. helveticus*, and *L. sakei*) associated with both fermented foods and self-reported “consumers,” but not “non-consumers” [154]. Furthermore, while transient or long-term intake of fermented foods may differentially impact the gut microbiome, the response of the microbiome to diet remains highly personalized [155]. Collectively, these reports reflect the challenge in delineating the origin of a FIB as from a fermented food, or from a non-fermented food transformed by the gut microbiota.

### Representation of fermented foods consumed globally in the literature

While our goal was to search for FIBs for fermented food products consumed globally, a small number of fermented food products were represented in the current literature. To date, the majority of research has concentrated on coffee, beer, wine, chocolate, bread, and fermented dairy products, as described above. Studies on fermented foods consumed in large quantities in Africa and Asia, for example products from rice (idli, dosa, dhokla), corn (ogi, kenkey, pozol), or sorghum (injera, kisra), fermented alcoholic beverages (sake, bouza, chichi, mahewu, boza) [3–5], were not identified, indicating a gap in the scientific literature. There exists a great opportunity for the further exploration and validation of biomarkers for less-commonly investigated fermented foods, the results of which will help benefit fermentation-dependent FIBs for fermented foods overall.

## Conclusions

The large number of different food-level, food group-level, and fermentation-dependent compounds identified in this literature search may be promising FIBs for fermented food products if combined in a multi-marker approach, and needs to be validated in free-living cohorts with uncontrolled diets. While fairly specific food-level and food group-level biomarkers exist for commonly consumed fermented foods (e.g., trigonelline for coffee, pentadecanoic acid for dairy), this review captured several fermentation-dependent FIBs common among foods fermented by the same fermentation process (e.g., ethanol and metabolites for alcoholic fermentation, methionine, indoles, and 3-phenyllactic acid from lactic fermentation). Further, several gaps in the literature were revealed, particularly in the lack of studies on FIBs for fermented meats, fish, fruits, and vegetables, which presents an opportunity for future scientific investigation. Expanding the repertoire of FIBs for different fermented food products will greatly aid epidemiological efforts aimed to associate fermented foods with various health outcomes.

## Supplementary Information

**Additional File 1.** Food-Specific Keywords Used in the Literature Search for Candidate Biomarkers of Fermented Food Intake.pdf.

**Additional File 2.** Operators Used in the Literature Search for Candidate Biomarkers of Fermented Food Intake.pdf.

**Additional File 3.** Summary of Untargeted Studies Presenting Candidate FIBS for Fermented Foods.pdf.

**Additional File 4.** Summary of Targeted Studies Presenting Candidate FIBs for Fermented Foods.pdf.

## Data Availability

Not applicable.

## Abbreviations

ACE-1: Angiotensin-1-converting enzyme
FIB: Food intake biomarker
FFQ: Food frequency questionnaire
LAB: Lactic acid bacteria
