# Supplementary material for: A systematic review to identify biomarkers of intake for fermented food products
Source: Genes Nutr. 2021 Apr 21;16:5. doi: 10.1186/s12263-021-00686-4 (PMC8058972; doi:10.1186/s12263-021-00686-4)
Supplement: Supplementary file 1 — Additional File 1. Food-Specific Keywords Used in the Literature Search for Candidate Biomarkers of Fermented Food Intake.pdf. [file 12263_2021_686_MOESM1_ESM.pdf]

Additional File 1

**Food-Specific Keywords Used in the Literature Search for Candidate Biomarkers of Fermented Food Intake**			
**Operator**	**Database**	**Field**	**Keywords**
**General Fermented Foods**			
AND	Pubmed	Title/Abstract	ferment* OR “fermented food*” OR “fermented product*”
	Web of Science	Topic	
	Scopus	Article Title/Abstract/Keywords	
**Fermented Dairy**			
AND	Pubmed	Title/Abstract	cheese OR yoghurt OR yogurt OR yoghourt OR yakult OR “creme fraiche” OR quark OR kefir OR lassi OR “sour cream” OR “soured milk” OR “cultured milk” OR “cultured dairy” OR “fermented dairy” OR “fermented milk” OR buttermilk
	Web of Science	Topic	
	Scopus	Article Title/Abstract/Keywords	
NOT	Pubmed	Title/Abstract	"breast milk" OR "breast feeding" OR bone OR muscle OR allerg* OR "phyto ster*" OR "plant ster*" OR phytoster* OR newborn* OR infant*
	Web of Science	Topic	
	Scopus	Article Title/Abstract/Keywords	
**Fermented Meat & Fish**			
AND	Pubmed	Title/Abstract	“fermented meat” OR salami OR pepperoni OR chorizo OR cervelat OR mettwurst OR “summer sausage” OR sucuk OR “fermented sausage” OR “cured meat” OR “dried meat” OR “dry sausages” OR “dried sausages” OR “processed meat” OR “fermented fish” OR “fish sauce” OR “shrimp paste” OR “shrimp sauce”
	Web of Science	Topic	
	Scopus	Article Title/Abstract/Keywords	
**Fermented Fruits & Vegetables**			
AND	Pubmed	Title/Abstract	“fermented vegetable*” OR “fermented fruit*” OR sauerkraut OR olive* OR pickle* OR “fermented cucumber*” OR kimchi OR paocai
	Web of Science	Topic	
	Scopus	Article Title/Abstract/Keywords	
NOT	Pubmed	Title/Abstract	oil* OR “olive leaf extract”
	Web of Science	Topic	
	Scopus	Article Title/Abstract/Keywords	
**Fermented Legumes (Including Soy)**			
AND	Pubmed	Title/Abstract	“fermented soy*” OR “fermented bean” OR “soy sauce” OR “soya sauce” OR “soybean paste” OR miso OR tempeh OR natto OR cheonggukjang OR doenjang OR doubanjiang OR douchi OR gochujang
	Web of Science	Topic	
	Scopus	Article Title/Abstract/Keywords	
NOT	Pubmed	Title/Abstract	milk OR allerg*
	Web of Science	Topic	
	Scopus	Article Title/Abstract/Keywords	
**Fermented Cereals & Grains**			
AND	Pubmed	Title/Abstract	“fermented cereal*” OR “fermented grain*” OR “fermented wheat” OR “fermented oat*” OR “fermented rice” OR bread* OR sourdough OR crispbread
	Web of Science	Topic	
	Scopus	Article Title/Abstract/Keywords	
**Fermented Beverages**			

AND	Pubmed	Title/Abstract	“fermented beverage*” OR “fermented drink*” OR beer OR wine OR cider OR kombucha OR pulque OR coffee OR “fermented tea” OR “dark tea” OR “yellow tea” OR puer OR pu’er OR pu-er* OR fuzhuan OR “fu zhuan”
	Web of Science	Topic	
	Scopus	Article Title/Abstract/Keywords	
**NOT**	Pubmed	Title/Abstract	poison* OR drug* OR smoking OR toxic*
	Web of Science	Topic	
	Scopus	Article Title/Abstract/Keywords	
**Other**			
AND	Pubmed	Title/Abstract	chocolate OR cocoa OR “fermented condiment” OR “vinegar” OR tabasco OR worcestershire OR worcester
	Web of Science	Topic	
	Scopus	Article Title/Abstract/Keywords	
