# Supplementary material for: A systematic review to identify biomarkers of intake for fermented food products
Source: Genes Nutr. 2021 Apr 21;16:5. doi: 10.1186/s12263-021-00686-4 (PMC8058972; doi:10.1186/s12263-021-00686-4)
Supplement: Supplementary file 2 — Additional File 2. Operators Used in the Literature Search for Candidate Biomarkers of Fermented Food Intake.pdf. [file 12263_2021_686_MOESM2_ESM.pdf]

Additional File 2

Operators Used in the Literature Search for Candidate Biomarkers of Fermented Food Intake			
**Operator**	**Database**	**Field**	**Keywords**
AND	Pubmed	All fields	biomarker* OR marker* OR metabolite* OR biokinetics OR biotransformation OR pharmacokinetics
	Web of Science	Topic	
	Scopus	Article Title/Abstract/Keywords	
AND	Pubmed	All fields	intake* OR meal* OR diet* OR ingestion OR consumption OR eat* OR drink* OR administration
	Web of Science	Topic	
	Scopus	Article Title/Abstract/Keywords	
AND	Pubmed	All fields	human* OR men OR women OR patient* OR volunteer* OR participant* OR individual* OR subject*
	Web of Science	Topic	
	Scopus	Article Title/Abstract/Keywords	
AND	Pubmed	All fields	urine OR plasma OR blood OR serum OR excretion OR tissue* OR faeces OR feces OR "fecal water" OR "faecal water" OR nail* OR hair
	Web of Science	Topic	
	Scopus	Article Title/Abstract/Keywords	
