# Supplementary material for: A systematic review to identify biomarkers of intake for fermented food products
Source: Genes Nutr. 2021 Apr 21;16:5. doi: 10.1186/s12263-021-00686-4 (PMC8058972; doi:10.1186/s12263-021-00686-4)
Supplement: Supplementary file 3 — Additional File 3. Summary of Untargeted Studies Presenting Candidate FIBS for Fermented Foods.pdf. [file 12263_2021_686_MOESM3_ESM.pdf]

Additional File 3

**Summary of Untargeted Studies Presenting Candidate FIBs for Fermented Foods**								
**Fermented Food**	**Intervention**	**Dose**	**Study Design**	***n***	**Analytical Method**	**Biosample**	**Candidate FIBs[a]**	**Reference**
Cocoa	New Nordic Diet vs. Average Danish Diet	15±7 g/day (derived from 3-day weighted food records)	6-month, randomized, controlled, parallel	107 healthy volunteers	U(H)PLC-QTOF-MS	Urine	• 7-Methyluric acid<br>• AMMU<br>• Theobromine	(1)
Cocoa	New Nordic Diet vs. Average Danish Diet	Exact intakes not reported (derived from 3-day weighted food records)	6 month, randomized, controlled, parallel; 3-day weighted food records (24h)	161 healthy adults	U(H)PLC-QTOF-MS	Urine	• **3,7-Dimethyluric acid**<br>• **7-Methyluric acid**<br>• **7-Methylxanthine**<br>• **AMMU**<br>• **Theobromine**	(2)
Cocoa	Cocoa or derived products	≥3x30 g/week chocholate and/or cocoa powder	Cross-sectional	64 healthy adults	HPLC-QTOF-MS	Urine	• **(Epi)catechin glucuronide**<br>• **(Epi)catechin sulfate**<br>• **3,7-Dimethyluric acid**<br>• **3-Methyluric acid**<br>• **3-Methylxanthine**<br>• **4-Hydroxy-5-(dihydroxyphenyl) valeric acid**<br>• **4-Hydroxy-5-(dihydroxyphenyl) valeric acid glucuronide**<br>• **4-Hydroxy-5-(dihydroxyphenyl) valeric acid sulfate**<br>• **4-Hydroxy-5-(hydroxy-methoxyphenyl) valeric acid sulfate**<br>• **4-Hydroxy-5-(hydroxyphenyl) valeric acid sulfate**<br>• **4-Hydroxy-5-(hydroxyphenyl) valeric acid sulfate**<br>• **4-Hydroxy-5-(phenyl) valeric acid sulfate**<br>• **7-Methylxanthine**<br>• **AMMU**<br>• **AMMU isomer**<br>• **Aspartyl-phenylalanine**<br>• **Cyclo(aspartylphenylalanyl)**<br>• **DHPV sulfoglucuronide**<br>• **DHPV glucuronide**<br>• **DHPV sulfate**<br>• **Furoylglycine**<br>• **Hydroxyphenyl-valerolactone glucuronide**<br>• **Hydroxyphenyl-valerolactone sulfate**<br>• **MHPV**	(3)

Summary of Untargeted Studies Presenting Candidate FIBs for Fermented Foods								
**Fermented Food**	**Intervention**	**Dose**	**Study Design**	***n***	**Analytical Method**	**Biosample**	**Candidate FIBs[a]**	**Reference**
							• **MHPV glucuronide**<br>• **Theobromine**<br>• **Vanillic acid**<br>• **Vanillin sulfate**<br>• **Xanthine**	
Cocoa	Cocoa powder in water vs. cocoa powder in milk	40 g/day + 250 mL water or milk	Acute, randomized, controlled, crossover	10 healthy adults	HPLC-QTOF	Urine	• **3,5-Diethyl-2-methylpyrazine**<br>• **3,7-Dimethyluric acid**<br>• **3-Methyluric acid**<br>• **3-Methylxanthine**<br>• **4-hydroxy-5-(3,4-dihydroxyphenyl) valeric acid**<br>• **7-Methyluric acid**<br>• **7-Methylxanthine**<br>• **AMMU**<br>• **Caffeine**<br>• **Cyclo(Pro-Pro)**<br>• **Cyclo(Ser-Tyr)**<br>• **DHPV glucuronide**<br>• **DHPV sulfate**<br>• **Epicatechin-O-sulfate**<br>• **Hydroxyacetophenone**<br>• **Hydroxynicotinic acid**<br>• **MHPV**<br>• **MHPV glucuronide**<br>• **O-Methylepicatechin**<br>• **Theobromine**<br>• **Trigonelline**<br>• **Tyrosine**<br>• **Tyrosine**<br>• **Vanillic acid**<br>• **Vanilloylglycine**	(4)
Cocoa	Cocoa powder in milk vs. milk only	40 g/day + 500 mL skimmed milk	8-week, randomized, controlled, crossover	20 patients at high risk of CVD	HPLC-QTOF	Urine	• **(Epi)catechin glucuronide**<br>• **N-[4-hydroxy-3 -methoxy-E-cinnamoyl]-L-aspartic acid**<br>• **N-[4-hydroxycinnamoyl]-L-aspartic acid**<br>• **Theobromine**<br>• **Vanillic acid glucuronide**<br>• **Vanillic acid sulfoglucuronide**<br>• **Vanilloylglycine**<br>• **3,5-Diethyl-2-methylpyrazine**<br>• **3,7-Dimethyluric acid**<br>• **3-Methyluric acid**<br>• **3-Methylxanthine**<br>• **4-Hydroxy-5-(dihydroxyphenyl) valeric acid glucuronide**	(5)

Summary of Untargeted Studies Presenting Candidate FIBs for Fermented Foods								
**Fermented Food**	**Intervention**	**Dose**	**Study Design**	***n***	**Analytical Method**	**Biosample**	**Candidate FIBs[a]**	**Reference**
							• **4-Hydroxy-5-(dihydroxyphenyl) valeric acid sulfate**<br>• **4-Hydroxy-5-(hydroxy-methoxyphenyl) valeric acid glucuronide**<br>• **7-Methyluric acid**<br>• **7-Methylxanthine**<br>• **AMMU**<br>• **Cyclo(propylalanyl)**<br>• **DHPV glucuronide**<br>• **DHPV sulfoglucuronide**<br>• **DHPV sulfate**<br>• **Epicatechin sulfoglucuronide**<br>• **Hydroxyphenyl-γ-valerolactone glucuronide**<br>• **Methyl(epi)catechin sulfate**<br>• **MHPV**<br>• **MHPV sulfate**	
Cocoa	Cocoa powder in milk	40 g/day + 250 mL milk	Acute intervention	10 healthy adults	HPLC-QTOF-MS	Urine	• 3-Methylxanthine<br>• Theobromine<br>• Vanilloylglycine<br>• Xanthurenic acid<br>• 7-Methylxanthine<br>• DHPV glucuronide<br>• Furoylglycine<br>• **N-methylguanine**	(6)
Cocoa	Chocolate (Flavan-3-ol-enriched dark, standard dark, white)	60 g + 400 mL still table water (200 mL in TO 2h and 4h)	Acute, randomized, controlled, crossover	42 healthy adults	NMR	Urine	• **2-Hydroxyisobutyrate**<br>• **3-Hydroxyisobutyrate**<br>• **3-Hydroxyisobutyrate**<br>• **4-Hydroxyphenylacetate**<br>• **Arginine**<br>• **Creatinine**<br>• **Alanine**<br>• **Dimethylamine**<br>• **Glycine**<br>• **Lactate**<br>• **N-acetylated compounds**<br>• **N-methylnicotinamide**<br>• **Pyruvate**<br>• **Theobromine**<br>• **Tyrosine**<br>• **Valine**<br>• **Epicatechin derivative**<br>• **Methylxanthines**	(7)
					LC-MS		• **Caffeine**<br>• **DHPV glucuronide**	

Summary of Untargeted Studies Presenting Candidate FIBs for Fermented Foods								
**Fermented Food**	**Intervention**	**Dose**	**Study Design**	***n***	**Analytical Method**	**Biosample**	**Candidate FIBs[a]**	**Reference**
							• **DHPV sulfate**<br>• **Epicatechin-O-sulfate**<br>• **Hydroxynicotinic acid**<br>• **MHPV glucuronide**<br>• **O-feruloylquinate**<br>• **Vanilloylglycine**<br>• **Epicatechin derivative**<br>• **Methylxanthines**<br>• **3,7-Dimethylurate**<br>• **4-hydroxy-5-(3,4-dihydroxyphenyl) valeric acid**<br>• **7- and 3-methyluric acid**<br>• **7- and 3-methylxanthine**<br>• **AMMU**	
Cocoa, wine	Chocolate	Habitual intake (dose not reported); assessed by FFQ	Prospective cohort	3559 adult female twins	U(H)PLC-MS/MS	Serum/plasma	• **7-Methylxanthine**<br>• **Theobromine**<br>• **1-Methylxanthine**<br>• **3-Hydroxypyridine sulfate**<br>• **3-Methyl catechol sulfate**<br>• **Catechol sulfate**<br>• **Cyclo(leu-pro)**<br>• **O-methylcatechol sulfate**<br>• **Quinate**	(8)
	Wine	Habitual intake (dose not reported); assessed by FFQ					• **1-docosahexaenoylglycerophospho ethanolamine**<br>• **2-aminobutyrate**<br>• **2-hydroxybutyrate**<br>• **2-Hydroxyisovalerate**<br>• **3-(4-hydroxyphenyl) lactate**<br>• **3-Methyl-2-oxobutyrate**<br>• **4-androsten-3beta,17beta-diol disulfate**<br>• **4-Methyl-2-oxopentanoate**<br>• **5-alpha-androstan-3beta-17beta-diol disulfate**<br>• **Arachidonate (20:4n6)**<br>• **Benzoate**<br>• **beta-hydroxyisovalerate**<br>• **Caprate (10:0)**<br>• **Caprylate (8:0)**<br>• **Docosahexaenoate (22:6n3)**<br>• **Docosapentaenoate (22:5n3)**<br>• **Eicosapentaenoate (20:5n3)**<br>• **Epiandrosterone sulfate**<br>• **myo-Inositol**<br>• **Phosphatidylcholine diacyl C32:1**<br>• **Phosphatidylcholine diacyl C36:5**	

Summary of Untargeted Studies Presenting Candidate FIBs for Fermented Foods								
**Fermented Food**	**Intervention**	**Dose**	**Study Design**	***n***	**Analytical Method**	**Biosample**	**Candidate FIBs[a]**	**Reference**
							• **Pipecolate**<br>• **Piperine**<br>• **Scyllo-inositol**<br>• **Stearidonate (18:4n3)**<br>• **Theophylline**	
Cocoa	Chocolate (80% cocoa)	100 or 200 g/day	2-day, randomized, controlled	15 heathy young adults	U(H)PLC-ESI-QTOF-MS	Urine	• Epicatechin-O-sulfate<br>• Epicatechin-O-glucuronide<br>• O-Methyl-epicatethin-O-sulfate<br>• O-Methyl-epicatethin-O-glucuronide<br>• O-Methyl-epicatethin-sulfate-O-glucuronide<br>• O-Methyl-epicatethin-disulfate-O-glucuronide<br>• Epicatechin-O-glucoside<br>• 5-(Hydroxyphenyl)-g-valerolactone-O-glucuronide<br>• 5-(Hydroxyphenyl)-g-valerolactone-O-glucuronide<br>• 5-Phenyl-g-valerolactone-glucuronide<br>• 5-Phenyl-g-valerolactone-methoxy-glucuronide<br>• 5-(Tri hydroxyphenyl)-g-valerolactone-glucuronide<br>• 5-(Hydroxy phenyl)-g-valerolactone-methoxy-sulfate<br>• 5-(Dihydroxy phenyl)-g-valerolactone-sulfate<br>• 5-Phenyl-g-valerolactone-methoxy-sulfate<br>• 5-(Hydroxyphenyl)-g-valerolactone-sulfate<br>• 5-Phenyl-g-valerolactone-sulfate<br>• 5-(Dihydroxy phenyl)-g-valero lactone	(9)
Cocoa	Dark chocolate (74% cocoa) consumption in volunteers with different anxiety traits	40 g/day	2-week, randomized, parallel, open	30 healthy volunteers	NMR, GC-MS, LC-MS/MS	Plasma, urine	• Asparagine<br>• Corticosterone<br>• Cortisol<br>• Cystine<br>• 4-Hydroxyphenylacetate<br>• Adrenaline<br>• Glucose-6-phosphate<br>• Normetanephrine<br>• Threonic acid<br>• Phenylacetylglutamine<br>• p-Cresol sulfate<br>• (Significant in urine, but not plasma; dark chcolate consumption further modulated metabolites associated with anxiety traits)	(10)
Cocoa	Chocolate consumption between ‘chocolate desiring’ vs. ‘chocolate	50 g	5-day, double crossover	22 healthy men	NMR	Urine	• **2-hydroxyhippurate**<br>• **2-Hydroxyisobutyrate**<br>• **4-cresol**<br>• **Acetone**<br>• **Carnitine**<br>• **Isobutyrate**	(11)

Summary of Untargeted Studies Presenting Candidate FIBs for Fermented Foods

Fermented Food	Intervention	Dose	Study Design	*n*	Analytical Method	Biosample	Candidate FIBs[a]	Reference
	indifferent' individuals						• **Methylsuccinate**<br>• **N-acetyl-carnitine**<br>• **Taurine**<br>• **Trigonelline**<br>• **Trimethylamine**<br>• 3-Hydroxyisobutyrate<br>• 4-Hydroxyphenylacetate<br>• Acetoacetate<br>• Citrate<br>• Dimethylglycine<br>• Glycine<br>• Phenylacetylglutamine	
Cocoa, coffee, wine	Chocolate candies	Habitual intake; dose not reported (from FFQ)	Nested case-control	1369 premenopausal women	U(H)PLC-MS/MS	Serum	• **3,7-Dimethylurate**<br>• **3-Methylxanthine**<br>• **7-Methylurate**<br>• **7-Methylxanthine**<br>• **Theobromine**	(12)
	Coffee, caffeinated	Habitual intake; dose not reported (from FFQ)					• **1,3,7-Trimethylurate**<br>• **1,3-Dimethylurate**<br>• **1,7-Dimethylurate**<br>• **1-Methylurate**<br>• **1-Methylxanthine**<br>• **AAMU**<br>• **Caffeine**<br>• **Paraxanthine**<br>• **Theophylline**	
	Coffee, decaffeinated	Habitual intake; dose not reported (from FFQ)					• **2,3-dihydroxypyridine**	
	Red wine	Habitual intake; dose not reported (from FFQ)					• **2,3-Dihydroxyisovalerate**<br>• **Ethyl glucuronide**	
	Total coffee	Habitual intake; dose not reported (from FFQ)					• **3-Hydroxypyridine sulfate**<br>• **3-Methyl catechol sulfate**<br>• **Citraconate/glutaconate**<br>• **Quinate**<br>• **Trigonelline**	
	Total wine	Habitual intake; dose not reported (from FFQ)					• **2,3-Dihydroxyisovalerate**<br>• **Androstenediol (3β,17β) monosulfate**<br>• **3-Carboxy-4-methyl-5-propyl-2-furanpropanoic acid**<br>• **Ethyl glucuronide**	

Summary of Untargeted Studies Presenting Candidate FIBs for Fermented Foods								
**Fermented Food**	**Intervention**	**Dose**	**Study Design**	***n***	**Analytical Method**	**Biosample**	**Candidate FIBs[a]**	**Reference**
							• **Oleoyl-linoleoyl-glycerol (18:1/18:2)**<br>• **Sphingomyelin (d18:2/18:1)**	
	White wine	Habitual intake; dose not reported (from FFQ)					• **2,3-Dihydroxyisovalerate**<br>• **Ethyl glucuronide**	
Bread	Wholegrain sourdough rye at different doses	0 g/day, 48 g/day, 96 g/day	4-week, randomized, controlled, crossover	33 healthy adults	FIE-MS	Urine	• **HBOA glucuronide**<br>• **HHPAA glucuronide**<br>• **HHPAA sulfate**<br>• **C13H21O3 glucuronide**<br>• **C14H25O4 glucuronide**<br>• **Creatinine**<br>• **HPAA sulfate**<br>• **N-feruloylglycine sulfate**<br>• **Phenylacetylglutamine derivative**	(13)
Bread	Sourdough fermented endosperm rye vs. white wheat bread	110.6 g (sourdough); 105.9 g (white wheat bread)	Acute, randomized, controlled, crossover	16 healthy adults	GCxGC-TOF-MS	Plasma	Sourdough:<br>• **2,4-Dihydroxybutanoic acid**<br>• **2-Oxo-butanoic acid**<br>• **Alpha-ketoglutaric acid**<br>• **Benzeneacetic acid**<br>• **Citrate**<br>• **Lysine**<br>• **Methionine**<br>• **Norvaline**<br>• **Phenylalanine**<br>• **Propanedioic acid**<br>• **Ribitol**<br>• **Threonic acid**<br>White wheat bread:<br>• **2-(Z)-Butenedioic acid**<br>• **2,4-Dihydroxybutanoic acid**<br>• **2,8-Dihydroxyquinoline glucuronide**<br>• **2-Oxo-butanoic acid**<br>• **Alpha-ketoglutaric acid**<br>• **Ascorbic acid**<br>• **Benzeneacetic acid**<br>• **Hydrocaffeic acid**<br>• **Lysine**<br>• **Norleucine**<br>• **Picolinic acid**<br>• **Propanedioic acid**<br>• **Succinic acid**	(14)
Bread	White bread vs. non-consumers	>1 portion/day (habitual intake)	Multi-centre, randomized,	255 healthy adults	HPLC-QTOF-MS	Urine	• **2-Aminophenol sulfate**<br>• **2-Hydroxy-7-methoxy-2H-1,4-benzoxazin-3-one**	(15)

Summary of Untargeted Studies Presenting Candidate FIBs for Fermented Foods								
**Fermented Food**	**Intervention**	**Dose**	**Study Design**	***n***	**Analytical Method**	**Biosample**	**Candidate FIBs[a]**	**Reference**
			controlled, parallel				• **2-Hydroxy-7-methoxy-2H-1,4-benzoxazin-3-one glucuronide**<br>• **DHPPA glucuronide**<br>• **Hydroxybenzoic acid glucuronide**<br>• **HPAA glucuronide**<br>• **Riboflavin**	
	Wholegrain bread vs. non-consumers	>1 portion/day (habitual intake)					• **2,8-Dihydroxyquinoline glucuronide**<br>• **2-Aminophenol sulfate**<br>• **HBOA glycoside**<br>• **2-Hydroxy-7-methoxy-2H-1,4-benzoxazin-3-one**<br>• **2-Hydroxy-7-methoxy-2H-1,4-benzoxazin-3-one glucuronide**<br>• **HHPAA**<br>• **3,5-Dihydroxyphenylethanol sulfate**<br>• **3-Indolecarboxylic acid glucuronide**<br>• **DHPPA glucuronide**<br>• **DHPPTA sulfate**<br>• **Dihydroferulic acid sulfate**<br>• **Enterolactone glucuronide**<br>• **Hydroxybenzoic acid glucuronide**<br>• **HPAA glucuronide**<br>• **Pyrraline**<br>• **Riboflavin**	
Bread	Whole grain bread replacement and high fish and bilberry consumption vs. wholegrain replacement and normal fish and berry consumption vs. avoidance of wholegrain cereals, fish, and berries	20-25% of total daily energy intake	12-week, randomized, controlled, parallel	106 healthy adults	U(H)PLC-QTOF-MS	Plasma	• AR 21:1-Gln<br>• AR 19:0-Gln<br>• Gamma-Butyrobetaine<br>Pipecolic acid betaine	(16)
Bread	Rye bread vs. wheat bread	214 g/day (rye); 179 g/day (wheat)	8 week, randomized, controlled, crossover	39 postmenopausal women	GCxGC-TOF-MS	Plasma	Rye bread:<br>• **Campesterol**<br>• **Dodecanamide**<br>• **Indole-3-acetic acid**<br>• **Inosose**<br>• **myo-Inositol**<br>• **Ribitol**	(17)

Summary of Untargeted Studies Presenting Candidate FIBs for Fermented Foods								
**Fermented Food**	**Intervention**	**Dose**	**Study Design**	***n***	**Analytical Method**	**Biosample**	**Candidate FIBs[a]**	**Reference**
							• **Ribonic acid**<br>• **Silanamine**<br>• **Sitosterol**<br>• **Trimethylsiloxy proline**<br>Wheat bread:<br>• **1-Monooleoylglycerol trimethylsilyl ether**<br>• **4-[39-(Triethylsilyl)propyl]phenol**<br>• **Butanoic acid**<br>• **d-Erythrotetrofuranose**<br>• **Glutamic acid**<br>• **N-Methyl-N-(2,4,6-trimethylphenyl)formamide**<br>• **Palmitic acid**<br>• **Pyrrole-2,5-dione trimethylsilate**<br>• **Tyrosine**<br>• **Urate**	
Bread	Wholegrain rye vs. white wheat bread enriched with fermented rye bran	6-10 slices/day	4-week, randomized, controlled, crossover	15 healthy adults	U(H)PLC-QTOF-MS	Plasma	• **AR C17:0-glucuronide**<br>• **AR C17:0-sulfate**<br>• **AR C19:0-glucuronide**<br>• **AR C19:0-sulfate**<br>• **AR C19:1-glucuronide**<br>• **AR C19:1-sulfate**<br>• **AR C21:0-glucuronide**<br>• **AR C21:1-glucuronide**<br>• **AR C21:1-sulfate**<br>• **AR C21:2-glucuronide**<br>• **AR C23:2-glucuronide**<br>• **3,5-Dihydrobenzoic acid**<br>• **2-Aminophenol sulfate**<br>**Pipecolic acid betaine**	(18)
Bread	Rye bran wheat bread vs. wholegrain wheat bread	196 g (rye grain); 208 g (wholegrain)	Acute crossover	12 healthy volunteers	UPLC-MS/MS (untargeted)	Urine	• Cysteine<br>• N-acetylcysteine<br>• Indolelactate<br>• 4-Acetamidobutanoate<br>• Imidazole lactate<br>• trans-4-Hydroxyproline<br>• N-Acetylarginine<br>• N2,N5-Diacetylornithine<br>• Argininosuccinate<br>• Creatinine<br>• 5-Hydroxyindoleacetate<br>• Xanthurenate<br>• Dopamine sulfate<br>• N-Acetylputrescine<br>• Cysteinylglycine	(19)

Summary of Untargeted Studies Presenting Candidate FIBs for Fermented Foods								
**Fermented Food**	**Intervention**	**Dose**	**Study Design**	***n***	**Analytical Method**	**Biosample**	**Candidate FIBs[a]**	**Reference**
							• Phenylacetylglutamine<br>• Citramalate<br>• Pseudouridine<br>• 4-Ureidobutyrate<br>• Uridine<br>• 5,6-Dihydrouracil<br>• Thymine<br>• 3-Methylcytidine<br>• 7-Methylguanine<br>• N2,N2-Dimethylguanosine<br>• N1-Methyladenosine<br>• N6-Carbamoylthreonyladenosine<br>• Adenosine<br>• Adenine<br>• Urate<br>• Azelate<br>• Pimelate<br>• Dimethylmalonic acid<br>• 2-Aminooctanoate<br>• Ribitol<br>• Ribulose/xylulose<br>• N1-Methyl-2-pyridone-5-carboxamide<br>• 1-Methylnicotinamide<br>• Oxalate<br>• Citraconate/glutaconate<br>• **Tartarate**<br>• **Vanillic acid**<br>• **3,5-DHBA**<br>• **Ferulic acid 4-sulfate**<br>• **Syringic acid**<br>• **2,3-Dihydroxyisovalerate**<br>• **2-Oxindole-3-acetate**<br>• **Gentisic acid-5-glucoside**<br>• **4-Vinylguaiacol sulfate**<br>• **1,2,3-Benzenetriol sulfate**<br>• **3-Hydroxypyridine sulfate**<br>• **1,2,3-Benzenetriol sulfate**<br>• Lanthionine<br>• Sulfate<br>• HPAA sulfate<br>• 4-Acetylphenol sulfate<br>• 3-Methylcatechol sulfate<br>• 3-Methylcatechol sulfate<br>• 3-Methoxycatechol sulfate	

Summary of Untargeted Studies Presenting Candidate FIBs for Fermented Foods								
**Fermented Food**	**Intervention**	**Dose**	**Study Design**	***n***	**Analytical Method**	**Biosample**	**Candidate FIBs[a]**	**Reference**
							• 1-Methylxanthine<br>• 1-Methylurate	
					HPLC-MS/MS (Targeted)		• 3,5-DHBA<br>• 3,5-DHPPTA<br>• 3,5-DHBA glycine<br>• 3,5-DHBA sulfate<br>• 3,5-DHPPA sulfate<br>• 3,5-DHPPTA sulfate<br>• 3,5-DHPHTA sulfate<br>• 2-(3,5-dihydroxyphenyl)ethanol sulfate<br>• HPAA<br>• HPAA sulfate<br>• 2-Aminophenol sulfate<br>• HHPAA sulfate<br>• HHPPA sulfate<br>• Isopropyl 2hydroxyphenylcarbamate<br>• Ferulic acid sulfate<br>• Caffeic acid sulfate<br>• Vanillic acid sulfate<br>• Homovanillic acid sulfate<br>• DHFA sulfate<br>• Feruloyglycine<br>• Feruloyglycine sulfate<br>• Glycochenodeoxycholic acid<br>• Glycochenodeoxychol-5en-24-oic acid<br>• Glycochenodeoxycholic acid glucuronide<br>• Glycochenodeoxychol-5en-24-oic acid glucuronide<br>• Enterolactone glucuronide	
Bread	Wholegrain sourdough bread vs. white wheat bread enriched with native unprocessed rye bran vs. white wheat bread enriched with bioprocessed rye bran vs. white wheat bread control	109 to 166 g (standardised to 50 g carbohydrates and 20 g fiber)	Acute, randomized, crossover	12 healthy volunteers	LC-QTOF-MS and MS/MS	Plasma	• Benzoxazinoid-derived phenylacetamide sulfates (hydroxy-N-(2-hydroxyphenyl) acetamine, and N-(2-hydroxyphenyl) acetamide)<br>(Fermentation has a central role in modulating the phytochemical profile of the breads)	(20)

Summary of Untargeted Studies Presenting Candidate FIBs for Fermented Foods								
**Fermented Food**	**Intervention**	**Dose**	**Study Design**	***n***	**Analytical Method**	**Biosample**	**Candidate FIBs[a]**	**Reference**
Bread	Wholegrain bread vs. wholemeal rye bread	Advised to take test products instead of customarily used breads and baked products	4-week, randomized, crossover	20 volunteers with slightly elevated serum cholesterol	UPLC-QTOF-MS	Urine	• 3,5-Dihydroxyhydrocinamic acid sulfate<br>• Non-identified nitrogen containing metabolite<br>• Ascorbic acid<br>• Non-identified nitrogen containing metabolite<br>• 2-Aminophenol sulfate<br>• Non-identified nitrogen containing metabolite<br>• Nonanedioic acid<br>• DHPPA glucuronide<br>• Indolylacryloylglycine<br>• Enterolactone glucuronide<br>• DHPPA sulfate<br>• Non-identified nitrogen containing metabolite<br>• Ferulic acid-4-O-sulfate<br>• 2,4-Dihydroxy-1,4-benzoxazin3-one sulfate<br>• 3,5-Dihydroxyphenylethanol sulfate<br>• 1,3,4,5Tetrahydroxycyclohexane-1carboxylic acid	(21)
Coffee	Coffee, lower intake vs. Coffee, higher intake	4 cups/day (600 mL/day) lower intake; 8 cups/day (1200mL/day) higher intake	1-month, single-blind, crossover	47 healthy adults	U(H)PLC-ESI-MS/MS	Serum	Lower intake:<br>• **Creatinine**<br>• **Guanidinoacetate**<br>• **Imidazole lactate**<br>• **Isovalerylcarnitine**<br>• **Cysteine**<br>• **4-Acetamidobutanoate**<br>• **N-acetylputrescine**<br>• **Indole-3-lactic acid**<br>• **Kynurenine**<br>• **2-Hydroxyphenylacetate**<br>• **Glucuronate**<br>• **Trigonelline**<br>• **Citraconate/glutaconate**<br>• **4-Androsten-3beta,17beta-diol monosulfate**<br>• **Epiandrosterone sulfate**<br>• **Etiocholanolone glucuronide**<br>• **Urate**<br>• **7-Methylguanine**<br>• **N-acetylcarnosine**<br>• **DSGEGDFXAEGGGVR***<br>• **3-(3-hydroxyphenyl) propionate**<br>• **3-(3-hydroxyphenyl) propionate sulfate**<br>• **3-Hydroxyhippurate**<br>• **3-Methyl catechol sulfate**<br>• **3-Phenylpropionate**<br>• **4-Vinylphenol sulfate**<br>• **Catechol sulfate**	(22)

Summary of Untargeted Studies Presenting Candidate FIBs for Fermented Foods								
**Fermented Food**	**Intervention**	**Dose**	**Study Design**	***n***	**Analytical Method**	**Biosample**	**Candidate FIBs[a]**	**Reference**
							• **Hippurate**<br>• **O-methylcatechol sulfate**<br>• **3-Hydroxypyridine sulfate**<br>• **N-methylpipecolate**<br>• **Cinnamoylglycine**<br>• **Dihydroferulic acid**<br>• **Homostachydrine**<br>• **N-(2-furoyl)glycine**<br>• **Pyrraline**<br>• **Quinate**<br>• **1,3,7-Trimethylurate**<br>• **1,3-Dimethylurate**<br>• **1,7-Dimethylurate**<br>• **1-Methylurate**<br>• **1-Methylxanthine**<br>• **3,7-Dimethylurate**<br>• **3-Methylxanthine**<br>• **AAMU**<br>• **7-Methylxanthine**<br>• **Caffeic acid sulfate**<br>• **Caffeine**<br>• **Paraxanthine**<br>• **Theobromine**<br>• **Theophylline**<br>Higher intake:<br>• **Creatinine**<br>• **Guanidinoacetate**<br>• **Imidazole lactate**<br>• **Isovalerylcarnitine**<br>• **Cysteine**<br>• **4-Acetamidobutanoate**<br>• **N-acetylputrescine**<br>• **Indole-3-lactic acid**<br>• **Kynurenine**<br>• **2-Hydroxyphenylacetate**<br>• **Glucuronate**<br>• **Trigonelline**<br>• **Citraconate/glutaconate**<br>• **4-Androsten-3beta,17beta-diol monosulfate**<br>• **Epiandrosterone sulfate**<br>• **Etiocholanolone glucuronide**<br>• **Urate**<br>• **7-Methylguanine**<br>• **N-acetylcarnosine**	

Summary of Untargeted Studies Presenting Candidate FIBs for Fermented Foods								
**Fermented Food**	**Intervention**	**Dose**	**Study Design**	***n***	**Analytical Method**	**Biosample**	**Candidate FIBs[a]**	**Reference**
							• **DSGEGDFXAEGGGVR**<br>• **3-(3-hydroxyphenyl) propionate**<br>• **3-(3-hydroxyphenyl) propionate sulfate**<br>• **3-Hydroxyhippurate**<br>• **3-Methyl catechol sulfate**<br>• **3-Phenylpropionate**<br>• **4-Vinylphenol sulfate**<br>• **Catechol sulfate**<br>• **Hippurate**<br>• **O-methylcatechol sulfate**<br>• **3-Hydroxypyridine sulfate**<br>• **N-methylpipecolate**<br>• **Cinnamoylglycine**<br>• **Dihydroferulic acid**<br>• **Homostachydrine**<br>• **N-(2-furoyl)glycine**<br>• **Pyrraline**<br>• **Quinate**<br>• **1,3,7-Trimethylurate**<br>• **1,3-Dimethylurate**<br>• **1,7-Dimethylurate**<br>• **1-Methylurate**<br>• **1-Methylxanthine**<br>• **3,7-Dimethylurate**<br>• **3-Methylxanthine**<br>• **AAMU**<br>• **7-Methylxanthine**<br>• **Caffeic acid sulfate**<br>• **Caffeine**<br>• **Paraxanthine**<br>• **Theobromine**<br>• **Theophylline**<br>• **Campesterol**<br>• **N6-carbamoylthreonyladenosine**<br>• **Palmitoyl dihydrosphingomyelin**<br>• **Phosphate**	
Coffee	Coffee, high consumption vs. coffee low consumption	1546 g/day (high); 337 g/day (low) (from FFQ)	Nested case-control	489 healthy adults	LC-MS and GC-MS	Serum	• **1,3,7-Trimethylurate**<br>• **1,7-Dimethylurate**<br>• **1-Methylurate**<br>• **1-Methylxanthine**<br>• **3-(3-hydroxyphenyl) propionate**<br>• **3-Hydroxyhippurate**<br>• **4-Vinylphenol sulfate**<br>• **Caffeine**	(23)

Summary of Untargeted Studies Presenting Candidate FIBs for Fermented Foods								
**Fermented Food**	**Intervention**	**Dose**	**Study Design**	***n***	**Analytical Method**	**Biosample**	**Candidate FIBs[a]**	**Reference**
							• **Catechol sulfate**<br>• **Cinnamoylglycine**<br>• **Cyclo(leu-pro)**<br>• **N-(2-furoyl)glycine**<br>• **Paraxanthine**<br>• **Quinate**<br>• **Theobromine**<br>• **Theophylline**<br>• **Trigonelline**	
Coffee	Coffee	200 mL	Acute intervention	8 healthy adults	NMR	Urine	• **2-Furoylglycine**<br>• **N-methylpyridinium**	(24)
Coffee	Coffee	350 mL	Acute intervention	9 healthy adults	HILIC-U(H)PLC-TOF-MS	Urine	• **N-methylpyridinium**<br>• **Trigonelline**<br>• **1,3- or 1,7-dimethyluric acid**<br>• **3-Methylxanthine**<br>• **7-Methylxanthine**<br>• **Caffeine**<br>• **Catechol sulfate**<br>• **Dihydrocaffeic acid sulfate**<br>• **Dihydroferulic acid**<br>• **Dihydroferulic acid glucuronide**<br>• **Dihydroferulic acid sulfate**<br>• **Ferulic acid**<br>• **Ferulic acid glucuronide**<br>• **Ferulic acid sulfate**<br>• **Guaiacol sulfate**<br>• **N-feruloylglycine**<br>• **Paraxanthine**<br>• **Theobromine**<br>• **Theophylline**<br>• **Trigonelline**	(25)
Coffee	Coffee, instant	400 mL	Acute, randomized, controlled, crossover	9 healthy adults	U(H)PLC-MS	Plasma	• 4-Feruloylquinic acid<br>• 4-Feruloylquinic acid lactone<br>• Caffeic acid 3-O-sulfate<br>• Caffeic acid 4-O-sulfate<br>• Caffeoylquinic acid lactone O-sulfates<br>• Dihydrocaffeic acid<br>• Dihydrocaffeic acid 3-O-glucuronide<br>• Dihydrocaffeic acid 3-O-sulfate<br>• Dihydrocaffeic acid 4-O-sulfate<br>• Dihydrodihydrodimethoxycinnamic acid<br>• Dihydroferulic acid<br>• Dihydroferulic acid 4-O-sulfate<br>• Dihydroisoferulic acid	(26)

Summary of Untargeted Studies Presenting Candidate FIBs for Fermented Foods								
**Fermented Food**	**Intervention**	**Dose**	**Study Design**	***n***	**Analytical Method**	**Biosample**	**Candidate FIBs[a]**	**Reference**
							• Dihydroisoferulic acid 3-O-sulfate<br>• Dihydroisoferulic acid O-glucuronide<br>• Dimethoxycinnamic acid<br>• Ferulic acid<br>• (iso)Ferulic acid<br>• (iso)Ferulic acid 3-O-glucuronide<br>• (iso)Ferulic acid 3-O-sulfate<br>• Ferulic acid 4-O-glucuronide<br>• Ferulic acid 4-O-sulfate<br>• 3-Feruloylquinic acid<br>• 5-Feruloylquinic acid<br>• 3-Feruloylquinic acid lactone<br>• Feruloylquinic acid lactone O-glucuronide<br>• Feruloylquinic acid lactone O-sulfate<br>• m-CoA O-sulfate<br>• o-CoA O-sulfate<br>p-DHCoAO-sulfate	
Coffee	High coffee consumption vs. low coffee consumption	183 to 540 mL/day (high); 0 mL/day (low) (from FFQ, multiple 24h recalls)	Prospective cohort	39 healthy adults (20 high consumers; 19 low consumers)	U(H)PLC-QTOF-MS	Urine	• **1,3- or 3,7-Dimethyluric acid**<br>• **1,7-Dimethyluric acid**<br>• **1-Methyluric acid**<br>• **1-Methylxanthine**<br>• **3-Hydroxyhippuric acid**<br>• **AFMU**<br>• **Atractyligenin glucuronide**<br>• **Caffeine**<br>• **Cyclo(isoleucyl-prolyl)**<br>• **Dimethylxanthine (paraxanthine or theophylline) glucuronide**<br>• **Hippurate**<br>• **Kahweol oxide glucuronide**<br>• **Kahweol oxide glucuronide analogue**<br>• **Paraxanthine**<br>• **Trigonelline**<br>• **Trimethyluric acid**	(27)
Coffee	Coffee	4 cups/day; 8 cups/day	1-month controlled, crossover	47 healthy adults, habitual coffee drinkers	IMS-MS (lipidomics)	Serum	Low-dose:<br>• TAG47:1-FA17:0<br>• TAG52:5-FA20:5<br>• PC(18:0/16:1)<br>• PC(18:0/18:3)<br>• PC(18:0/20:2)<br>• PE(O-16:0/18:2)<br>• PE(P-16:0/18:2)<br>• PE(P-18:0/18:2)<br>• DCER(24:0)	(28)

Summary of Untargeted Studies Presenting Candidate FIBs for Fermented Foods								
**Fermented Food**	**Intervention**	**Dose**	**Study Design**	***n***	**Analytical Method**	**Biosample**	**Candidate FIBs[a]**	**Reference**
							High-dose:<br>• **TAG47:1-FA17:0**<br>• **TAG52:5-FA20:5**<br>• **TAG60:11-FA22:5**<br>• **PC(18:0/16:1)**<br>• **PC(18:0/18:3)**<br>• **PC(18:0/20:2)**<br>• **PC(18:0/20:3)**<br>• **PE(18:0/20:1)**<br>• **PE(O-16:0/18:2)**<br>• **PE(O-18:0/20:3)**<br>• **PE(P-16:0/18:2)**<br>• **PE(P-18:0/18:2)**<br>• **DCER(24:0)**<br>• **LCER(26:1)**	
Coffee	Coffee consumers vs. non-consumers	Consumers (mean 506.4 g/day for women, 526.4 g/day for men); Non-consumers (0 g/day)	Cross-sectional, KarMeN study	48 healthy adults (consumers); 49 healthy adults (non-consumers)	HS-SPME-GCxGC-MS	Urine	• **3,4-Dimethyl-2,5-furadione**<br>• **2-Methyl-furan**<br>• **Guaicol**<br>• **2-Methyl-butanoic acid**<br>• **3-Methyl-butanoic acid**<br>• **2-Vinylfuran**	(29)
Coffee	Total coffee	Consumers (≥50 mL/day)	Cross-sectional, PREDIMED study	1379 adults with T2D or CV risk factors; 285 non-coffee consumers	LC-MS/MS, U(H)PLC-Exactive Plus orbitrap MS	Plasma	• **Sphingomyelin (C24:0)**<br>• **Caffeine**<br>• **AAMU**<br>• **Cotinine**	(30)
	Caffeinated coffee	Consumers (≥50 mL/day)		512 adults with T2D or CV risk factors			• **Sphingomyelin (C24:0)**<br>• **Caffeine**<br>• **AAMU**<br>• **Cotinine**	
	Decaffeinated coffee	Consumers (≥50 mL/day)		721 adults with T2D or CV risk factors			• **Alpha-glycerophosphate**<br>• **Hydroxyhippurate**<br>• **Hippurate**<br>• **Sphingomyelin (C24:0)**<br>• **Phosphatidylcholine (C40:6)**	
Coffee	Habitual coffee intake	253 mL/day (France); 437 mL/day (Germany); 154 mL/day (Greece); 99 mL/day (Italy)	EPIC study (France, Germany, Italy, Greece)	451 participants from the EPIC cohort	U(H)PLC-MS/MS	Serum	• **Trigonelline**<br>• **Caffeine**<br>• **Paraxanthine**<br>• **AAMU**<br>• **Quinic acid**<br>• **Cyclo(prolyl)-valyl**<br>• **Cyclo(isoleucyl)-prolyl**<br>• **Cyclo(leucyl)-prolyl**	(31)

Summary of Untargeted Studies Presenting Candidate FIBs for Fermented Foods								
**Fermented Food**	**Intervention**	**Dose**	**Study Design**	***n***	**Analytical Method**	**Biosample**	**Candidate FIBs[a]**	**Reference**
							• **Pyrocatechol sulfate**	
Coffee, multiple foods	Habitual intake of 58 different foods based on FFQ	<1X/week, 1X/week, 2-4X/week, 5-6X/week, 1X/day, >1X/day	Prospective cohort	68 volunteers from the GrainMark cohort (FFQ)	FIE-MS	Urine	• Dihydrocaffeic acid, hippuric acid, caffeic acid for coffee intake<br>• Metabolites from other foods, including cheese, chocolate, wine, beer, and other alcoholic beverages were not reported	(32)
Coffee	Habitual coffee consumption	Frequency of coffee consumption and number of 250 mL cups each consumption (from FFQ)	Association study	564 healthy volunteers from the Hong Kong Osteoporosis Study	LC-MS (Untargeted)	Serum	• **Quinate**<br>• **3-Hydroxypyridine sulfate**<br>• **Trigonelline**<br>• **AFMU**<br>• **AAMU**<br>• **1-Methylxanthine**<br>• **Paraxanthine**<br>• **3-Methyl catechol sulfate**<br>• **1-Methylurate**<br>• **1,7-Dimethylurate**<br>• **3-Hydroxyhippurate**	(33)
Coffee	Coffee intake	Frequency of coffee consumption (from FFQ)	Association study	1595 women from the Nurses Health Study I and II	LC-MS/MS (Untargeted)	Plasma	• **Trigonelline**<br>• **AAMU**<br>• **Cinnamoylglycine**<br>• **1,7-Dimethyluric acid**<br>• **Caffeine**<br>• **Phenyllactic acid**<br>• **4-Hydroxyhippuric acid**<br>• **Cytosine**<br>• **7-Methylxanthine**<br>• **L-carnitine**<br>• **C20:4 cholesterol ester**<br>• **C18:1 cholesterol ester**<br>• **C18:2 cholesterol ester**	(34)
Coffee	Habitual coffee intake, boiled and filtered coffee intake	Frequency of coffee intale (including boiled and filtered) (from FFQ)	Nested case-control	421 case-control pairs and 129 at 10-year follow-up	LC-MS (Untargeted)	Plasma	Filtered coffee:<br>• **1-Methyluric acid**<br>• **Quinic acid**<br>• **Theobromine**<br>• **2-Furoylglycine**<br>• **AAMU**<br>• **Cyclo(leucyl-prolyl)**<br>• **7-hydroxy-4(methoxymethyl)coumarin**<br>Boiled coffee:<br>• **LysoPE(20:4)**<br>• **PE(20:4/16:0)**<br>General:<br>• **Caffeine**	(35)

Summary of Untargeted Studies Presenting Candidate FIBs for Fermented Foods								
**Fermented Food**	**Intervention**	**Dose**	**Study Design**	***n***	**Analytical Method**	**Biosample**	**Candidate FIBs[a]**	**Reference**
							• **Paraxanthine**<br>• **Theophylline**<br>• **Trigonelline**<br>• **Atractyligenin glucuronide**<br>• **Ethyl 3-mercaptopropanoic acid**<br>• **LysoPC(24:0)**<br>• **LysoPE(22:4)**<br>• **LysoPE(22:5)**<br>• **LysoPE(24:0)**<br>• **N-Methylpyridinium**<br>• **Sinapic acid**<br>• **Dihydroferulic acid 4-sulphate**	
Wine	Red wine vs. no red wine	250 mL/day	4-week, randomized, controlled, parallel	33 healthy adults; 8 in control group	U(H)PLC-TOF-MS	Urine	• **(3S,5R,6S,7E,9x)-7-megastigmene-3,6,9-triol 9-glucoside**<br>• **(E)-2-propenyl [3-(2-propenylthio)-2-propenyl] sulfate**<br>• **1-(2,3-dihydro-1H-pyrrolizin-5-yl)-2-propen-1-one**<br>• **2,3-Dihydroxy-3-methylvaleric acid**<br>• **2,3-Dihydroxyvaleric acid**<br>• **2,3-Dihydroxyvaleric acid**<br>• **2,3-Dimethyl-3-hydroxyglutaric acid**<br>• **2-Isopropyl-3-oxosuccinate**<br>• **3-Carboxy-4-methyl-5-pentyl-2-furanpropanoic acid**<br>• **3-Methoxy-4-hydroxyphenylglycol sulfate**<br>• **3-Methylcrotonylglycine**<br>• **4′,6′-Dihydroxy-2′-methoxyacetophenone 6′-glucoside**<br>• **4-Chloro-3-[(2-chloro-5-nitrobenzoyl)carbamothioylamino]benzoic acid**<br>• **4-Hydroxy-5-(dihydroxyphenyl) valeric acid-O-sulfate**<br>• **4-Hydroxy-5-(phenyl) valeric acid-O-glucuronide**<br>• **4-Hydroxy-5-(phenyl) valeric acid-O-sulfate**<br>• **5-(1-propynyl)-5-vinyl-2,2-bithiophene**<br>• **DHPV sulfate**<br>• **5,7-dihydroxy-3′,4′-dimethoxy-5′-prenylflavanone**<br>• **5-Methoxybilobetin**<br>• **AFMU**<br>• **Aurantricholide B**	(36)

Summary of Untargeted Studies Presenting Candidate FIBs for Fermented Foods								
Fermented Food	Intervention	Dose	Study Design	*n*	Analytical Method	Biosample	Candidate FIBs[a]	Reference
							• Azaspirazid<br>• Caffeic acid<br>• Catechol sulfate (pyrocatechol sulfate)<br>• Coumaroyl−glucose<br>• DHPV-O-methyl-O-sulfate<br>• DHPV-O-glucuronide<br>• Dibenzyl disulfide<br>• Dihydropteridine<br>• Ethyl 1-(ethylthio)propyl disulfide<br>• Ethyl maltol<br>• (iso)Ferulic acid sulfate<br>• (iso)Ferulic acid sulfate<br>• Galactosylglycerol<br>• Glucosinalbin<br>• Hesperetin-O-sulfate<br>• Hordatine B glucoside<br>• Hydroxytyrosol<br>• Kanzonol I<br>• Kanzonol R<br>• Luteolin sulfate<br>• L-γ-glutamyl-L-(iso)leucine<br>• Methyl helianthenoate F glucoside<br>• Methylisocitric acid<br>• Monoglyceride citrate<br>• O-methoxycatechol-O-sulfate<br>• O-methoxycatechol-O-sulfate<br>• O-ureidohomoserine<br>• Oxovaleric acid<br>• p-Chlorobenzenesulfonyl urea<br>• Phenol sulfate<br>• Phenylalanyl aspartate<br>• Phloroglucinol<br>• Pyrogallol sulfate<br>• Salicylate glucuronide<br>• Sulfohydroxybenzoic acid<br>• Tartaric acid<br>• Tyrosol sulfate<br>• Tyrosol sulfate<br>• Valechlorin<br>• Vanillic acid 4-sulfate<br>• Vanillin 4-sulfate<br>• Wthyl hydrogen sulfate<br>• Wyeronic acid<br>• α-Terpinyl cinnamate	

Summary of Untargeted Studies Presenting Candidate FIBs for Fermented Foods

Fermented Food	Intervention	Dose	Study Design	*n*	Analytical Method	Biosample	Candidate FIBs[a]	Reference
Wine	Wine (administered with grapes) administered as part of a food challenge	Not reported	Acute intervention	7 healthy adults	NMR	Urine	• **2,3-Butanediol**<br>• **2-Isopropylmalate**<br>• **Diethylmalonate**	(37)
Wine	Red wine vs. no red wine	250 mL/day	4-week, randomized, controlled, parallel	33 healthy adults; 8 in control group	U(H)PLC-TOF (untargeted)	Feces	• **2,3-Pentanedione acid**<br>• **2-Hydroxyglutaric acid**<br>• **2-Methylbutyric acid**<br>• **2-Phenethyl butyrate**<br>• **2-Phenylethyl hexanoate**<br>• **4-Hydroxy-5-(3-hydroxyphenyl) valeric acid**<br>• **4-Hydroxy-5-(phenyl) valeric acid**<br>• **Benzoic acid**<br>• **Cholesterol sulfate**<br>• **Deoxycholic acid**<br>• **DHPV**<br>• **Diethylmalonate**<br>• **Docosahexaenoic acid methyl ester**<br>• **Glutaric acid**<br>• **Stercobilin**<br>• **Urobilinogen**	(38)
					U(H)PLC-ESI-MS/MS (targeted for microbial phenolics)		• **DHBA**<br>• **3-Hydroxyphenylacetic acid**<br>• **3-O-Methylgallic acid**<br>• **3-Phenylpropionate**<br>• **4-hydroxy-5-(3,4-dihydroxyphenyl) valeric acid**<br>• **4-Hydroxy-5-(phenyl) valeric acid**<br>• **DHPV**<br>• **Protocatechuic acid**<br>• **Syringic acid**<br>• **Vanillic acid**	
Wine	Red wine vs. no red wine	250 mL/day	4-week, randomized, controlled, parallel	33 healthy adults; 8 in control group	U(H)PLC-TOF-MS	Feces	• **2,3,-Pentanedione**<br>• **2-Hydroxyglutaric acid**<br>• **2-Methylbutyric acid**<br>• **2-Phenethyl butyrate**<br>• **2-Phenylethyl hexanoate**<br>• **4-Hydroxy-5-(3-hydroxyphenyl) valeric acid**<br>• **4-Hydroxy-5-(phenyl) valeric acid**<br>• **Benzoic acid**<br>• **Cholesterol sulfate**<br>• **Deoxycholic acid**<br>• **DHPV**<br>• **Diethylmalonate**	(39)

Summary of Untargeted Studies Presenting Candidate FIBs for Fermented Foods								
**Fermented Food**	**Intervention**	**Dose**	**Study Design**	***n***	**Analytical Method**	**Biosample**	**Candidate FIBs[a]**	**Reference**
							• **Docosahexaenoic acid methyl ester**	
Wine	Red wine, dealcoholized vs. red wine, alcoholized vs. gin (comparator)	272 mL/day (wines); 100 mL/day (gin)	28-day, randomized, controlled, crossover	61 healthy men with high CV risk	NMR	Urine	• **3-Methyl-2-oxovalerate**<br>• **4-Hydroxyphenylacetate**<br>• **Ethanol**<br>• **Hippurate**<br>• **Mannitol**<br>• **Tartrate**<br>• **Trigonelline**<br>• 1-Methylnicotinamide<br>• 2-Hydroxyisobutyrate<br>• 3-Hydroxyisobutyrate<br>• 3-Hydroxymandelate<br>• Acetate<br>• Acetoacetate<br>• Acetone<br>• Acetylcarnitine<br>• Alanine<br>• Betaine<br>• Carnitine<br>• cis-Aconitate<br>• Citrate<br>• Creatine<br>• Creatinine<br>• Dimethylamine<br>• Formate<br>• Fucose<br>• Glucose<br>• Glycine<br>• Glycylproline<br>• Histidine<br>• Indole-3-acetate<br>• Lactate<br>• Leucine<br>• Lysine<br>• Malonate<br>• Methylsuccinate<br>• N-Methylhistidine<br>• N-N<br>• N-Phenylacetylglycine<br>• Succinate<br>• Taurine<br>• Threonine<br>• Trimethylamine<br>• Trimethylamine-N-oxide	(40)

Summary of Untargeted Studies Presenting Candidate FIBs for Fermented Foods								
**Fermented Food**	**Intervention**	**Dose**	**Study Design**	***n***	**Analytical Method**	**Biosample**	**Candidate FIBs[a]**	**Reference**
							• Tyrosine<br>• Urea<br>• Valine	
Wine	Red wine, dealcoholized; Red wine, alcoholized; Gin (comparator)	272 mL/day (wines); 100 mL/day (gin)	28-day, randomized, controlled, crossover	56 adults/elderly with high CV risk	NMR	Urine	• **2,3-Butanediol**<br>• **2-methyl-2-oxovalerate**<br>• **4-Hydroxyphenylacetate**<br>• **Ethanol**<br>• **Ethyl glucuronide**<br>• **Hippurate**<br>• **Mannitol**<br>• **Tartrate**	(41)
	Wine	0 mL/day (non-consumer); <180 mL/day (intermittent); ≥180 mL/day (consumer)	5-year, multi-centre, randomized, controlled, parallel, single-blind (baseline assessment)	91 adults/elderly with high CV risk			• **2,3-Butanediol**<br>• **2-methyl-2-oxovalerate**<br>• **Ethanol**<br>• **Ethyl glucuronide**<br>• **Mannitol**<br>• **Tartrate**	
Wine	Red wine, dealcoholized vs. no wine	272 mL/day	28-day, randomized, controlled, crossover	57 adults/elderly with high CV risk	NMR	Urine	• **3-Hydroxyphenylacetic acid**<br>• **4-Hydroxyphenylacetate**<br>• **Betaine**<br>• **Dimethylamine**<br>• **Fucose**<br>• **Glucose**<br>• **Lactate**<br>• **Mannitol**<br>• **Methanol**<br>• **Tartrate**<br>• **Threonine**	(42)
Cheese	Cheese (hard, yellow Samsø)	143 g/day	6-week, randomized, controlled, crossover	23 healthy adults	U(H)PLC-QTOF-MS	Urine	• 4-Hydroxyphenylacetate<br>• Indoxyl sulfate<br>• Isobutyrylglycine<br>• Isovalerylglutamic acid<br>• Isovalerylglycine<br>• Triglylglycine<br>• Tyramine sulfate<br>• Xanthurenic acid	(43)
Cheese	Cheese (Gruyere)	100 g + 500 mL water	Acute, randomized, controlled, crossover	11 healthy adults	GC-MS	Urine	• **3-Phenyllactic acid**<br>• **4-Methylcatechol**<br>• **Alanine**<br>• **Lactic acid**<br>• **Pyroglutamic acid**	(44)
					NMR		• **Alanine**	

Summary of Untargeted Studies Presenting Candidate FIBs for Fermented Foods								
**Fermented Food**	**Intervention**	**Dose**	**Study Design**	***n***	**Analytical Method**	**Biosample**	**Candidate FIBs[a]**	**Reference**
							• **Proline**<br>• **Pyroglutamic acid**	
Cheese	Cheese (semihard, cow)	1.859 g/day	2-week, randomized, controlled, crossover	15 healthy men	NMR	Feces	• **Acetate**<br>• **Butyrate**<br>• **Malonate**<br>• **Propionate**	(45)
						Urine	• **Hippurate**<br>• **Proline betaine**<br>• **Tyrosine**<br>• **Urea**	
Cheese	Cheese vs. milk vs. soy beverage	100 g (cheese), 600 mL (milk or soy beverage)	Acute, randomized, controlled, crossover	11 healthy volunteers	HS-GC-MS (Untargeted)	Plasma, urine (volatile)	• **Heptan-2-one (plasma)**<br>• **Undecan-2-one (plasma)**<br>• **Heptan-4-one (urine)**	(46)
Cheese	Cheese vs. milk vs. soy beverage	100 g (cheese); 600 mL (milk or soy beverage)	Acute, randomized, controlled, crossover	11 healthy volunteers	LC-MS (Untargeted)	Plasma, urine	• **Aminoadipic acid**<br>• **Citrulline**<br>• **Valyl-threonine**<br>• **Phenylalanyl-proline**<br>• **Indolelactic acid**<br>• **Proline**	(47)
Cheese	Cheese (Gruyere) vs. baseline	100 g + 500 mL water	Acute, randomized, controlled, crossover	11 healthy adults	GC-MS	Serum	• **Methionine**<br>• **Proline**<br>• **Leucine**<br>• **Glutamic acid**<br>• **3-Phenyllactic acid**<br>• **Pentadecanoic acid (C15:0)**<br>• **Heptadecanoic acid (C17:0)**<br>• Lactose<br>• Galacitol<br>• Galactono-1,5-lactone<br>• Dodecanoic acid<br>• Linoleic acid<br>• Gamma-tocopherol<br>• Maltol<br>• Sucrose<br>• Guaiacol<br>• Catechol	(48)
					NMR		• **Methionine**<br>• **Tyrosine**<br>• **Valine+isoleucine**<br>• **3-Hydroxyisobutyrate**<br>• Lipid=CH-CH2-CH=	
Fermented milk	Probiotic fermented milk	0.4 L/day	8-week, randomized,	31 IBS patients	NMR	Serum	• **3-Hydroxybutyrate**<br>• **D-Lactate**	(49)

Summary of Untargeted Studies Presenting Candidate FIBs for Fermented Foods								
**Fermented Food**	**Intervention**	**Dose**	**Study Design**	***n***	**Analytical Method**	**Biosample**	**Candidate FIBs[a]**	**Reference**
			controlled, parallel				• **L-lactate**	
Fermented milk	Probiotic fermented milk	0.4 L/day	8-week, randomized, controlled, parallel	31 IBS patients	GC-MS	Serum	• **Aspartic acid**<br>• **Creatine/creatinine**<br>• **Glutamine**<br>• **Lactate**<br>• **Proline**	(50)
Yoghurt	Yoghurt vs. baseline	800 g	Acute, randomized, controlled, double-blind, crossover	14 healthy men	LC-MS	Serum	• **Phenylalanine**<br>• **Threonine**<br>• **Lysine**<br>• **Proline**<br>• **Asparagine**<br>• **Tyrosine**<br>• **Tryptophan**<br>• **Citrulline**<br>• **Indole-3-lactic acid**<br>• **Indole-3-acetaldehyde**	(51)
			2-week, randomized, controlled, double-blind, crossover				• **Proline**<br>• **Lysine**<br>• **Threonine**<br>• **Citrulline**<br>• **Indole-3-lactic acid**<br>• **Indole-3-acetaldehyde**	
Beer, coffee, wine	Beer	Habitual intake (dose not reported); assessed by FFQ	Case-control	125 patients with colon adenoma; 128 controls	U(H)PLC-MS/MS and GC-MS	Urine	• **Glycerol 3-phosphate**<br>• **Homovanillate sulfate**	(52)
	Coffee, caffeinated	Habitual intake (dose not reported); assessed by FFQ				Serum	• **1,3,7-Trimethylurate**<br>• **1,3-Dimethylurate**<br>• **1,7-Dimethylurate**<br>• **1-Methylxanthine**<br>• **Caffeine**<br>• **Catechol sulfate**<br>• **N-(2-furoyl)glycine**<br>• **Quinate**<br>• **Theophylline**<br>• **Trigonelline**	
	Coffee, caffeinated	Habitual intake (dose not reported); assessed by FFQ				Urine	• **1,3,7-Trimethylurate**<br>• **1,3-Dimethylurate**<br>• **1,7-Dimethylurate**<br>• **1-Methylurate**<br>• **1-Methylxanthine**<br>• **AAMU**	

Summary of Untargeted Studies Presenting Candidate FIBs for Fermented Foods								
**Fermented Food**	**Intervention**	**Dose**	**Study Design**	***n***	**Analytical Method**	**Biosample**	**Candidate FIBs[a]**	**Reference**
							• **AFMU**<br>• **Caffeine**<br>• **Catechol sulfate**<br>• **Hippurate**<br>• **Homovanillate sulfate**<br>• **N-(2-furoyl)glycine**<br>• **Nicotinate**<br>• **Pseudouridine**<br>• **Quinate**<br>• **Theophylline**<br>• **Trigonelline**	
	Coffee, decaffeinated	Habitual intake (dose not reported); assessed by FFQ				Serum	• **1,7-Dimethylurate**<br>• **3-methoxytyrosine**	
	Wine	Habitual intake (dose not reported); assessed by FFQ				Urine	• **2,3-Dihydroxyisovalerate**<br>• **2-Isopropylmalate**<br>• **Nicotine**	
Beer	Strong lager vs. regular lager vs. light/alcohol-free beer vs. control soft drink	330 mL	3-day, randomized, controlled, single-blind, crossover	37 healthy adults	U(H)PLC-QTOF-MS	Plasma	• Hydroxy alloiso-cohumulone, cohumulinone<br>• 2-ethyl malate<br>• Cysteine conjugate of NO2 or CH2O2 adducted iso-ad/humulone<br>• Cysteine conjugate of NO2 or CH2O2 adducted iso-cohumulone<br>• Dihydroxylated iso-ad/humulone<br>• Dihydroxylated iso-ad/humulone II<br>• Hordenine<br>• Hydroxy alloiso-ad/humulones, humulinone<br>• Iso/leucine<br>• Iso-cohumulone<br>• Iso-n/ad-humulone<br>• Iso-tricycload/humene<br>• Iso-tricyclohumene<br>• Maltose<br>• N-methyl tyramine sulfate<br>• NO or CH2O adduct of iso-ad/humulone<br>• NO or CH2O adduct of iso-cohumulone<br>• NO2 or CH2O2 adduct of iso-cohumulone<br>• NO2 or CH2O2 conjugate adduct of iso-ad/humulone<br>• Pyroglutamyl proline	(53)

Summary of Untargeted Studies Presenting Candidate FIBs for Fermented Foods								
**Fermented Food**	**Intervention**	**Dose**	**Study Design**	***n***	**Analytical Method**	**Biosample**	**Candidate FIBs[a]**	**Reference**
							• Tetra-cycload/humol<br>• Tetra-cyclocohumol, tricyclohumol<br>• Tricycload/humol, double hydroxylated iso-cohumulone<br>• Tyrosine	
Beer	Beer, alcoholic	660 mL/day	4-week, randomized, controlled, crossover	33 men with high CV risk	HPLC-ESI-MS/MS (targeted plasma acylcarnitines)	Plasma	• Acylcarnitines	(54)
					LC-MS (untargeted)		• **1,2,3,4-Tetrahydro-1-methyl-beta-carboline-3-carboxylic acid**<br>• **2,3-Dihydroxy-3-methylvaleric acid**<br>• **2-Phenylethanol glucuronide**<br>• **4-Guanidinobutanoic acid**<br>• **Cohumulone**<br>• **Ethyl glucuronide**<br>• **Ethyl sulfate**<br>• **Humulinone**<br>• **Hydroxyadipic acid**<br>• **Oxyhumulinic acid**	
	Beer, non-alcoholic	660 mL/day			HPLC-ESI-MS/MS (targeted plasma acylcarnitines)		• Acylcarnitines	
					LC-MS (untargeted)		• **2,3-dihydroxy-3-methylvaleric acid**<br>• **4-Guanidinobutanoic acid**<br>• **Cohumulone**<br>• **Humulinone**<br>• **Hydroxyadipic acid**<br>• **Oxyhumulinic acid**	
Beer	Habitual dietary intake	Frequency of intake for 137 foods, including beer (from FFQ)	Association study	491 volunteers from the Prostate, Lung, Colorectal, and Ovarian Cancer Screening Trial	HILIC-QQQ-MRM (Untargeted)	Serum	• **Beer: C24:0 sphingomyelin**	(55)
Rice beer	Rice beer drinkers vs. non-drinkers In Ahom and Bodo ethnic groups	Drinkers or non-drinkers of rice beer (from questionnaire)	Cross-sectional	134 healthy volunteers	GC-MS (Untargeted)	Feces	• Propanoic, butyric, cis-vaccenic acids was higher in Ahom non-drinkers vs. drinkers<br>• Butyric acid, rhamnose, arabinose, glycine, hydroxycinnamic acid, indole, formic acid, ursodeoxycholic acid, acetic acid, and benzoic acid was higher in Bodo non-drinkers vs. drinkers	(56)

Summary of Untargeted Studies Presenting Candidate FIBs for Fermented Foods								
**Fermented Food**	**Intervention**	**Dose**	**Study Design**	***n***	**Analytical Method**	**Biosample**	**Candidate FIBs[a]**	**Reference**
							(250 metabolites were detected, but only 40 metabolites from microbial origin were considered)	
Post-fermented tea	Tea, pu-erh	200 mL/day	2-week, randomized, crossover	20 healthy adults	U(H)PLC-QTOF-MS	Urine	• **1,3-Dimethylurate**<br>• **1,7-Methyluric acid**<br>• **1-Methyluric acid**<br>• **1-Methylxanthine**<br>• **2-Hydroxybenzoic acid**<br>• **2-Methoxyphenol**<br>• **3,5-Hydroxybenzoic acid**<br>• **3-Hydroxyphenylacetic acid**<br>• **4-Aminobutanoic acid**<br>• **4-Aminobutanoic acid**<br>• **4-Hydroxy-3-methoxyphenylacetic acid**<br>• **Aminomalonic acid**<br>• **Caffeine**<br>• **Epigallocatechin**<br>• **Hippurate**<br>• **Nicotinic acid**<br>• **Ornithine**<br>• **Paraxanthine**<br>• **Phenol**<br>• **Theobromine**<br>• **Theophylline**<br>• **Valine**	(57)
General fermented foods	Fermented food intake frequency (never, <1X/week, 1-2X/week, 3-5X/week, daily)	Habitual diet and activites; dose not reported	Cross-sectional	7 healthy adults who ferment their own foods	U(H)PLC-MS	Multiple (biofilm, fermented food, forehead, indoor surface, hands, mouth, stool)	• **Avobenzone (skin)**<br>• **Bacteriopheophytin (kimchi)**<br>• **Cholesterol and derivatives (skin)**<br>• **Gingerol (foods, indoor surfaces)**<br>• **Pheophytin A (foods of vegetable origin)**<br>• **Piperine (food, stool, indoor surfaces, skin)**<br>• **Plant flavonoids, lipids, plant sterols**<br>• **Polanrazine B (food, stool)**<br>• **Procyanidin B2 (biofilm, food, indoor surface, skin, stool)**<br>• **Pyropheophytin (kimchi)**	(58)

Abbreviations: AAMU, 5-Acetylamino-6-amino-3-methyluracil; AFMU, 5-Acetylamino-6-formylamino-3-methyluracil; AMMU, 6-amino-5[N-methylformylamino]-1-methyluracil; AR, alkenylresorcinol; DCER, dihydroceramides; DHBA, 3,5-dihydroxybenzoic acid; DHPPA, 3-(3,5-dihydroxyphenyl)-1-propanoic acid); DHPPTA, 5-(3,5-dihydroxyphenyl) pentanoic acid; DHPV, 5-(3,4-dihydroxyphenyl)-γ-valerolactone; ESI, electrospray ionization; FA, fatty acid; FFQ, food frequency questionnaire; FIB = food intake biomarker; FIE, flow injection electrospray; GC, gas chromatography; HBOA, 2-Hydroxy-1,4-benzoxazin-3-one; HHPAA, 2-Hydroxy-N-(2-hydroxyphenyl) acetamide; HILIC, hydrophilic interaction liquid chromatography; HPAA, N-(2-hydroxyphenyl) acetamide; HPLC, high-performance liquid chromatography; HS, headspace; IMS, ion mobility spectrometry; LC, liquid chromatography; LCER, lactosylceramides; MHPV, 3′-methoxy-4′-hydroxyphenylvalerolactone; MS, mass spectrometry; MRM, multiple reaction monitoring; MS/MS, tandem mass spectrometry; NMR, nuclear magnetic resonance; PC, phosphatidylcholine; PE, phosphatidylethanolamine; QQQ, triple quadrupole; QTOF, quadrupole time-of-flight; SPME, solid phase microextraction; TAG, triacylglycerol; TOF, time-of-flight; U(H)PLC, ultra-high performance liquid chromatography.

[a] Candidate FIBs that are significantly increased compared to control or baseline in each study are bolded. Candidate FIBs that are detected in the biosample, but not statistically significant, are not bolded.

References

1. Andersen M-BS, Kristensen M, Manach C, Pujos-Guillot E, Poulsen SK, Larsen TM, et al. Discovery and validation of urinary exposure markers for different plant foods by untargeted metabolomics. Anal Bioanal Chem. 2014;406(7):1829-44.
2. Andersen M-BS, Rinnan A, Manach C, Poulsen SK, Pujos-Guillot E, Larsen TM, et al. Untargeted metabolomics as a screening tool for estimating compliance to a dietary pattern. J Proteome Res. 2014;13(3):1405-18.
3. Garcia-Aloy M, Llorach R, Urpi-Sarda M, Jauregui O, Corella D, Ruiz-Canela M, et al. A metabolomics-driven approach to predict cocoa product consumption by designing a multimetabolite biomarker model in free-living subjects from the PREDIMED study. Mol Nutr Food Res. 2015;59(2):212-20.
4. Llorach R, Urpi-Sarda M, Jauregui O, Monagas M, Andres-Lacueva C. An LC-MS-based metabolomics approach for exploring urinary metabolome modifications after cocoa consumption. J Proteome Res. 2009;8(11):5060-8.
5. Llorach R, Urpi-Sarda M, Tulipani S, Garcia-Aloy M, Monagas M, Andres-Lacueva C. Metabolomic fingerprint in patients at high risk of cardiovascular disease by cocoa intervention. Mol Nutr Food Res. 2013;57(6):962-73.
6. Llorach-Asuncion R, Jauregui O, Urpi-Sarda M, Andres-Lacueva C. Methodological aspects for metabolome visualization and characterization A metabolomic evaluation of the 24 h evolution of human urine after cocoa powder consumption. J Pharm Biomed Anal. 2010;51(2):373-81.
7. Ostertag LM, Philo M, Colquhoun IJ, Tapp HS, Saha S, Duthie GG, et al. Acute Consumption of Flavan-3-ol-Enriched Dark Chocolate Affects Human Endogenous Metabolism. J Proteome Res. 2017;16(7):2516-26.
8. Pallister T, Jennings A, Mohney RP, Yarand D, Mangino M, Cassidy A, et al. Characterizing blood metabolomics profiles associated with self-reported food intakes in female twins. PLoS One. 2016;11(6).
9. Hakeem Said I, Truex JD, Heidorn C, Retta MB, Petrov DD, Haka S, et al. LC-MS/MS based molecular networking approach for the identification of cocoa phenolic metabolites in human urine. Food Res Int. 2020;132.
10. Martin F-PJ, Rezzi S, Peré-Trepat E, Kamlage B, Collino S, Leibold E, et al. Metabolic effects of dark chocolate consumption on energy, gut microbiota, and stress-related metabolism in free-living subjects. J Proteome Res. 2009;8(12):5568-79.
11. Rezzi S, Ramadan Z, Martin F-PJ, Fay LB, van Bladeren P, Lindon JC, et al. Human metabolic phenotypes link directly to specific dietary preferences in healthy individuals. J Proteome Res. 2007;6(11):4469-77.
12. Wang Y, Gapstur SM, Carter BD, Hartman TJ, Stevens VL, Gaudet MM, et al. Untargeted Metabolomics Identifies Novel Potential Biomarkers of Habitual Food Intake in a Cross-Sectional Study of Postmenopausal Women. J Nutr. 2018;148(6):932-43.
13. Beckmann M, Lloyd AJ, Haldar S, Seal C, Brandt K, Draper J. Hydroxylated phenylacetamides derived from bioactive benzoxazinoids are bioavailable in humans after habitual consumption of whole grain sourdough rye bread. Mol Nutr Food Res. 2013;57(10):1859-73.
14. Bondia-Pons I, Nordlund E, Mattila I, Katina K, Aura AM, Kolehmainen M, et al. Postprandial differences in the plasma metabolome of healthy Finnish subjects after intake of a sourdough fermented endosperm rye bread versus white wheat bread. Nutr J. 2011;10(1).
15. Garcia-Aloy M, Llorach R, Urpi-Sarda M, Tulipani S, Salas-Salvadó J, Martínez-González MA, et al. Nutrimetabolomics fingerprinting to identify biomarkers of bread exposure in a free-living population from the PREDIMED study cohort. Metabolomics. 2014;11(1):155-65.
16. Hanhineva K, Lankinen MA, Pedret A, Schwab U, Kolehmainen M, Paananen J, et al. Nontargeted metabolite profiling discriminates diet-specific biomarkers for consumption of whole grains, fatty fish, and bilberries in a randomized controlled trial. J Nutr. 2015;145(1):7-17.
17. Lankinen M, Schwab U, Seppanen-Laakso T, Mattila I, Juntunen K, Mykkanen H, et al. Metabolomic Analysis of Plasma Metabolites That May Mediate Effects of Rye Bread on Satiety and Weight Maintenance in Postmenopausal Women. J Nutr. 2011;141(1):31-6.
18. Keski-Rahkonen P, Kolehmainen M, Lappi J, Micard V, Jokkala J, Rosa-Sibakov N, et al. Decreased plasma serotonin and other metabolite changes in healthy adults after consumption of wholegrain rye: an untargeted metabolomics study. Am J Clin Nutr. 2019;109(6):1630-9.
19. Zhu Y, Wang P, Sha W, Sang S. Urinary Biomarkers of Whole Grain Wheat Intake Identified by Non-targeted and Targeted Metabolomics Approaches. Sci Rep. 2016;6.
20. Hanhineva K, Keski-Rahkonen P, Lappi J, Katina K, Pekkinen J, Savolainen O, et al. The postprandial plasma rye fingerprint includes benzoxazinoid-derived phenylacetamide sulfates. J Nutr. 2014;144(7):1016-22.
21. Bondia-Pons I, Barri T, Hanhineva K, Juntunen K, Dragsted LO, Mykkänen H, et al. UPLC-QTOF/MS metabolic profiling unveils urinary changes in humans after a whole grain rye versus refined wheat bread intervention. Mol Nutr Food Res. 2013;57(3):412-22.
22. Cornelis MC, Erlund I, Michelotti GA, Herder C, Westerhuis JA, Tuomilehto J. Metabolomic response to coffee consumption: application to a three-stage clinical trial. J Intern Med. 2018;283(6):544-57.
23. Guertin KA, Loftfield E, Boca SM, Sampson JN, Moore SC, Xiao Q, et al. Serum biomarkers of habitual coffee consumption may provide insight into the mechanism underlying the association between coffee consumption and colorectal cancer. Am J Clin Nutr. 2015;101(5):1000-11.
24. Heinzmann SS, Holmes E, Kochhar S, Nicholson JK, Schmitt-Kopplin P. 2-Furoylglycine as a Candidate Biomarker of Coffee Consumption. J Agric Food Chem. 2015;63(38):8615-21.
25. Lang R, Wahl A, Stark T, Hofmann T. Urinary N-methylpyridinium and trigonelline as candidate dietary biomarkers of coffee consumption. Mol Nutr Food Res. 2011;55(11):1613-23.

26. Redeuil K, Smarrito-Menozzi C, Guy P, Rezzi S, Dionisi F, Williamson G, et al. Identification of novel circulating coffee metabolites in human plasma by liquid chromatography-mass spectrometry. J Chromatogr A. 2011;1218(29):4678-88.
27. Rothwell JA, Fillatre Y, Martin J-F, Lyan B, Pujos-Guillot E, Fezeu L, et al. New biomarkers of coffee consumption identified by the non-targeted metabolomic profiling of cohort study subjects. PLoS One. 2014;9(4):e93474.
28. Kuang A, Erlund I, Herder C, Westerhuis JA, Tuomilehto J, Cornelis MC. Lipidomic Response to Coffee Consumption. Nutrients. 2018;10(12).
29. Mack CI, Egert B, Liberto E, Weinert CH, Bub A, Hoffmann I, et al. Robust Markers of Coffee Consumption Identified Among the Volatile Organic Compounds in Human Urine. Mol Nutr Food Res. 2019;63(10):e1801060.
30. Papandreou C, Hernandez-Alonso P, Bullo M, Ruiz-Canela M, Yu E, Guasch-Ferre M, et al. Plasma Metabolites Associated with Coffee Consumption: A Metabolomic Approach within the PREDIMED Study. Nutrients. 2019;11(5).
31. Rothwell JA, Keski-Rahkonen P, Robinot N, Assi N, Casagrande C, Jenab M, et al. A Metabolomic Study of Biomarkers of Habitual Coffee Intake in Four European Countries. Mol Nutr Food Res. 2019;63(22):e1900659.
32. Lloyd AJ, Beckmann M, Haldar S, Seal C, Brandt K, Draper J. Data-driven strategy for the discovery of potential urinary biomarkers of habitual dietary exposure. Am J Clin Nutr. 2013;97(2):377-89.
33. Chau YP, Au PCM, Li GHY, Sing CW, Cheng VKF, Tan KCB, et al. Serum Metabolome of Coffee Consumption and its Association With Bone Mineral Density: The Hong Kong Osteoporosis Study. J Clin Endocrinol Metab. 2020;105(3).
34. Hang D, Zeleznik OA, He X, Guasch-Ferre M, Jiang X, Li J, et al. Metabolomic Signatures of Long-term Coffee Consumption and Risk of Type 2 Diabetes in Women. Diabetes Care. 2020.
35. Shi L, Brunius C, Johansson I, Bergdahl IA, Rolandsson O, van Guelpen B, et al. Plasma metabolite biomarkers of boiled and filtered coffee intake and their association with type 2 diabetes risk. J Intern Med. 2020;287(4):405-21.
36. Esteban-Fernandez A, Ibanez C, Simo C, Bartolome B, Moreno-Arribas MV. An Ultrahigh-Performance Liquid Chromatography-Time-of-Flight Mass Spectrometry Metabolomic Approach to Studying the Impact of Moderate Red-Wine Consumption on Urinary Metabolome. J Proteome Res. 2018;17(4):1624-35.
37. Heinzmann SS, Merrifield CA, Rezzi S, Kochhar S, Lindon JC, Holmes E, et al. Stability and robustness of human metabolic phenotypes in response to sequential food challenges. J Proteome Res. 2012;11(2):643-55.
38. Jiménez-Girón A, Muñoz-González I, Martín-Álvarez PJ, Moreno-Arribas MV, Bartolomé B. Towards the fecal metabolome derived from moderate red wine intake. Metabolites. 2014;4(4):1101-18.
39. Jiménez-Girón A, Ibanez C, Cifuentes A, Simo C, Muñoz-González I, Martin-Álvarez PJ, et al. Faecal metabolomic fingerprint after moderate consumption of red wine by healthy subjects. J Proteome Res. 2015;14(2):897-905.
40. Vázquez-Fresno R, Llorach R, Alcaro F, Rodríguez MA, Vinaixa M, Chiva-Blanch G, et al. 1H-NMR-based metabolomic analysis of the effect of moderate wine consumption on subjects with cardiovascular risk factors. Electrophoresis. 2012;33(15):2345-54.
41. Vázquez-Fresno R, Llorach R, Urpi-Sarda M, Khymenets O, Bulló M, Corella D, et al. An NMR metabolomics approach reveals a combined-biomarkers model in a wine interventional trial with validation in free-living individuals of the PREDIMED study. Metabolomics. 2015;11(4):797-806.
42. Vázquez-Fresno R, Llorach R, Perera A, Mandal R, Feliz M, Tinahones FJ, et al. Clinical phenotype clustering in cardiovascular risk patients for the identification of responsive metabotypes after red wine polyphenol intake. J Nutr Biochem. 2016;28:114-20.
43. Hjerpsted JB, Ritz C, Schou SS, Tholstrup T, Dragsted LO. Effect of cheese and butter intake on metabolites in urine using an untargeted metabolomics approach. Metabolomics. 2014;10(6):1176-85.
44. Münger LH, Trimigno A, Picone G, Freiburghaus C, Pimentel G, Burton KJ, et al. Identification of Urinary Food Intake Biomarkers for Milk, Cheese, and Soy-Based Drink by Untargeted GC-MS and NMR in Healthy Humans. J Proteome Res. 2017;16(9):3321-35.
45. Zheng H, Yde CC, Clausen MR, Kristensen M, Lorenzen J, Astrup A, et al. Metabolomics investigation to shed light on cheese as a possible piece in the French paradox puzzle. J Agric Food Chem. 2015;63(10):2830-9.
46. Fuchsmann P, Tena Stern M, Münger LH, Pimentel G, Burton KJ, Vionnet N, et al. Nutrivolatilomics of Urinary and Plasma Samples to Identify Candidate Biomarkers after Cheese, Milk and Soy-Based Drink Intake in Healthy Humans. J Proteome Res. 2020.
47. Pimentel G, Burnand D, Münger LH, Pralong FP, Vionnet N, Portmann R, et al. Identification of Milk and Cheese Intake Biomarkers in Healthy Adults Reveals High Interindividual Variability of Lewis System-Related Oligosaccharides. J Nutr. 2020;150(5):1058-67.
48. Trimigno A, Münger L, Picone G, Freiburghaus C, Pimentel G, Vionnet N, et al. GC-MS Based Metabolomics and NMR Spectroscopy Investigation of Food Intake Biomarkers for Milk and Cheese in Serum of Healthy Humans. Metabolites. 2018;8(2).
49. Pedersen SMM, Nielsen NC, Andersen HJ, Olsson J, Simren M, Ohman L, et al. The serum metabolite response to diet intervention with probiotic acidified milk in irritable bowel syndrome patients is indistinguishable from that of non-probiotic acidified milk by 1H NMR-based metabonomic analysis. Nutrients. 2010;2(11):1141-55.
50. Pedersen SMM, Nebel C, Nielsen NC, Andersen HJ, Olsson J, Simren M, et al. A GC-MS-based metabonomic investigation of blood serum from irritable bowel syndrome patients undergoing intervention with acidified milk products. Eur Food Res Technol. 2011;233(6):1013-21.
51. Pimentel G, Burton KJ, von Ah U, Butikofer U, Pralong FP, Vionnet N, et al. Metabolic Footprinting of Fermented Milk Consumption in Serum of Healthy Men. J Nutr. 2018;148(6):851-60.
52. Playdon MC, Sampson JN, Cross AJ, Sinha R, Guertin KA, Moy KA, et al. Comparing metabolite profiles of habitual diet in serum and urine. Am J Clin Nutr. 2016;104(3):776-89.

53. Gürdeniz G, Jensen MG, Meier S, Bech L, Lund E, Dragsted LO. Detecting Beer Intake by Unique Metabolite Patterns. J Proteome Res. 2016;15(12):4544-56.
54. Quifer-Rada P, Chiva-Blanch G, Jauregui O, Estruch R, Lamuela-Raventos RM. A discovery-driven approach to elucidate urinary metabolome changes after a regular and moderate consumption of beer and nonalcoholic beer in subjects at high cardiovascular risk. Mol Nutr Food Res. 2017;61(10).
55. Mazzilli KM, McClain KM, Lipworth L, Playdon MC, Sampson JN, Clish CB, et al. Identification of 102 Correlations between Serum Metabolites and Habitual Diet in a Metabolomics Study of the Prostate, Lung, Colorectal, and Ovarian Cancer Trial. J Nutr. 2020;150(4):694-703.
56. Deb D, Das S, Adak A, Khan MR. Traditional rice beer depletes butyric acid-producing gut bacteria Faecalibacterium and Roseburia along with fecal butyrate levels in the ethnic groups of Northeast India. 3 Biotech. 2020;10(6):283.
57. Xie G, Zhao A, Zhao L, Chen T, Chen H, Qi X, et al. Metabolic fate of tea polyphenols in humans. J Proteome Res. 2012;11(6):3449-57.
58. Quinn RA, Navas-Molina JA, Hyde ER, Song SJ, Vazquez-Baeza Y, Humphrey G, et al. From Sample to Multi-Omics Conclusions in under 48 Hours. mSystems. 2016;1(2).
