# Supplementary material for: A systematic review to identify biomarkers of intake for fermented food products
Source: Genes Nutr. 2021 Apr 21;16:5. doi: 10.1186/s12263-021-00686-4 (PMC8058972; doi:10.1186/s12263-021-00686-4)
Supplement: Supplementary file 4 — Additional File 4. Summary of Targeted Studies Presenting Candidate FIBs for Fermented Foods.pdf. [file 12263_2021_686_MOESM4_ESM.pdf]

Additional File 4

Summary of Targeted Studies Presenting Candidate FIBs for Fermented Foods								
**Fermented Food**	**All interventions**	**Dose**	**Study Design**	***n***	**Analytical Method**	**Biosample**	**Candidate FIBs[a]**	**Reference**
Beer	Beer vs. Non-alcoholic control drink	330 mL	Acute, randomized, controlled, crossover	19 healthy volunteers (n=4 urine, n=3 plasma, n=1 serum)	U(H)PLC-MS/MS (Targeted)	Serum, plasma, urine	• L-Tartaric acid<br>• Ethyl sulfate<br>• Ethyl-beta-D-gulcuronide<br>• Indoxyl sulfate<br>• Cresol sulfate<br>• Resveratrol<br>• Estrone sulfate<br>• Dihydroepiandrosterone sulfate<br>• (Isoxanthohumol)*<br>• (Isocohumulone)*<br>• (3-nitrotyrosine)*<br>• (Indole-3-lactic acid)*<br>• (Cortisol)*<br>• (Cortisol sulfate)*<br>(* Detected but not validated)	(1)
Beer	Amstel beer (RIAA) vs. Hahn Premium Light beer (TIAA) vs. Coopers Clear beer (HIAA)	470-770 mL (Amstel), 850-1500 mL (Hahn), 500-850 mL (Coopers Clear)	Acute intervention	5 healthy volunteers	U(H)PLC-MS/MS (Targeted)	Urine	• Iso-alpha-acids (isohumulones)<br>• Rho-iso-alpha-acids (RIAA)<br>• Tetrahydro-iso-alpha-acids (TIAA)<br>• Hexahydro-iso-alpha-acids (HIAA)	(2)
Beer	Beer	330 mL	Acute intervention	10 healthy volunteers	LC-ESI-MS/MS (Targeted)	Urine	• Isoxanthohumol<br>• Xanthohumol<br>• 8-prenylnaringenin	(3)
Beer	Beer + 300 g allopurinol (anti-hyperuricemic agent) vs. Beer alone vs. Allopurinol alone	10 mL/kg body weight	Acute crossover	5 healthy volunteers	Not reported (Targeted)	Plasma, urine	• Hypoxanthine<br>• Xanthine<br>• Uric acid	(4)
Beer	Volt-Damm beer	330 mL (women); 660 mL (men)	Acute intervention	7 healthy volunteers	Melatonin ELISA and HPLC (Targeted)	Serum	• Melatonin	(5)
Beer	Beer vs. Vodka and carbonated water	1 L (standardized to 48 g alcohol content)	Acute crossover	7 healthy volunteers	GC-MS/MS (Targeted)	Urine, serum	• Mevalonic acid	(6)

Summary of Targeted Studies Presenting Candidate FIBs for Fermented Foods								
**Fermented Food**	**All interventions**	**Dose**	**Study Design**	***n***	**Analytical Method**	**Biosample**	**Candidate FIBs[a]**	**Reference**
Beer	Little Creatures Pale Ale (high-hopped) vs. Erdinger Weissbier (low-hopped)	613 to 802 mL	Acute crossover	5 healthy men	U(H)PLC-MS/MS (Targeted)	Whole blood, urine	• Iso-alpha-acids (isohumulones)	(7)
Beer	1 Beer, 2 Beers, 3 Beers (male); 1 Beer, 1.5 Beers, 2 Beers (female)	330 mL, 660 mL, 990 mL (males); 330 mL, 495 mL, 660 mL (females)	Acute dose-response, randomized, crossover	41 healthy volunteers	LC-MS/MS (Targeted)	Urine	• Isoxanthohumol	(8)
	Beer vs. Gin vs. non-alcoholic beer	660 mL (beer); 92 mL (gin) (standardized to 30 g ethanol/day); 990 mL non-alcoholic beer (equivalent amount of polyphenols)	4-week randomized, open, controlled, crossover	33 males with high CV risk				
	Beer	No beer drinkers vs. intermittent/daily beer drinkers (22 to 825 mL/day) (from FFQ)	Parallel-group, multicenter, controlled, randomized 5-year clinical trial	46 volunteers from PREDIMED cohort				
Beer	Non-alcoholic beer	2.5 L	Acute intervention	4 healthy volunteers	LC-MS/MS (Targeted)	Urine	• Ethyl glucuronide<br>• Ethyl sulfate	(9)
Beer	Low-alcohol beer	4 L over 4 hours	Acute intervention	5 healthy men	HPLC-PDA (Targeted)	Urine	• Ferulic acid (total, free and glucuronidated)<br>• 4-Dydroxy-3-methoxy-cinnamic acid	(10)
Beer	Beer	0.5 g/kg body weight (594 to 986 mL)	Acute intervention	6 healthy volunteers	LC-MS (Targeted)	Urine	• Ethanol<br>• Ethyl glucuronide	(11)
Beer	Light beer	330 mL	Acute intervention	8 healthy volunteers	Enzymatic method (Targeted)	Capillary blood	• Ethanol	(12)
	Beer on empty stomach	660 mL	Acute intervention	9 healthy men				

Summary of Targeted Studies Presenting Candidate FIBs for Fermented Foods								
**Fermented Food**	**All interventions**	**Dose**	**Study Design**	***n***	**Analytical Method**	**Biosample**	**Candidate FIBs[a]**	**Reference**
	Beer with meal	660 mL	Acute intervention	9 healthy men				
Beer	Low-alcohol beer (0.5%) vs. low-alcohol beer (0.9%)	3 L	Acute, randomized, crossover	20 healthy men	Alcohol dehydrogenase method (Targeted)	Blood	• Ethanol	(13)
Beer	Beer vs. whisky vs. white wine vs. dry sherry	~614 mL (beer); 240 mL (beer); 157 mL (sherry); 66.6 mL (whisky); standardised to 0.3 g/kg body weight ethanol	Acute, randomized, crossover	11 healthy men	Breathalyser (Targeted)	Blood	• Ethanol	(14)
Beer	Non-drinkers vs. drinkers after a drinking party	0 (non-drinkers); not reported (drinking party)	Cross-sectional	40 healthy men (non-drinkers); 13 healthy men (drinking party)	GC-MS (Targeted)	Urine	• 1-Methyl-1,2,3,4-tetrahydro-beta-carboline<br>• 1,2,3,4-Tetrahydro-beta-carboline<br>• Tryptamine (after administration of dideuterated L-tryptophan)	(15)
	Beer vs. whisky	500 mL (beer); 68 mL (whisky)	Acute intervention	4 healthy men				
Beer	Beer	16 mL/kg body weight	Acute intervention	4 healthy men (3 flushers and 1 non-flusher)	HS-GC (Targeted)	Blood, urine	• Ethanol<br>• Acetaldehyde<br>• Acetate	(16)
Beer	Beer	16 mL/kg body weight (non-flushers); 8 mL/kg (flushers)	Acute intervention	4 healthy men	HS-GC-FID (Targeted)	Urine	• Free and bound ethanol<br>• Free and bound acetaldehyde<br>• Free and bound acetate<br>• Free and bound acetone<br>• Free and bound methanol	(17)
Beer	Beer vs. rum vs. carbonate mixed rum	275 mL (beer); 40 mL (rum); standardised to 12 g ethanol	Acute parallel	30 healthy volunteers	TDx Abbott analyzer (Targeted)	Plasma	• Ethanol	(18)
Beer	Tusker or Pilsner beer	8-15 beers (500 mL each) for me; 8-12 beers for women	Acute intervention	17 volunteers	GC (detector not specified) (Targeted)	Urine, blood	• Alcohol	(19)

Summary of Targeted Studies Presenting Candidate FIBs for Fermented Foods								
**Fermented Food**	**All interventions**	**Dose**	**Study Design**	***n***	**Analytical Method**	**Biosample**	**Candidate FIBs[a]**	**Reference**
Beer	Beer	762 to 1000 mL	Acute intervention	4 healthy volunteers	HS-GC-EI-MS (ethanol), U(H)PLC-ESI-MS/MS (hordenine) (Targeted)	Plasma	• Ethanol<br>• Hordenine (N,N-dimethyltyramine) and glucuronidated and sulfated conjugates	(20)
Wine, beer	Consumption of 10 alcoholic beverages (light/medium/full strength beer; red/ white/sparkling wine; wine cooler, spirits/liqueurs, spirit-based mixed drinks, sherry/port, and other)	Frequency of intake (never or <1/month, 1–3 times/month, once/week, 2–4 times/week, 5–6 times/week, once/day, 2–3 times/day, 4–5 times/day, >6 times/day) for 10 alcoholic beverages (from FFQ)	Cross-sectional	1785 healthy volunteers from the Childhood Determinants of Adult Health study	NMR (Targeted)	Serum	• Weak positive associations for fatty acids: total fatty acids, saturated fatty acids, MUFA, PUFA, omega-6 PUFA, linoleic acid, omega-3 PUFA, DHA (total alcohol, wine beer)<br>• Weak positive associations for low-molecular weight metabolites: Alanine (total, wine, beer), glutamine (wine), tyrosine (total, wine, beer), glucose (total, wine, beer), pyruvate (beer), glycerol (beer), acetoacetate (beer), beta-hydroxybutyrate (beer), albumin (beer), acetate (beer)	(21)
Wine, beer	Red wine vs. beer vs. Dutch gin vs. water	4 glasses; standardised to 40 g/day alcohol	3-week, randomized, controlled, crossover	12 healthy men	HPLC-FLD/UV (Targeted)	Plasma	• Alpha-tocopherol<br>• Gamma-tocopherol<br>• Lutein<br>• Beta-cryptoxanthin<br>• Lycopene<br>• Alpha-carotene	(22)
Wine, beer	Red wine vs. Lager beer vs. Stout (alcoholic) vs. Stout (non-alcoholic) vs. Water with alcohol	1 drink: 341 mL<br>3 drinks: 3 X 155 mL (wine); 3 X 341 mL (lager beer); 3 X 341 mL (stout), 3 X 341 mL (water)	Acute intervention	12 healthy volunteers (1 drink); 8 healthy volunteers (3 drinks)	Sigma Alcohol kit, ELISA reader (Targeted)	Plasma	• Ethanol	(23)
Wine, beer	Alcohol intake (wine, beer and spirits)	Quartiles of alcohol intake as % total energy intake: Q1:0, Q2: 0.6, Q3: 2.3, Q4: 5.8 (from FFQ)	Association study	1457 healthy volunteers from the IMMIDIET cohort	GC-FID (Targeted)	Plasma	• EPA (women and men)<br>• DHA (women)	(24)

Summary of Targeted Studies Presenting Candidate FIBs for Fermented Foods								
**Fermented Food**	**All interventions**	**Dose**	**Study Design**	***n***	**Analytical Method**	**Biosample**	**Candidate FIBs[a]**	**Reference**
Wine, beer	Beer, white wine, vodka/tonic	All beverages give 0.5 g ethanol/kg body weight (5.1% v/v beer, 12.5% v/v chardonnay, 20% v/v vodka and tonic)	Acute crossover	15 healthy men	HS-GC (Targeted)	Whole blood	• Ethanol	(25)
Wine, beer	Pilsen-type beer, Cabernet Sauvignon red wine, Scotch whisky, cachaca	All beverages give 0.5 g ethanol/kg body weight	Acute crossover	20 healthy volunteers	COBAS INTEGRA Ethanol Kit (Targeted)	Plasma	• Ethanol	(26)
Wine, beer	Swedish vodka and tonic (Absolut), French wine (La Garonne), Swedish export beer (Pripps Export)	All beverages give 1.0 mL ethanol/kg body weight	Acute intervention	6 healthy men	Not reported	Blood	• Ethanol	(27)
Wine, beer	White wine or beer	0.1 or 0.2 L (white wine); 0.33 L or 0.66 L (beer)	Acute intervention	12 healthy volunteers	LC-MS/MS (Targeted)	Urine	• Ethyl glucuronide<br>• Ethyl sulfate	(28)
Wine, beer	Choice of white wine or export beer	50 g or 80 g ethanol	Acute intervention	20 healthy volunteers (‘social drinkers’)	HS-GC, GC-MS, HPLC (Targeted)	Urine	• Ethanol<br>• Methanol<br>• 5-Hydroxytryptophol<br>• 5-Hydroxyindol-3-ylacetic acid	(29)
Wine	Dealcoholized red wine	272 mL/day	4-week, randomized, controlled intervention	36 elderly men	U(H)PLC-MS/MS (Targeted 67 metabolites)	Urine	• (Epi)catechin glucuronides<br>• (Epi)catechin sulfates<br>• DHPV glucuronides<br>• DHPV sulfates<br>• Ethylgallate glucuronides<br>• Ethylgallate sulfate<br>• Methyl(epi)catechin glucuronides<br>• Methyl(epi)catechin sulfates<br>• Methylgallic sulfate<br>• MHPV glucuronide<br>• MHPV sulfates<br>• Hydroxybenzoic acids (especially gallic acid)<br>• Hydroxyphenylacetic acids<br>• Hydroxycinnamic acids	(30)

Summary of Targeted Studies Presenting Candidate FIBs for Fermented Foods								
**Fermented Food**	**All interventions**	**Dose**	**Study Design**	***n***	**Analytical Method**	**Biosample**	**Candidate FIBs[a]**	**Reference**
							• Hydroxyphenylpropanoic acids<br>• Glycinates (vanilloylglycine)<br>• Hydroxyphenylvalerolactones<br>• Enterolactone<br>• Pyrogallol	
Wine	Sparkling wine	0.1 to 0.2 L (9 or 18 g ethanol); 1 volunteer drank 0.54 L (49 g ethanol)	Acute intervention	9 healthy volunteers	LC-ESI-MS/MS (Targeted)	Urine	• Ethyl sulfate<br>• Ethyl glucuronide	(31)
Wine	Merlot red wine	250 mL	Acute intervention	11 healthy men	HPLC-MS/MS (Targeted)	Urine	• Resveratrol (trans-resveratrol) and metabolites (trans-piceid, taxifolin, hexestrol, trans- and cis-resveratrol glucuronides and sulfates)	(32)
Wine	Red wine vs. dealcoholized red wine	272 mL/day	28-day, randomized, controlled, crossover	73 volunteers with high cardiovascular risk	U(H)PLC-MS/MS (Targeted)	Urine	• Resveratrol (trans- and cis-resveratrol) and metabolites (trans-piceid, piceid glucurondies and sulfates, trans- and cis-resveratrol glucuronides and sulfates, dihydroresveratrol and its glucuronides and sulfates)	(33)
Wine	Red wine vs. dealcoholized red wine	272 mL/day	4-week, randomized, controlled, crossover	36 men with high cardiovascular risk	U(H)PLC-MS/MS (Targeted 70 phenolic metabolites)	Plasma, urine	Plasma:<br>• 3-Hydroxyphenylacetic acid<br>• DHPV and glucuronides<br>• Gallic acid<br>• Methyl(epi)catechin glucuronides<br>• Methylgallic acid<br>• Methylgallic sulfate<br>• p-Coumaric acid<br>• (Epi)catechin glucuronides<br>• 3-Hydroxyphenylacetic acid<br>Urine:<br>• (Epi)catechin glucuronides<br>• (Epi)catechin sulfates<br>• 2,4-Dihydroxybenzoic acid<br>• 2,5-Dihydroxybenzoic acid<br>• 2,6-Dihydroxybenzoic acid<br>• 3-Hydroxyphenylacetic acid<br>• DHPV and glucuronides and sulfates<br>• Ethylgallate and glucuronides and sulfates<br>• Gallic acid<br>• Methyl(epi)catechin glucuronides<br>• Methyl(epi)catechin sulfates<br>• Methylgallic acid	(34)

Summary of Targeted Studies Presenting Candidate FIBs for Fermented Foods								
**Fermented Food**	**All interventions**	**Dose**	**Study Design**	***n***	**Analytical Method**	**Biosample**	**Candidate FIBs[a]**	**Reference**
							• Methylgallic sulfate<br>• p-Coumaric acid<br>• Pyrogallol<br>• Resveratrol<br>• Syringic acid<br>• Vanilloylglycine<br>• 3-(3-hydroxyphenyl) propionate<br>• DHBA<br>• 3-Hydroxybenzoic acid<br>• 3-Hydroxyphenylacetic acid<br>• 4-Hydroxybenzoic acid<br>• Caffeic acid<br>• Enterolactone<br>• Ferulic acid<br>• MHPV glucuronides	
Wine	White wine vs. red wine	300 mL/day	15-day, randomized, controlled, parallel	20 healthy volunteers	HPLC electrochemical method (Targeted)	Plasma	• Resveratrol	(35)
Wine	Wine vs. grape juice vs. grape tablets enriched with trans-resveratrol (also compared to separate study with olive oil)	250 mL/day (wine); 1L/day (grape juice); 1 mg resveratrol/day (tablets)	4-day, randomized, controlled, crossover	12 healthy volunteers	GC-MS (Targeted)	Plasma	• Hydroxytyrosol and its metabolite, homovanillic alcohol	(36)
Wine	Red wine vs. no wine	250 mL/day	4-week, randomized, controlled, parallel	41 healthy volunteers	U(H)PLC-ESI-MS/MS (Targeted microbial-derived phenolics)	Feces	• 3,5-Dihydroxybenzoic acid<br>• 3-Hydroxyphenylacetic acid<br>• 3-Phenylpropionate<br>• 4-hydroxy-5-(3,4-dihydroxyphenyl) valeric acid<br>• 4-Hydroxy-5-(phenyl) valeric acid<br>• 4-O-methylgallic acid<br>• DHPV<br>• Protocatechuic acid<br>• Syringic acid<br>• Vanillic acid	(37)
Wine	Wine, grapes, peanuts, and red wine intake (along with total	Continuous intake of resveratrol, resveratrol 3-O-glucoside,	Association study	475 volunteers from the EPIC cohort	U(H)PLC-MS/MS (Targeted)	Urine	• Resveratrol	(38)

Summary of Targeted Studies Presenting Candidate FIBs for Fermented Foods								
**Fermented Food**	**All interventions**	**Dose**	**Study Design**	***n***	**Analytical Method**	**Biosample**	**Candidate FIBs[a]**	**Reference**
	resveratrol intakes)	resveratrol aglycone, wine, grapes, peanuts, and red wine (from 24-h recalls and dietary questionnaire)						
Wine	White wine (and crackers)	250 mL	Acute intervention	10 healthy volunteers	HPLC-ECD (Targeted)	Plasma	• Caffeic acid<br>• Ferulic acid<br>• P-coumaric acid	(39)
Wine	White wine	Volume to achieve ~0.5 g/kg body weight ethanol	Acute intervention	13 healthy volunteers	LC-MS/MS (Targeted)	Serum, urine	• Ethyl glucuronide<br>• Ethyl sulfate	(40)
Wine	Extra virgin olive oil, red wine, extra virgin olive oil + red wine	25 mL (EVOO), 150 mL (wine)	Acute, randomized, crossover	12 healthy volunteers	HPLC-MS/MS (Targeted)	Urine	• Resveratrol (cis, trans, dihydro)<br>• Tyrosol (free, sulfate, and glucoronide)<br>• Hydroxytyrosol (free, sulfate, and glucoronide)<br>• Ethyl glucuronide (wine)	(41)
Wine	Red wine vs. dealcoholized red wine	200 mL	Acute crossover	10 healthy volunteers	HPLC-Coulochem II detector (Targeted)	Plasma	• Caffeic acid	(42)
Wine	Test meal (Milanese beef cutlet and chips) with red wine	300 mL	Acute intervention	10 healthy men	HPLC-UV and HPLC-PDA-MS (Targeted)	Serum	• Trans-resveratrol (and 3- and 4' glucuronides)	(43)
	Red wine	600 mL	Acute intervention	5 healthy volunteers	HPLC-UV-DAD and LC-MS/MS (Targeted)		• Trans-resveratrol (and 3- and 4' glucuronides)	
	'Fat meal' with red wine vs. 'lean meal' with red wine	600 mL	Acute parallel	10 healthy volunteers			• Trans-resveratrol (and 3- and 4' glucuronides)	
Wine	Red wine vs. dealcoholized red wine	272 mL/day	20-day, randomized, controlled, crossover	8 healthy volunteers	U(H)PLC-ESI-MS/MS (Targeted for microbial phenolics)	Feces	• 3,5-Dihydroxybenzoic acid<br>• 3-O-Methylgallic acid<br>• 4-Hydroxy-5-(phenyl) valeric acid<br>• p-Coumaric acid<br>• Phenylpropionic acid<br>• Protocatechuic acid<br>• Syringic acid	(44)

Summary of Targeted Studies Presenting Candidate FIBs for Fermented Foods								
**Fermented Food**	**All interventions**	**Dose**	**Study Design**	***n***	**Analytical Method**	**Biosample**	**Candidate FIBs[a]**	**Reference**
							• Vanillic acid	
Wine	Red wine vs. dealcoholized red wine	120 mL	Acute crossover	9 healthy volunteers	GC-MS (Targeted)	Urine	• Catechin (free) and metabolites (catechin sulfate, glucuronide, glucuronide-sulfate)<br>• 3'-methylcatechin and glucuronides and sulfates	(45)
Wine	Red wine vs. alcohol abstinent	150 mL/day (females); 300 mL/day (males)	3-month, randomized, parallel	44 healthy volunteers	LC-MS/MS (Targeted)	Whole blood	• Phosphatidylethanol<br>• Carbohydrate-deficient transferrin	(46)
Wine	Red wine, ethanol, water	155 mL (wine, first dose), ethanol equivalent to acheive BAC of 40 mg/mL<br><br>310 mL (wine, second dose)	Acute, 2-dose, randomized, single-blind, crossover	13 healthy volunteers	GC (detector not specified) (Targeted)	Plasma	• Resveratrol<br>• Catechin	(47)
Wine	Red wine (at 3 different doses)	100 mL, 200 mL, or 300 mL	Acute, randomized, crossover	21 healthy men	LC-ESI-MS/MS (Targeted)	Urine	• Tartaric acid	(48)
Wine	Mediterranean diet vs. occidental diet, with or without red wine	240 mL/day	3-month, randomized, parallel (1-month wine intake during second month)	42 healthy men	HPLC-electrochemical detection (beta-carotene), spectrophotometry (L-ascorbic acid), Folin-Ciocalteu method (polyphenols) (Targeted)	Plasma, urine (polyphenols)	• Vitamin C<br>• Beta-carotene<br>• Polyphenols (gallic acid equivalents)	(49)
Wine	Wine	Non-wine consumers, intermittent wine consumers, daily wine consumers (from FFQ)	Prospective cohort	1000 volunteers with high CV risk from the PREDIMED cohort	LC-MS/MS (Targeted)	Urine	• Resveratrol metabolites (trans- and cis-resveratrol glucuronides and sulfates)	(50)

Summary of Targeted Studies Presenting Candidate FIBs for Fermented Foods								
**Fermented Food**	**All interventions**	**Dose**	**Study Design**	***n***	**Analytical Method**	**Biosample**	**Candidate FIBs[a]**	**Reference**
Wine	Mencia red wine	1 glass/serving per day (volume not specified)	3-day intervention	25 healthy volunteers who are occasional wine drinkers	HPLC-MS/MS (Targeted)	Plasma	• Resveratrol (trans)	(51)
Wine	Sparking wine vs. gin	300 mL/day (wine); 100 mL (gin), standardized to 30 g ethanol/day	28-day, randomized, controlled, crossover	10 healthy men	LC-MS/MS (Targeted)	Urine	• Total resveratrol metabolites (cis- and trans- resveratrol glucuronides)	(52)
	White wine vs. red wine	200 mL/day, standardized to 20 g ethanol/day	28-day, randomized, controlled, crossover	10 healthy women				
	Wine consumed at 3 different levels	Daily consumption, intermittent consumption, no consumption (from FFQ)	Prospective cohort	52 volunteers from the PREDIMED cohort				
Wine	Red wine vs. dealcholized red wine	120 mL	Acute, randomized, crossover	9 healthy volunteers	GC-MS (Targeted)	Plasma	• Catechin and 3'-O-methylcatechin (and glucuronide and sulfate conjugates)	(53)
Wine	Delacoholized red wine reconstituted with water vs dealcoholized red wine reconstituted with water and ethanol	120 mL	Acute, randomized, crossover	9 healthy volunteers	GC-MS (Targeted)	Plasma	• Total (+)-Catechin	(54)
Wine	Alcohol consumption, wine consumption	3 categories for total alcohol consumption, wine consumption (from FFQ)	Prospective cohort	1045 volunteers with high CV risk from the PREDIMED cohort	GC-MS (hydroxytyrosol); enzyme immunochemical method (ethyl glucuronide) (Targeted)	Urine	• Hydroxytyrosol<br>• Ethyl glucuronide	(55)

Summary of Targeted Studies Presenting Candidate FIBs for Fermented Foods								
**Fermented Food**	**All interventions**	**Dose**	**Study Design**	***n***	**Analytical Method**	**Biosample**	**Candidate FIBs[a]**	**Reference**
Wine	Red wine vs. grape juice vs. red wine extract tablets	250 mL (wine); 1 L (juice); 10 tablets; standardized to 14 ug/kg resveratrol	Acute, randomized, controlled, crossover	11 healthy men	GC-MS (Targeted)	Plasma, urine	• Resveratrol (trans, cis), dihydroresveratrol	(56)
Wine	Red wine	200 mL	Acute intervention	5 healthy men	LC-ESI-MS/MS (Targeted)	Urine	• Tartaric acid<br>• Malic acid<br>• Succinic acid	(57)
Wine	Alcohol consumption	Quartiles of alcohol consumption, continous intake of wine (from FFQ)	Association study	1000 volunteers with high CV risk from the PREDIMED cohort	LC-MS/MS (Targeted)	Urine	• Total resveratrol metabolites (trans- and cis-resveratrol glucuronides and sulfates)	(58)
Wine	Merlot red wine	250 mL	Acute intervention	11 healthy men	LC-ESI-MS/MS (Targeted)	Plasma LDL	• Trans-resveratrol and metabolites (3-O-glucuronide, cis-3-O-glucuronide, cis-3-O-glucoside)	(59)
Wine	Diet without vegetable, fruit, and wine vs. diet with vegetable and fruit vs. diet with wine and no vegetable or fruit vs. diet with wine, vegetable, and fruit	Grouped intakes of fruit, vegetabe, and wine intake (from 24-h recall)	Cross-sectional	180 healthy free-living volunteers	HPLC-FLD (Targeted)	Plasma	• (+)-Catechin	(60)
Wine	Red wine vs. no red wine	375 mL/day	2-week, randomized, controlled, parallel	20 healthy free-living volunteers	HPLC-MS/MS (Targeted)	Plasma	• Total phenolics<br>• (+)-Catechin glucuronide<br>• (-)-Epicatechin glucuronide<br>• Methyl catechin glucuronide<br>• Methyl epicatethin glucuronide	(61)
Wine	Red wine	100, 200, 300 mL	Acute, dose-response intervention	5 healthy men	HPLC-Coulochem II detector (Targeted)	Plasma	• Caffeic acid	(62)

Summary of Targeted Studies Presenting Candidate FIBs for Fermented Foods								
**Fermented Food**	**All interventions**	**Dose**	**Study Design**	***n***	**Analytical Method**	**Biosample**	**Candidate FIBs[a]**	**Reference**
Wine	Table red wine vs. Port red wine	250 mL (table red wine); 150 mL (Port red wine)	Acute crossover	4 healthy men	HPLC-DAD, HPLC-MS (Targeted)	Plasma, urine	• Total plasma anthocyanins and metabolites<br>• Delphinidin-3-O-glucoside<br>• Petunindin-3-O-glucoside<br>• Peonidin-3-O-glucoside<br>• Delphinidin-glucuronide<br>• Peonidin-glucuronide<br>• Malvidin-3-O-beta-glucuronide<br>• Malvidin-3-O-beta-glucoside	(63)
Wine	Oak-aged red wine vs. strawberries vs red raspberries vs. walnuts (all containing ellagitannins)	300 mL (wine); 250 g (strawberries); 225 g (raspberries); 35 g (walnuts)	Acute crossover	40 healthy volunteers	LC-MS/MS (Targeted)	Urine	• Urolithin B derivatives (glucuronide more than aglycone)	(64)
Wine	Wine vs. vodka (diluted in lemon-flavored water) vs. dealcoholised wine vs. placebo (lemon-flavored water)	147 mL	Acute, randomized, controlled, crossover	28 healthy men	Method not reported (Targeted)	Urine	• Hydroxytyrosol<br>• 3'-Dihydroxyphenylacetic acid<br>• Homovanillic acid<br>• 4-Hydroxyphenylacetic acid	(65)
Wine	Wine (alcohol consumption) vs. no wine (alcohol abstinent)	1 glass/day for women (16 g ethanol); 2 glasses/day for men (32 g ethanol)	3-month, randomized, controlled, parallel	44 healthy volunteers	LC-MS/MS (Targeted)	Hair	• Ethyl glucuronide	(66)
Wine	Red wine vs. red grape juice	400 mL	Acute crossover	9 healthy volunteers	HPLC-UV-VIS (Targeted)	Plasma, urine	• Total anthocyanins<br>• Cyanidin-3-glucuronide<br>• Delphinidin-3-glucuronide<br>• Malvidin-3-glucuronide<br>• Peonidin-3-glucuronide<br>• Petunidin-3-glucuronide	(67)
Wine	Red wine vs. delacoholised red wine vs. red grape juice	500 mL	Acute, randomized, crossover	6 healthy men	HPLC-PDA (Targeted)	Plasma, urine	• Malvidin-3-glucoside	(68)
Wine	Wine vs. mature whisky vs. new whisky	100 mL	Acute, randomized, crossover	9 healthy volunteers	Folin Ciocalteu method (Targeted)	Plasma, urine	• Total phenols (gallic acid equivalents)	(69)

Summary of Targeted Studies Presenting Candidate FIBs for Fermented Foods								
**Fermented Food**	**All interventions**	**Dose**	**Study Design**	***n***	**Analytical Method**	**Biosample**	**Candidate FIBs[a]**	**Reference**
Wine	Red wine vs. Red grape juice	400 mL	Acute, randomized, crossover	9 healthy volunteers	HPLC-UV-VIS (Targeted)	Plasma, urine	• Total anthocyanins<br>• Cyanidin-3-glucuronide<br>• Delphinidin-3-glucuronide<br>• Malvidin-3-glucuronide<br>• Peonidin-3-glucuronide<br>• Petunidin-3-glucuronide	(70)
Wine	Wine	250 mL	Acute intervention	7 healthy volunteers	LC-MS/MS (Targeted)	Urine	• Ethyl glucuronide	(71)
Wine	Listerine mouth rinse vs. non-alcoholic wine vs. vodka	4.2 to 7.5 dL (wine); 3.75 mL (vodka)	Acute parallel	12 healthy volunteers	HPLC-MS/MS, HPLC-UV (Targeted)	Oral fluid, whole blood, urine	• Ethyl glucuronide<br>• Ethyl sulfate	(72)
Wine	Pinot noir red wine vs. Cabernet sauvignon red wine vs. dry semillon white wine vs. sauvignon blanc white wine vs. absolute alcohol in water	0.3 g/kg body weight ethanol	Acute, randomized, crossover	108 volunteers with previous illness	GLC (Targeted)	Blood	• Ethanol	(73)
Rice wine	Japanese rice wine (sake) vs. water	0.4 g/kg body weight ethanol	Acute intervention	63 healthy men	GC-FID (Targeted)	Breath, blood	• Alcohol<br>• Acetaldehyde	(74)
Rice wine	Sake vs. rice wine	100 mL (sake); 50 mL (rice wine)	Acute intervention	2 healthy volunteers	1H NMR (Targeted)	Urine	• Ethyl glucoside	(75)
Yoghurt	Yoghurt vs. milk	800 g	Acute, randomized, double-blind, crossover	14 healthy men	GC-MS (Targeted)	Serum	• Lactose<br>• Galactose	(76)
Yoghurt	Probiotic yoghurt with *Bifidobacterium animalis* subsp. *lactis* LKM512 or placebo yoghurt	100 g/day	4-week, double-blind, placebo-controlled, crossover	10 adults with moderate atopic dermatitis	HPLC-Scanning FLD (fecal polyamines); HPLC-Differential Refractometer (fecal	Feces	• Butyrate vs baseline (no increases for other SCFAs measured, acetate, propionate, isobutyrate, valerate, and isovaleate)<br>• Spermidine (also some with increased putrescine, but spermine levels not detected)	(77)

Summary of Targeted Studies Presenting Candidate FIBs for Fermented Foods								
**Fermented Food**	**All interventions**	**Dose**	**Study Design**	***n***	**Analytical Method**	**Biosample**	**Candidate FIBs[a]**	**Reference**
					SCFAs) (Targeted)			
Yoghurt	Yoghurt consumers vs. non-consumers	>200 g/day (consumers), 0 g/day (non-consumers) (from 24-h food records)	Free-living cohort	30 (consumers) ad 21 (non-consumers) from the SU.VI.MAX cohort	GC (detector not specified) (SCFAs); GLC (for bile acids) (Targeted)	Feces	• No significant differences in fecal SCFA (total, acetate, propionate, butyrate, iso-acids, valerate + caproate)<br>• No significant differences in neutral bile acids (cholesterol, coprostanol, cholestanol, coprostanone, beta-sitosterol, copro-beta-sistosterol)<br>• Increase in 7-keto-lithocholic (no significant differences in other acidic bile acids cholic, deoxycholic, ursocholic, 7-keto-deoxycholic, chenodeoxycholic, lithocholic, ursodeoxycholic, 7-keto-lithocholic, sum)	(78)
Yoghurt	Probiotic yoghurt with *Bifidobacterium animalis* subsp. *lactis* LKM512 vs. placebo yoghurt	100 g/day	2-week, placebo-controlled, crossover	7 healthy adults	HPLC-Scanning FLD (fecal polyamines); HPLC-Differential Refractometer (fecal SCFAs) (Targeted)	Feces	• Spermidine (putrescine not increased, spermine levels not detected)<br>• No significant changes in lactate or acetate	(79)
Yoghurt	Fresh yoghurt (>10^7 CFU) vs. heated yoghurt (<10^2 CFU)	500 g/day	15-day, randomized	12 healthy men with lactose malabsorption and 12 healthy men without lactose malabsoption	HS-GC-FID (SCFAs) (Targeted)	Plasma	• Butyrate (in volunteers without lactose malabsorption for fresh yoghurt vs. heated)<br>• Propionate (in volunteers with lactose malabsorption for fresh yoghurt vs. baseline)<br>• No change in acetate	(80)
Yoghurt	Whole milk, commercial unflavored yoghurt, heated yoghurt	400 mL (milk); 450 g (yoghurts)	Acute, randomized, crossover	8 healthy volunteers with lactose malabsorption	Enzymatic assay (Targeted)	Ileal fluid	• Lactose (higher after heated yoghurt than yoghurt)<br>• Galactose (not signficant)<br>• Glucose (not significant)<br>• Hexoses (higher after heated yoghurt than yoghurt)	(81)
Cheese, yoghurt (general dairy)	Total dairy intake	Quintiles of total dairy intake: Q1: 0-2.07, Q2: 2.1-4.1, Q3: 4.13-6.65, Q4:	Association study	659 volunteers without diabetes from the	GC-FID (Targeted)	Serum	• Pentadecanoic acid (15:0)<br>• Trans-palmitoleic acid (trans 16:1n-7) was not considered to be a specific marker for total dairy intake	(82)

Summary of Targeted Studies Presenting Candidate FIBs for Fermented Foods								
**Fermented Food**	**All interventions**	**Dose**	**Study Design**	***n***	**Analytical Method**	**Biosample**	**Candidate FIBs[a]**	**Reference**
		6.69-9.73, Q5: 9.8-31.08 (from FFQ)		IRAS cohort				
Cheese, yoghurt (general dairy)	Dairy fat intake	Continuous levels of dairy fat intake (from 1 week weighted diet records and FFQ)	Association study	81 healthy women	GC with (Targeted)	Subcutaneous adipose tissue	• Pentadecanoic acid (15:0)<br>• Heptadecanoic acid (17:0)	(83)
Cheese, yoghurt (general dairy)	Natural yoghurt, cheddar cheese, semi-skimmed milk, water (dairy as snack)	410 mL	Acute, randomized, crossover	40 overweight men	GC-FID (Targeted)	Plasma	• Amino acids (alpha-amino butyric acid, Ala, Asn, cysteine, Gly, Glu, His, Ile, Leu, Lys, Met, Orn, phe, Pro, Ser, Thr, Trp, Try, Val)	(84)
Cheese, yoghurt (general dairy)	Dairy: Milk (1% fat), yoghurt (1.5% fat), cheese (34% fat)<br>Control: fruit and vegetable juice, cashews and a cookie	3 servings/day (375 mL milk/day, 175 yoghurt/day, 30 g cheese/day)	4 week, randomized, free-living, multi-center, crossover	124 healthy volunteers	GC-FID (Targeted)	Plasma	• Pentadecanoic acid (15:0)<br>• Heptadecanoic acid (17:0)<br>• Trend towards higher cis-9, trans-11-18:2n-6, p=0.06)<br>• Total SFA<br>• 18:3n-6<br>• 22:1n-9<br>• SFA:MUFA and SFA:PUFA	(85)
Cheese, yoghurt (general dairy)	Total dairy, high-fat dairy, low-fat dairy, milk, cream, yoghurt, cheese, butter	Continuous levels of total dairy, high-fat dairy, low-fat dairy, milk, cream, yoghurt, cheese, butter (from FFQ)	Association study	334 control and 1054 intervention volunteers from Food4Me	GC-FID (Targeted)	Dried blood spots	• Pentadecanoic acid (15:0)<br>• Heptadecanoic acid (17:0)	(86)
Cheese, yoghurt (general dairy)	Meat, dairy food, egg, and fish	Quartiles/levels of milk and dairy products, milk, cheese, total meat, red meat, processed meat, white meat, fis hand shellfish, and	Association study	271 participants of the Second Bavarian Food Consumption Survey	LC-MS (Targeted)	Plasma	• Trimethylamine-N-oxide with milk and dairy products, milk (not for other food groups)<br>• No associations for betaine or choline	(87)

Summary of Targeted Studies Presenting Candidate FIBs for Fermented Foods								
**Fermented Food**	**All interventions**	**Dose**	**Study Design**	***n***	**Analytical Method**	**Biosample**	**Candidate FIBs[a]**	**Reference**
		eggs and egg products (from 24-h recalls)						
Cheese, yoghurt	Dairy (total dairy, milk, cheese, yoghurt, cream/butter) intake	Frequency of dairy consumption from never/ <1 time/month to ≥6 times/day (from FFQ)	Association study	2205 volunteers from the Framingham Health Study Offspring cohort and 866 volunteers from the Gen3 cohort	HILIC- and lipid-LC-MS and MS/MS (Targeted and Untargeted)	Plasma	• Cheese: C46:0 triacylglycerol, C54:4 triacylglycerol, C54:5 triacylglycerol, C54:6 triacylglycerol<br>• Yoghurt: C20:5 cholesterol ester	(88)
Cheese, yoghurt, fermented milk (general dairy)	Total milk products, cream, cheese, fermented milk, total milk, ice cream	Quartiles of of total milk products, cream, cheese, fermented milk, total milk, ice cream, and 15:0 and 17:0 (from FFQ)	Prospective case-control nested within larger cohort NSHDS	444 cases of myocardial infarction and 556 controls	GLC (Targeted)	Plasma	• Pentadecanoic acid (15:0)<br>• Heptadecanoic acid (17:0)	(89)
Cheese, soy sauce	Soy sauce with a meal (not further specified) or Appenzeller cheese	9 mL soy sauce; 10 g cheese	Acute intervention	4 volunteers (cheese); 3 volunteers (soy sauce)	HPLC-IS-MS (Targeted)	Urine	• 4-methylspinacemine (4-methyl-4,5,6,7-tetrahydro-1H-imidazo-[4,5-c]pyridine)<br>• 1,4-dimethylspinacemine	(90)
Cheese	Probiotic cheese with *L. plantarum* TENSIA vs. control cheese	50 g/day	3-week, randomized, double-blind, placebo-controlled, parallel	25 hospitalized patients	GC-FID (Targeted)	Urine	• Putrescine and acetylated putrescine in control group decreased vs baseline (no significant differences in other polyamines, tyramine or acetylated spermidine)<br>• Extent of change is significantly higher for probiotic cheese vs control for putrescine and acetylated putrescine	(91)
Cheese	Cheese (from cows with linseed oil added tot heir diet) vs. control cheese	3 X 50 g/week	4-week, randomized, double-blind, crossover	30 healthy, free-living volunteers	GC-FID (Targeted)	Serum	• C18:0 in test cheese group vs baseline<br>• No other significant differences in serum fatty acids compared to baseline for either group (12:0, 14:0, 16:0, 18:0, 16:1, 18:1, 18:2, 20:4)	(92)

Summary of Targeted Studies Presenting Candidate FIBs for Fermented Foods								
**Fermented Food**	**All interventions**	**Dose**	**Study Design**	***n***	**Analytical Method**	**Biosample**	**Candidate FIBs[a]**	**Reference**
Soy	Untreated soymilk vs. beta-glucosidase soymilk vs. fermented soymilk	100 mL	7-day crossover	12 healthy volunteers	LC-MS (Targeted)	Serum, urine	• Isoflavones (daidzein, genistein, glycitein)	(93)
Soy	Breakfast casseroles containing tofu, tempeh, cooked soybeans, or texturized vegetable protein	30 g (texturized vegetable protein); 100 g (tempeh); 100 g (cooked soybeans); 300 g tofu	Acute, randomized, crossover	10 healthy women	HPLC with variable wavelength detector (Targeted)	Plasma, urine, feces	• Isoflavones (daidzein, genistein)	(94)
Soy	Breakfast with natto and spinach vs. detergent-solubilized K1 vs. low-vitamin K diet	200 g (natto) and 400 g (spinach)	Acute crossover	6 healthy men	HPLC-FLD (Targeted)	Serum	• Vitamin K2 (menaquinones); higher than vitamin K1 (phylloquinones in spinach)	(95)
Soy	Fermented soy vs. non-fermented soy	112 g (tempeh); 125 g (soybeans)	9-day, randomized, crossover	22 healthy men	GC-MS (Targeted)	Urine	• Isoflavoids (equol, O-desmethylangolensin, daidzein, genistein), daidzein and genistein recover greater with tempeh diet<br>• Lignans (enterolactone, enterodiol) decreased with soy intake	(96)
Soy	Fermented soymilk containing *Bifodibacterium animalis* Bb-12 vs. non-fermented soymilk	200 mL/day, containing 20, 40, or 80 mg isoflavone/200 mL	14-day, randomized, double-blind, crossover	16 healthy postmenopausal women	HPLC-UV-VIS (Targeted)	Urine	• Isoflavones (genistein, daidzein, glycitein) aglycones, and beta-glucoside isomers	(97)
							• Equol (tendency to increase in the fermented group over time but did not reach significance)	(98)
Soy	Isogen capsules vs. soymilk vs. fermented soybeans	84.8 mg (isogen); 43.8 g (fermented soybeans); 600 mL (soymilk); standardised to 64.8 mg isoflavones	Acute, randomized, controlled, parallel	26 healthy women	HPLC (detector not specified) (Targeted)	Plasma, urine	• Isoflavones (genistein, daidzein) higher urinary recovery in fermented group, longer plasma half-life in fermented and isogen groups	(99)

Summary of Targeted Studies Presenting Candidate FIBs for Fermented Foods								
**Fermented Food**	**All interventions**	**Dose**	**Study Design**	***n***	**Analytical Method**	**Biosample**	**Candidate FIBs[a]**	**Reference**
Soy	Tofu, natto (fermented soybean), soy milk, soy isoflavone supplement	180 g (tofu), 100 g (natto), 300 g (soymilk), 1 supplement, all standardized to 75 mg isoflavones	1-day intervention	20 healthy women (9 equol producers and 11 non-producers)	HPLC-UV (Targeted)	Urine	• Isoflavones (genistein, daidzein)	(100)
Soy	Fermented soy powder (aglycone-rich) with *Aspergillus oryzae* vs. non-fermented soy powder (glycoside-rich)	23 g (standardized to 95 umol isoflavones)	Acute, randomized, double-blind, crossover	11 healthy postmenopausal women	LC-MS/MS (Targeted)	Serum, urine	• Isoflavones (total) increased bioavailability and urinary excretion; trend towards increased in daidzein, genistein, and glycitein as well, no significant difference in equol	(101)
Soy	Stinky tofu	146 g	Acute intervention	36 healthy volunteers (18 equol producers, 18 non-producers)	HPLC-UV (Targeted)	Urine	• S-equol, similar rates of excretion in producers and non-producers<br>• Daidzein and dihydrodaidzein<br>• Total daidzein (daidzein, dihydrodaidzein, S-equol, O-desmethylangolensin)	(102)
Soy	Fermented soymilk with *Lactobacillus casei* Shirota (containing aglycones) vs. placebo soymilk (no aglycones)	100 mL	Acute, randomized, double-blind, placebo-controlled, crossover	7 healthy postmenopausal women	LC-MS/MS (Targeted)	Serum	• Isoflavones (genistein, daidzein, glycitein, dihydrodaidzein, O-desmethylangolensin, equol)	(103)
Soy	Natto (fermented with *Bacillus natto*)	Natto consumption frequency (questionnaire); 80 g in acute intervention	Cross-sectional, and acute intervention	Group 1: 49 postmenopausal women from Tokyo (8 also participated in acute intervention)<br>Group 2: 25 postmenopausal	HPLC-FLD (Targeted)	Serum	• Vitamin K2 (menaquinone-7)<br>• Phylloquinone (slightly higher in Japanese than British women)<br>• Menaquinone-4 (below limit of detection in most women)	(104)

Summary of Targeted Studies Presenting Candidate FIBs for Fermented Foods								
**Fermented Food**	**All interventions**	**Dose**	**Study Design**	***n***	**Analytical Method**	**Biosample**	**Candidate FIBs[a]**	**Reference**
				women from Hiroshima Group 3: 31 postmenopausal British women				
Soy	Soya milk vs. miso soup	250 mL (soymilk); 31 g Hacco miso; standardized to 48 mg isoflavones	1-day or 2 non-consecutive days, randomized, crossover	21 healthy women	LC-MS (Targeted)	Urine	• Isoflavones (daidzein, genistein, equol)	(105)
Soy	Fermented soy (tempeh) vs. non-fermented soy (soybean)	112 g (tempeh); 125 g (soybean)	9-day, randomized, controlled, crossover	17 healthy men	GC-MS (Targeted)	Urine	• Isoflavones (daidzein, genistein) higher excretion for fermented group (although fermentation decreased isoflavone content of the soy product)	(106)
Soy	Soy flour vs. fermented soybean paste	12.2 g (soy flour); 52 g (soybean paste); standardized to 20 mg isoflavones	Acute, randomized, controlled, crossover	10 healthy volunters	LC-ESI-MS/MS (Targeted)	Urine	• 20 isoflavone metabolites detected, consisting of daidzein and genistein glucuronides and sulfoglucuronides, daidzein sulfate, equol glucuronide, and ODMA glucuronides)<br>• Isoflavones (especially glucuronides of daidzein, genistein) higher in fermented group	(107)
Soy	Natto fermented with *Bacillus subtilis,* double-boiled natto with short interval, vitamin K syrup	50 g/day	3-day intervention with untreated natto; acute, crossover with natto, boiled natto, and vitamin K syrup	32 healthy volunteers	HPLC (detector not specified) (Targeted)	Plasma	• Menaquinone-7 (increased following natto consumption; negligible levels of phylloquinone and menaquinone-4)<br>• Lower levels of menaquinone-7 following boiled natto consumption compared to regular natto and syrup	(108)
Soy	Fermented soybean soup with *Aspergillus oryzae* vs. placebo soup	20 g powder with water; standardized to 24 mg isoflavones/day	4-week, randomized, double-blind, placebo-controlled, parallel	65 healthy postmenopausal women	HPLC-UV (Targeted)	Urine	• Isoflavones (daidzein, genistein, glycitein), total isoflavoens, especially daidzein and glycitein, were higher in fermented group	(109)

Summary of Targeted Studies Presenting Candidate FIBs for Fermented Foods								
**Fermented Food**	**All interventions**	**Dose**	**Study Design**	***n***	**Analytical Method**	**Biosample**	**Candidate FIBs[a]**	**Reference**
Soy	Soybean milk, fried bean curd puff, fresh bean curd, soybeans, fermented bean curd, and other bean foods	Quartiles of soy product consumption, including soybean milk, fried bean curd puff, fresh bean curd, soybeans, fermented bean curd, and other bean foods (from FFQ)	Case-control nested within a large randomized trial of breast self-examination among textile workers in Shanghai	1823 women (1590 women without breast cancer, and 233 women with breast cancer)	LC-coularray and LC-MS (Targeted)	Serum	• Isoflavones (daidzein, genistein)	(110)
Soy	Regular natto vs. reinforced natto 1 vs. reinforced natto 2	50 g (775 ug/100g MK7; 1298 ug/100g MK7; 1765 ug/100g MK7)	7-day crossover	8 healthy men	HPLC (detector not specified) (Targeted)	Serum	• Vitamin K2 (menaquinone-7)	(111)
	Rare intake of natto vs. occasional intake vs. frequent intake	~0 (rare); a few times a month (occasional), a few times a week (frequent) (from food record)	Cross-sectional	134 healthy volunteers				
Soy	Biscuits with fermented soybean meal vs. biscuits with soybean meal	75 g; dose equivalent to 0.44 mg/kg body weight of isoflavones	Acute, randomized, double-blind, crossover	18 healthy volunteers	HPLC-DAD-FLD (Targeted)	Urine	• Glycitein<br>• Daidzein<br>• Genistein<br>• Dihydrodaidzein<br>• O-demethylangolensin<br>• Dihydrogenistein<br>• 6-hydroxy-O-demethylangolensin<br>• Equol<br>• Total aglycones<br>• Total colonic metabolites	(112)
Soy	Testmeals with fermented soybean vs. non-fermented soybean	33 g of soybean	Acute (2-dose), randomized, controlled, crossover	12 healthy volunteers	UPLC-DAD-QTOF-MS (Targeted)	Plasma, urine	• Soy isoflavones: genistein 5-O-glucoside, daidzein 7-O-glucoside (daidzin), glycitein 7-O-glucoside (glycitin), genistein 7-O-glucoside (genistin), daidzein 4′-O-(6″-O-malonyl)glucoside, genistein 5-O-(6″-O-malonyl)glucoside, daidzein 7-O-(4″-O-malonyl)glucoside, glycitein 7-O-(4″-O-malonyl)glucoside, daidzein 7-O-(6″-O-malonyl)glucoside, glycitein 7-O-(6″-O-malonyl)glucoside, daidzein 7-O-(6″-O-succinoyl)glucoside, daidzein 7-O-(6″-O-acetyl)glucoside, genistein 4′-O-(6″-O-malonyl)glucoside, genistein 7-O-(4″-O-malonyl)glucoside, glycitein 7-O-(6″-O-acetyl)glucoside, genistein 7-O-(6″-O-malonyl)glucoside, genistein 4′-O-(6″-O-	(113)

Summary of Targeted Studies Presenting Candidate FIBs for Fermented Foods								
**Fermented Food**	**All interventions**	**Dose**	**Study Design**	***n***	**Analytical Method**	**Biosample**	**Candidate FIBs[a]**	**Reference**
							acetyl)glucoside, daidzein, genistein 7-O-(6″-O-succinoyl)glucoside glycitein, genistein 7-O-(6″-O-acetyl)glucoside genistein, Sum of glucoside forms, simple glucoside, acetyl glucoside, succinyl glucoside, malonyl glucoside)	
Soy	Fermented soybean paste (Cheonggukjang)	50 g/kg body weight	Acute intervention	48 healthy men	UPLC-QTOF-MS (Targeted)	Plasma	• Isoflavone metabolites: Daidzein, Genistein, Glycitein, 3-hydroxydaidzein, 2-hydroxygenistein, Daidzein 4'-glucuronide, Daidzein 7-glucuronide, Daidzein diglucuronide, Daidzein 4'-sulfate, Daidzein 7-sulfate 4'-glucuronide, Genistein 7-glucuronide, Genistein diglucuronide, Genistein-7-glucuronide-4'-sulfate, Genistein 4'-sulfate, Genistein-7-sulfate, Dihydrogenistein, Dihydrodaidzein sulfate, Equol-7-glucuronide, Equol-4-sulfate, 5-hydroxy equol, O-Desmethylangolensin)	(114)
Bread	Whole-grain and fibre-rich rye bread vs. refined wheat bread	174 g/day (wholegrain products); 188 g/day (refined white breads)	12-week, randomized, parallel	51 volunteers with metabolic syndrome	GC-MS (Targeted)	Plasma	• Alkylrecorsinols (AR homologs C17:0-25:0)	(115)
Bread	Wholegrain wheat and rye	Continuous intake of various wholegrains (from FFQ)	Association study	360 postmenopausal women from the Danish Diet, Cancer and Health Study	GC-MS (Targeted)	Plasma	• Alkylrecorsinols (AR homologs C17:0-25:0)	(116)
Bread	High-fiber rye bread	198 g (containing 45 mg ARs) and 21 g butter	Acute intervention	15 healthy volunteers	HPLC-CEAD (Targeted)	Plasma	• Alkylrecorsinol metabolites DHBA and DHPPA	(117)
Bread	Whole grain breads	Total daily whole grain intake (whole grain soft bread + dark crisp bread) (from FFQ)	Association	20 free-living women from the Swedish Mammography Cohort	GC-MS (Targeted)	Plasma, subcutaneous adipose tissue	• Alkylrecorsinols (AR homologs C17:0-25:0)	(118)
Bread	Wholegrain rye porridge vs. refined wheat bread	2 wholegrain rye porridges (40/55 g), 3 rye porridges with different inulin	Acute, randomized, crossover	21 healthy volunteers	NMR (36 plasma metaboites); GC-MS (short chain	Plasma	• Valine, leucine, isoleucine, lysine, phenylalanine, 2-oxoisocaproate<br>• Acetate, butyrate, propionate, acetoacetate, 3-hydroxybutyrate	(119)

Summary of Targeted Studies Presenting Candidate FIBs for Fermented Foods								
**Fermented Food**	**All interventions**	**Dose**	**Study Design**	***n***	**Analytical Method**	**Biosample**	**Candidate FIBs[a]**	**Reference**
		(55 g); refined wheat bread (55 g)			fatty acids) (Targeted)			
Bread	Rye bran bread products vs. control wheat bread products	250 g	6-week, randomized, crossover	18 healthy postmenopausal women	HPLC-DAD (Targeted)	Urine	• Ferulic acid	(120)
Bread	Wheat bread vs. fresh pasta (both enriched with wheat bran)	132 g (bread); 119 g (pasta)	Acute, randomized, crossover	9 healthy men	GC-MS (Targeted)	Plasma	• L-Isoleucine, L-leucine, lactic acid, fructose, xyloe, arabinose, 2,4-dihydroxybutanoic acid, L-phenylalanine, L-proline (treatment X time effect)<br>• 1,2-diglyceride, 1-methylhistidine, urea, sitosterol, glyceric acid, phosphate, fumaric acid (treatment effect)<br>• C14:0, C17:0, glycerol, beta-alanine, L-valine, L-tryptophan, L-tyrosine, inositol, succinic acid, citric acid, L-ornithine, mannose, 2-hydroxypiperidine, iminodiacetic acid, ribulose/xylulose (treatment and time effect)	(121)
Bread	Wholegrain bread vs. bread enriched with aleurone fraction	94 g (wholegrain bread had 87 mg of ferulic acid, while aleurone bread had 43 mg)	Acute, single-blind, randomized, crossover	15 healthy volunteers	U(H)PLC-MS and MS/MS (Targeted)	Urine, plasma	• Ferulic acid metabolites (especially ferulic acid-4'-O-sulfate, dihydroferulic acid-4'-O-sulfate, dihydroferulic acid-O-glucuronide)	(122)
Bread	Wholegrain wheat crisp bread vs. wholegrain rye crisp bread	100 g/day	1-week, randomized, crossover	15 healthy volunteers	GC-MS (ARs), automatic fluoroimmunoassay (enterolactone) (Targeted)	Plasma, erythrocytes, lipoproteins (ARs), serum (enterolactone)	• Alkylrecorsinols (AR homologs C17:0-21:0)<br>• Enterolactone	(123)
Bread	Wholegrain rye and wheat bread vs. no wholegrain rye and wheat products (in habitual diet)	1. Habitual diet of wholegrain rye bread (3-5 pieces/day) and wholegrain wheat bread (2 pieces/day)<br><br>2. Habitual diet and wholegrain rye bread (2 pieces/day) and wholegrain	1. 1 week avoidance, 1 week habitual diet<br><br>2. 2 weeks of habitual diet<br><br>3. 2 weeks of gluten-free diet	1. 4 healthy volunteers<br><br>2. 4 healthy voluntters<br><br>3. 1 volunteer	GC-MS (Targeted)	Plasma, erythrocytes	• Alkylrecorsinols (AR homologs C17:0-25:0)	(124)

Summary of Targeted Studies Presenting Candidate FIBs for Fermented Foods								
**Fermented Food**	**All interventions**	**Dose**	**Study Design**	***n***	**Analytical Method**	**Biosample**	**Candidate FIBs[a]**	**Reference**
		wheat bread (2 pieces/day)<br><br>3. Gluten-free diet with no wheat, rye, or barley products						
Bread	High-fiber rye bread	198 g (containing 100 mg ARs) and 21 g butter	Acute intervention	15 healthy volunteers	HPLC-CEAD (Targeted)	Urine	• Alkylrecorsinol metabolites DHBA and DHPPA	(125)
Bread	Bread, rye bread, refined wheat bread, bran-seed bread	Continuous intake of bread, rye bread, refined wheat bread, bran-seed bread (from 3-day food records)	Association study	31 free-living men with prostate cancer and 91 non-cancer control men	HPLC-CEAD (Targeted)	Plasma (DHPPA), urine (DHBA and DHPPA)	• Alkylrecorsinol metabolites DHBA and DHPPA	(126)
Bread	Wholegrain products	Continuous intake of wholegrain products, including rye bread, wholegrain bread, rolled oats and muesli, and crispbread (from FFQ)	Association study	43 893 volunteers from the Danish Diet, Cancer, and Health cohort (ARs were measured in subset of 516 volunteers)	GC-MS (Targeted)	Plasma	• Alkylrecorsinols (AR homologs C17:0-25:0)	(127)
Bread	Wholegrain wheat vs. refined wheat	70 g/day biscuits (wholegrain); 33 g/day crackers and 27 g/day toasted bread (refined grain)	8-week, randomized, placebo-crontrolled, parallel	80 healthy obese/over weight volunteers	HPLC-MS/MS (Targeted)	Serum, urine, feces	• Phenolic acids (dihydroferulic acid in serum, ferulic acid in feces following wholegrain wheat)	(128)
Bread	Refined-grain wheat bread vs. wholegrain wheat bread	196 g (refined grain); 208 g (wholegrain), both with 21 g butter	Acute, crossover	12 healthy volunteers	LC-MS (Targeted)	Urine	• Alkylrecorsinol metabolites (3,5-DHBA glycine, 3,5-DHPPTA, 3,5-DHBA, 3,5-DHPPA)	(129)

Summary of Targeted Studies Presenting Candidate FIBs for Fermented Foods								
**Fermented Food**	**All interventions**	**Dose**	**Study Design**	***n***	**Analytical Method**	**Biosample**	**Candidate FIBs[a]**	**Reference**
Bread	Wholegrain rye bread vs. low-fiber wheat bread	214 g (rye); 178 (wheat)	8-week, randomized, crossover	39 healthy postmenopausal women	GC-MS (ARs); fluoroimmunoassay (enterolactone) (Targeted)	Plasma	• Alkylrecorsinols<br>• Enterolactone	(130)
Bread	White wheat flour soft bread and white wheat flour crispbread vs. rye bran-enriched soft bread and whole grain rye crisp bread	142.8 g/day and 92.4 g/day; 180.6 g/day and 91.0 g/day	2-week, randomized, crossover	10 volunteers with previous proctocolectomy for ulcerative colitis	GC-MS (Targeted)	Ileal effluent	• Alkylrecorsinols (AR homologs C17:0-25:0)	(131)
Bread	Sourdough fermented bread vs. traditional sourdough bread vs. baker's yeast bread	215 mL (mimicking chewing and homogenisation of the bread portion + 160 mL water)	Acute, randomized, double-blind, crossover	36 healthy volunteers	Biochrom 30 series Amino Acid Analyzer (Targeted)	Plasma	• Total free amino acids	(132)
Bread	Rye-based bread vs. white wheat bread	75 g	3-day, randomized, controlled, crossover	38 healthy volunteers	GC (detector not specified) (Targeted)	Plasma	• Acetate<br>• Butyrate<br>• Total SCFAs	(133)
Cocoa	Sugar-free, flavanol-rich cocoa alone vs. low-dose sugar + cocoa vs. high-dose sugar + cocoa	0.125 g/kg body weight (cocoa); 8.75 kJ/kg (low-dose sugar); 17.5 kJ/kg (high-dose sugar)	Acute crossover	6 healthy volunteers	HPLC-coulometric multiple-array detection (Targeted)	Plasma	• Total flavonols (epicatechin + catechin)	(134)
	Sugar-free, flavanol-rich cocoa alone vs. bread+ cocoa vs. steak + cocoa vs. butter + cocoa (macronutrient-rich foods)	0.125 g/kg body weight (cocoa); 17.5 kJ/kg (foods)					• Total flavonols (epicatechin + catechin)	

Summary of Targeted Studies Presenting Candidate FIBs for Fermented Foods								
**Fermented Food**	**All interventions**	**Dose**	**Study Design**	***n***	**Analytical Method**	**Biosample**	**Candidate FIBs[a]**	**Reference**
	Sugar-free, flavanol-rich cocoa alone vs. bread + cocoa vs. milk + cocoa vs. grapefruit juice + cocoa	0.125 g/kg body weight (cocoa); 17.5 kJ/kg (foods)					• Total flavonols (epicatechin + catechin)	
	Water + cocoa vs. water + cocoa + Famotidine	0.125 g/kg body weight (cocoa); 20 mg (Famotidine)					• No difference in flavonols	
Cocoa	Semi-sweet chocolate (M&Ms chocolate mink baking bits) vs. vanilla milk chips	80 g	Acute, parallel	13 healthy volunteers	HPLC-Coulochem II detector (Targeted)	Plasma	• Catechin<br>• Epicatechin	(135)
Cocoa	Nestle Noir 70% dark chocolate	100 g	Acute intervention	5 healthy volunteers	LC-MS/MS (Targeted)	Plasma, urine	• (-)-Epicatechin-3'-beta-D-glucuronide<br>• (-)-Epicatechin-3'-sulfate<br>• 3'-O-methyl epicatechin sulfates	(136)
Cocoa	Cocoa powder + skimmed milk vs. skimmed milk	20 g/day with 250 mL skimmed milk; 500 mL/day skim milk	4-week, randomized, controlled, crossover	42 healthy volunteers	LC-MS/MS (Targeted)	Plasma, urine	• (-)-Epicatechin<br>• (-)-Epicatechin glucuronides and sulfates<br>• O-methyl-epicatechin glucuronides and sulfates<br>• DHPV glucuronides and sulfates<br>• MHPV glucuronides and sulfates<br>• Vanillic acid<br>• 3,4-Dihydroxyphenylacetic acid<br>• 3-Hydroxyphenylacetic acid	(137)
Cocoa	Cocoa powder + water vs. cocoa powder + whole milk vs. whole milk	40 g (cocoa powder) in 250 mL (milk or water)	Acute, randomized, crossover	21 healthy volunteers	HPLC-MS/MS (Targeted)	Urine	• (-)-Epicatechin glucuronide<br>• (-)-Epicatechin sulfates	(138)
Cocoa	Dark chocolate vs. high sucrose chocolate vs. high milk protein chocolate vs. sucrose milk protein	40 g or 250 mL (for drinks)	Acute, randomized, crossover	6 healthy volunteers	HPLC-EAD (Targeted)	Serum	• Epicatechin, catechin, and glucuronide and sulfate conjugates	(139)

Summary of Targeted Studies Presenting Candidate FIBs for Fermented Foods								
**Fermented Food**	**All interventions**	**Dose**	**Study Design**	***n***	**Analytical Method**	**Biosample**	**Candidate FIBs[a]**	**Reference**
	chocolate drink vs. non-nutritive sweetner milk protein chocolate drink							
Cocoa	Flavonoid-rich dark chocolate (72%) vs. flavonoid-free placebo (dyed) white chocolate	50 g	Acute, randomized, controlled, parallel	65 healthy men	HPLC-EAD (Targeted)	Plasma	• Epicatechin	(140)
Cocoa	High-flavanol chocolate with mannitol vs. high-flavanol chocolate with sucrose vs. low-flavanol chocolate with sucrose	25 g; standardized to macro and micronutrients, theobromine, and caffeine	Acute, randomized, double-blind, crossover	15 healthy volunteers	HPLC-FLD/UV (Targeted)	Plasma	• Total cocoa flavanols (epicatechin, catechin, procyanidin oligomers)<br>• 3' and 4'-O-methylated epicatechins	(141)
Cocoa	Cocoa beverage vs. milk	40 g cocoa powder in 250 mL milk; 250 mL milk	Acute, randomized, controlled, crossover	36 healthy volunteers	Folin-Ciocalteu Assay (modified) (Targeted)	Urine	• Total polyphenols (+-catecgub equivalents)	(142)
Cocoa	Cocoa with water	40 g cocoa powder in 250 mL water	Acute intervention	21 healthy volunteers	LC-MS/MS (Targeted)	Urine	• Caffeic acid<br>• Ferulic acid<br>• 3-hydroxyphenylacetic acid<br>• Vanillic acid<br>• 3-hydroxybenzoic acid<br>• 4-hydroxyhippuric acid<br>• Hippuric acid<br>• (-)-Epicatechin<br>• Procyanidin B2	(143)
Cocoa	Dark chocolate (70%) vs. white chocolate	45 g	2-week, controlled, parallel	20 healthy volunteers	HPLC-DAD (Targeted)	Plasma	• (-)-Epicatechin	(144)
Cocoa	Cocoa beverage with milk vs. cocoa beverage with water	10 g cocoa power in 250 mL milk or water	Acute crossover	9 healthy volunteers	HPLC-PDA, HPLC-MS (Targeted)	Plasma, urine	• (Epi)catechin-O-sulfate<br>• (-)-Epicatechin-O-glucuronide<br>• (Epi)catechin-O-sulfate<br>• O-Methyl-(epi)catechin-O-sulfate	(145)

Summary of Targeted Studies Presenting Candidate FIBs for Fermented Foods								
**Fermented Food**	**All interventions**	**Dose**	**Study Design**	***n***	**Analytical Method**	**Biosample**	**Candidate FIBs[a]**	**Reference**
Cocoa	Hershey's milk chocolate	113 g	Acute intervention	6 nursing mothers	HPLC (detector not specified) (Targeted)	Plasma, saliva, breast milk	• Theobromine	(146)
Cocoa	Dark chocolate vs. white chocolate	6.3 g/day	18-week, randomized, controlled, parallel	44 healthy volunteers	LC-MS/MS (Targeted)	Plasma	• Epicatechin<br>• Catechin<br>• Procyanidin B2<br>• Procyanidin B2 galate	(147)
Cocoa	Cocoa powder with no added methylxanthine vs. cocoa powder enriched with methylxanthine	15 g dissolved in 200 mL (milk)	Acute, randomized, controlled, crossover	13 healthy volunteers	HPLC-DAD, LC-QTOF, LC-DAD (Targeted)	Plasma, urine	• Theobromine<br>• Caffeine<br>• Paraxanthine<br>• Theophylline<br>• Methylxanthines (1-, 3-, 7, 1,7-, 3,7-, 1,3-, 1,3,7-)<br>• Methyluric acid (1-, 1,3-, 1,7-, 3,7-, 1,3,7-)	(148)
Cocoa	Cocoa in skimmed milk vs. skimmed milk	40 g/day (cocoa powder) in 500 mL (milk)	4-week, randomized, crossover	42 volunteers with high CV risk	LC-MS/MS (Targeted)	Urine	• (-)-Epicatechin glucuronides and sulfates<br>• O-Methyl-epicatechin glucuronides and sulfates<br>• 5-(3-Methyoxy-4-hydroxyphenyl)-gamma-valerolactone glucuronides and sulfates<br>• 5-(3,4-Methyoxy-4-hydroxyphenyl)-gamma-valerolactone<br>• 3-Hydroxyphenylacetic acid<br>• 3,4-Dihydroxyphenylacetic acid<br>• Vanillic acid	(149)
Cocoa	Dark chocolate (85%) vs. milk chocolate (<35%)	40 g	Acute, single-blind, crossover	20 healthy volunteers and 20 smokers	HPLC-UV (Targeted)	Serum	• Epicatechin	(150)
Cocoa	Cocoa beverage	0.375 g cocoa/kg body weight in 300 mL water	Acute intervention	5 healthy volunteers	HPLC-CEAD, LC-MS/MS (Targeted)	Plasma	• Procyanidin dimer B2 [epicatechin-4beta-8)-epicatechin]<br>• Catechin<br>• Epicatechin	(151)
Cocoa	Flavanoid-enriched chocolate bars vs. placebo	27 g/day with 90 mg catechin/day	1-year, randomized, controlled, parallel	93 postmenopausal women with type 2 diabetes	LC-MS/MS (Targeted)	Urine	• Epicatechin monoglucuronide<br>• Methylepicatechin monoglucuronide<br>• Epicatechin monosulfate<br>• Methylepicatechin sulfate<br>• Epicatechin monosulfate monoglucuronide	(152)
Cocoa	Cocoa in whole milk vs. cocoa in water vs. milk	40 g (cocoa powder) in 250 mL (milk or water)	Acute, randomized, crossover	21 healthy volunteers	LC-MS/MS (Targeted)	Plasma	• (-)-Epicatechin glucuronide	(153)

Summary of Targeted Studies Presenting Candidate FIBs for Fermented Foods								
**Fermented Food**	**All interventions**	**Dose**	**Study Design**	***n***	**Analytical Method**	**Biosample**	**Candidate FIBs[a]**	**Reference**
Cocoa	Cocoa beverage	Powder in 500 mL water; 10.7 mg cocoa flavanols/kg body weight	Acute intervention (X2)	7 healthy young volunteers	HPLC-FLD/UV/ECD (Targeted)	Plasma, urine	• (-)-Epicatechin-3'-beta-D-glucuronide<br>• (-)-Epicatechin-3'-sulfate<br>• 3'-O-Methyl-(-)-epicatechin-5-sulfate<br>• 3'-O-Methyl-(-)-epicatechin-7-sulfate	(154)
	Cocoa beverage vs. acetaminophen in beverage	Powder in 500 mL water; 5.3 and 10.7 mg cocoa flavanols/kg body weight; 2X 500 mg acetaminophen in water	Acute, randomized, crossover	20 young healthy men and 20 elderly healthy men			• (-)-Epicatechin-3'-beta-D-glucuronide<br>• (-)-Epicatechin-3'-sulfate<br>• 3'-O-Methyl-(-)-epicatechin-5-sulfate<br>• 3'-O-Methyl-(-)-epicatechin-7-sulfate	
Cocoa	High-flavanol cocoa drink vs. low-flavanol cocoa drink	Powder in 300 mL water; 917 mg or 37 mg cocoa flavanols	Acute, randomized, double-blind, crossover	10 healthy men	HPLC-MS (Targeted)	Plasma	• (-)-Epicatechin<br>• Catechin<br>• 4'-Methyl-epicatechin<br>• Epicatechin-O-beta-D-glucuronide<br>• 4'-O-Methyl-epicatechin-O-beta-D-glucuronide (highest)	(155)
Cocoa	Dark chocolate	100 g	Acute intervention	Healthy volunteers (number not reported)	LC-MS/MS (Targeted)	Urine	• 3'-O-methyl-(-)-epicatechin-5/7-O-sulfate<br>• 4'-O-methyl-(-)-epicatechin-5/7-O-sulfate	(156)
Cocoa	High-flavanol chcolate vs. low-flavanol chocolate	40 g (first visit single dose); 20 g/day thereafter	Acute and 12-week, randomized, parallel	44 healthy pregnant women	HPLC-FLD (Targeted)	Plasma	• Epicatechin<br>• Catechin<br>• Caffeine<br>• Theobromine<br>• Theophylline	(157)
Cocoa	Cocoa in milk vs. whole milk	40 g (cocoa powder) in 250 mL (milk)	Acute, randomized, crossover	5 healthy volunteers	LC-MS/MS (Targeted)	Plasma, urine	• (-)-Epicatechin<br>• (-)-Epicatechin glucuronide<br>• (-)-Epicatechin sulfate	(158)
Cocoa	Alkalized cocoa powder in water	20.3 g (cocoa powder) in 400 mL water	Acute intervention	8 healthy volunteers	LC-MS/MS (Targeted)	Urine	• N-[4'-hydroxy-(E)-cinnamoyl]-L-aspartic acid<br>• N-[4'-hydroxy-(E)-cinnamoyl]-L-glutamic acid<br>• N-[4'-hydroxy-3'-methoxy-(E)-cinnamoyl]-L-tyrosine	(159)
Cocoa	Chocolate	80 g	Acute intervention	11 healthy volunteers	GC-MS, HPLC-ESI-MS/MS (Targeted)	Urine	• M-Hydroxyphenylpropionic acid<br>• Ferulic acid<br>• 3,4-dihydroxyphenylacetic acid<br>• M-hydroxyphenylacetic acid<br>• Vanillic acid<br>• M-Hydroxybenzoic acid	(160)

Summary of Targeted Studies Presenting Candidate FIBs for Fermented Foods								
**Fermented Food**	**All interventions**	**Dose**	**Study Design**	***n***	**Analytical Method**	**Biosample**	**Candidate FIBs[a]**	**Reference**
Cocoa	Cocoa in whole milk vs. cocoa in water	40 g (cocoa powder) in 250 mL (milk or water)	Acute, randomized, crossover	21 healthy volunteers	LC-MS/MS (Targeted)	Urine	• 3,4-dihydroxyphenylacetic acid<br>• Phenylacetic acid<br>• Protocatechuic acid<br>• 4-hydroxybenzoic acid<br>• 4-hydroxyhippuric acid<br>• Hippuric acid<br>• Vanillic acid<br>• Caffeic acid<br>• Ferulic acid	(161)
Cocoa	Cocoa beverage vs. whole milk	40 g (cocoa powder) in 250 mL water; 250 mL (milk)	Acute, randomized, crossover	21 healthy volunteers	LC-MS/MS (Targeted)	Urine	• (-)-Epicatechin glucuronide<br>• (-)-Epicatechin sulfates	(162)
Cocoa	Chocolate (M&Ms semi-sweet chocolate mink baking bits) at 4 doses	0, 27, 53, 80 g	Acute, randomized, crossover	20 healthy volunteers	LC-Coulochem II coulometric detector (Targeted)	Plasma	• (-)-Epicatechin	(163)
Cocoa	Chocolate vs. cocoa	35 g cocoa powder in each	Acute crossover	5 healthy men	HPLC-MS (Targeted)	Plasma, urine	• (-)-Epicatechin and metabolites (glucuronides, sulfates, sulfoglucuronides, non-methylated, methylated)	(164)
Cocoa	Cocoa drink with low flavanols, medium flavanols, vs. high flavanols	18 g cocoa beverage mix in 250 mL water	Acute, randomized, double-blind, controlled, crossover	10 diabetic patients	HPLC-FLD (Targeted)	Plasma	• Total flavanols (epicatechin, catechin, and methylated, glucuronidated)	(165)
	Cocoa drink with high flavanols vs. low flavanols		30-day, randomized, double-blind, controlled, parallel	40 diabetic patients				
Cocoa	High-flavanol dark chocolate vs. low-flavanol dark chocolate	46 g	2-week, randomized, double-blind, placebo-controlled, parallel	21 healthy volunteers	HPLC-ECD (Targeted)	Plasma	• Epicatechin	(166)
Cocoa	Dark chocolate (85%) vs. milk chocolate (≤35%)	40 g	Acute, randomized, single-blind, crossover	20 patients with peripheral	HPLC-UV (Targeted)	Serum	• Epicatechin<br>• Epicatechin-3-O-methylether<br>• Epigallocatechin-3-gallate	(167)

Summary of Targeted Studies Presenting Candidate FIBs for Fermented Foods								
**Fermented Food**	**All interventions**	**Dose**	**Study Design**	***n***	**Analytical Method**	**Biosample**	**Candidate FIBs[a]**	**Reference**
				artery disease				
Cocoa	Chocolate vs. cola vs. caffeine capsules vs. theobromine capsules	82 g (chocolate); 800 mL (cola); 72 mg (caffeine capsules); 370 mg (theobromine capsule)	Acute crossover	7 healthy volunteers	HPLC-DAD (Targeted)	Plasma	• Methylxanthine<br>• Caffeine<br>• Paraxanthine<br>• Theophylline<br>• Theobromine	(168)
Cocoa	Cocoa drink vs. sugar	36 g cocoa powder	2-week, randomized, controlled, parallel	15 healthy men	HPLC-amperometric ECD (Targeted)	Plasma, urine	• Epicatechin	(169)
Cocoa	Nestle Noir dark chocolate low-dose vs. high-dose	40 g; 80 g	Acute crossover	8 healthy men	HPLC-DAD/FLD (Targeted)	Plasma	• Theobromine<br>• Epicatechin	(170)
Cocoa	Dark chocolate vs. milk chocolate	200 g	3-day, randomized, controlled, parallel	58 healthy volunteers	Enzymatic method (oxalate)	Urine	• Oxalate	(171)
Cocoa	Dark chocolate (70%) vs. white chocolate (4% cocoa)	1 g/kg body weight	Acute crossover	10 healthy volunteers	Not reported	Plasma	• Catechin<br>• Epicatechin and metabolites	(172)
Cocoa	Dark chocolate vs. theobromine sodium acetate oral solution	6 mg/kg body weight of theobromine (chocolate); 10 mg/kg body weight theobromine (oral solution)	7-day intervention (chocolate); acute intervention (oral solution)	12 healthy volunteers	HPLC-solit scintillator flow cell detector (Targeted)	Urine, serum	• Theobromine	(173)
Cocoa	Milk chocolate vs. chocolate powder vs. dark chocolate	40 g/day	7-day crossover	20 healthy volunteers	HPLC-PDA (Targeted)	Urine	• Theobromine	(174)
Cocoa	Non-alkalized cocoa beverage vs. alkalized cocoa beverage	0.6 g/kg body weight (alkalized powder); 0.8 g/kg body	Acute, randomized, double-blind, crossover	12 healthy volunteers	HPLC-CEAD (Targeted)	Plasma	• Epicatechin	(175)

Summary of Targeted Studies Presenting Candidate FIBs for Fermented Foods								
**Fermented Food**	**All interventions**	**Dose**	**Study Design**	***n***	**Analytical Method**	**Biosample**	**Candidate FIBs[a]**	**Reference**
		weight (non-alkalized powder) in 6 mL/kg body weight skimmed milk						
Cocoa	Conventional cocoa beverage vs. flavanol-rich cocoa beverage	15 g (conventional); 25 g (flavanol rich) in 200 mL semi-skimmed milk	Acute, radomised, blind, crossover	13 healthy volunteers	HPLC-ESI-QTOF-MS (Targeted)	Plasma, urine	• Epicatechin metabolites (Epicatechin-3-glucuronide, 3-methoxy-glucuronide, 3-sulfate, methoxy-sulfate)<br>• Phenyl-gamma-valerolactone derivatives (DHPV lactones, 5-(3-Hydroxyphenyl)-γ-valerolactone-4-glucuronide, 5-(4-Hydroxyphenyl)-γ-valerolactone-3-glucuronide, 5-(Hydroxyphenyl)-γ-valerolactone-sulfate, 5-Phenyl-γ-valerolactone-methoxy-glucuronide, 5-Phenyl-γ-valerolactone-methoxy-sulfate, 5-(3-Hydroxyphenyl)-γ-valerolactone, 5-Phenyl-γ-valerolactone-3-glucuronide, 5-Phenyl-γ-valerolactone-3-sulfate)<br>• Phenylvaleric acid derivatives (4-Hydroxy-5-(3,4-dihydroxyphenyl)valeric acid, 4-Hydroxy-5-(hydroxyphenyl)valeric acid-glucuronide, 4-Hydroxy-5-(hydroxyphenyl)valeric acid-sulfate)<br>• Other microbial metabolites (3,4-Dihydroxyphenylpropionic acid, 3-Methoxy-4-hydroxyphenylpropionic acid 3-Hydroxyphenylpropionic acid 3,4-Dihydroxyphenylacetic acid, 3-Methoxy-4-hydroxyphenylacetic acid 3-Hydroxyphenylacetic acid, Ferulic acid Isoferulic acid, 3,4-Dihydroxybenzoic acid, 4-Hydroxyhippuric acid, 3-Hydroxyhippuric acid, Hydroxybenzoic acid)	(176)
Cocoa	Milk chocolate vs. hazelnut and cocoa spread	60 g	Acute, randomized, single-blind, crossover	20 healthy smokers	Folin-Ciocalteau method (polyphenols); GC-MS (vitamin E) (Targeted)	Plasma (polyphenols); serum (vitamin E)	• Total polyphenols<br>• Vitamin E	(177)
Cocoa	Dark chocolate (90%)	50 g	Acute intervention	18 healthy men	U(H)PLC-ESI-MS/MS (Targeted)	Plasma	• Epicatechin and metabolites (glucuronides, glucuronide-sulfate, sulfates, methyl-epicatechin sulfates)<br>• Phenyl-gamma-valerolactones (5-(3-Hydroxyphenyl)-g-valerolactone-4-glucuronide, 5-Phenyl-g-valerolactone-glucuronide-sulfate, 5-(4-Hydroxyphenyl)-g-valerolactone-3-glucuronide, 5-Phenyl-g-valerolactone-3-glucuronide, 5-(Hydroxyphenyl)-g-valerolactone-sulfate isomers, 5-Phenyl-g-valerolactone-sulfate-methoxy, 5-Phenyl-g-valerolactone-3-sulfate)	(178)

Summary of Targeted Studies Presenting Candidate FIBs for Fermented Foods								
**Fermented Food**	**All interventions**	**Dose**	**Study Design**	***n***	**Analytical Method**	**Biosample**	**Candidate FIBs[a]**	**Reference**
Cocoa	Cocoa-enriched dark chocolate (70%) vs. cocoa-depleted control chocolate	561 kcal	Acute, single-blind, randomized, crossover	16 healthy male cyclists	RP-HPLC-UV (Targeted)	Plasma	• Epicatechin<br>• Theobromine	(179)
Coffee	Coffee consumption	0, 1, 2-4, 5-8, >8 cups of coffee/day (from questionnaire)	Association study	3503 patients undergoing elective coronary angiograph y from the WECAC cohort	LC-MS/MS (Targeted)	Plasma	• Trigonelline<br>• Nicotinamide<br>• N-methylnicotinamide	(180)
Coffee	Instant coffee (Nescafe Gold Blend)	3.4 g powder in 200 mL water	Acute intervention	5 volunteers with an ileostomy	HPLC-PDA-MS/MS (Targeted)	Ileal effluent, urine	• Total chlorogenic acid and metabolites<br>• 3-, 4-, and 5-O-caffeoylquinic acids<br>• 3-, 4-, and 5-O-caffeoylquinic acid sulfates<br>• 3- and 4--O-caffeoylquinic acid glucuronides<br>• 3-, 4-, and 5-O-feruloylquinic acids<br>• 3-, 4-, and 5-O-feruloylquinic acid sulfate<br>• 3- and 4-O-feruloylquinic acid glucuronides<br>• 3- and 4-O-caffeoylquinic acid lactones<br>• 3- and 4-O-caffeoylquinic glucuronides and sulfates<br>• 4- and 5-O-p-coumaroylquinic acid<br>• 3,4-, 3,5-, and 4,5-O-di-caffeoylquinic acid<br>• Caffeic acid<br>• Caffeic acid 4- and 3-O-sulfates<br>• Ferulic acid<br>• Ferulic acid-4-O-sulfate<br>• Feruloylglycine<br>• Dihydroferulic acid-4-O-sulfate<br>• Isoferulic acid 3-O-sulfate and glucuronide<br>• Dihydrocaffeic acid-3-O-sulfate	(181)
Coffee	Instant coffee	200 mL	Acute intervention	11 healthy volunteers	HPLC-PDA-MS/MS (Targeted)	Plasma, urine	• Dihydrocaffeic acid 3’-sulfate<br>• Dihydrocaffeic acid-3’-O-glucuronide<br>• Caffeic acid 4’-sulfate<br>• Dihydroferulic acid 4’-sulfate<br>• Caffeic acid 3’-sulfate<br>• Dihydroferulic acid 4’-O-glucuronide<br>• Ferulic acid 4’-sulfate<br>• Isoferulic acid 3’-sulfate<br>• Dihydroisoferulic acid 3’-O-glucuronide	(182)

Summary of Targeted Studies Presenting Candidate FIBs for Fermented Foods								
**Fermented Food**	**All interventions**	**Dose**	**Study Design**	***n***	**Analytical Method**	**Biosample**	**Candidate FIBs[a]**	**Reference**
							• Isoferulic acid 3'-O-glucuronide	
Coffee	Coffee vs. tea vs. Cola soft drink	At least 1 cup of coffee or tea, 500 mL soft drink	Acute intervention	146 healthy volunteers	HPLC-UV (Targeted)	Urine	• 5-acetylamino-6-formylamino-3-methyluracil<br>• 1-methylxanthine	(183)
Coffee	Low-polyphenol coffee vs. high-polyphenol coffee vs. caffeine in hot water	3.6 g ground coffee to 50 mL water; 110 mg caffeine	Acute, randomized, controlled, crossover	15 healthy men	U(H)PLC-ESI-MS/MS (Targeted)	Plasma	• Total chlorogenic acid metabolites<br>• 3-caffeoylquinic acid<br>• 4-caffeoylquinic acid<br>• Caffeic-4-O-sulfate<br>• 3-feroylquinic acid<br>• 4-feroylquinic acid<br>• 5-feroylquinic acid<br>• Ferulic acid<br>• Isoferulic acid<br>• Methylferulic acid<br>• Ferulic-4-O-glucuronide<br>• Isoferulic-3-O-glucuronide<br>• Ferulic-4-O-sulfate<br>• Isoferulic-3-O-sulfate	(184)
Coffee	Caffeinated coffee vs. decaffeinated coffee vs. combination	2 cups (caffeinated); 2 cups (decaffeinated); 1 cup (caffeinated) + 1 cup (decaffeinated)	Acute, randomized, crossover	17 men with coronary artery disease performing a treadmill test	HPLC (detector not specified) (Targeted)	Serum	• Caffeine	(185)
Coffee	Coffee (mostly instant coffee) and tea	Number of cups of coffee and tea consumption (FFQ for usual consumers; 24h recall for current consumers)	Association study	111 free-living volunteers (usual consumers); 344 healthy volunteers (current consumers)	GC-MS (Targeted)	Urine	• Isoferulic acid	(186)

Summary of Targeted Studies Presenting Candidate FIBs for Fermented Foods								
**Fermented Food**	**All interventions**	**Dose**	**Study Design**	***n***	**Analytical Method**	**Biosample**	**Candidate FIBs[a]**	**Reference**
Coffee	Filtered coffee	0 (refrain from consumption), 4 cups/day, 8 cups/day (150 mL/cup)	1-month, crossover	47 habitual coffee drinkers	HPLC-MS, GC-MS (Targeted)	Serum, plasma	• Caffeine<br>• Paraxanthine<br>• Theobromine<br>• Theophylline<br>• Caffeic acid<br>• Dihydrocaffeic acid<br>• Ferulic acid<br>• Isoferulic acid<br>• Dihydroferulic acid<br>• Dihydroisoferulic acid<br>• 3-(3-Hydroxyphenyl)propanoic acid<br>• 3-(3,4-Dimethoxyphenyl)propanoic acid<br>• 3,4-Dimethylcaffeic acid<br>• 3-Coumaric acid	(187)
Coffee	Coffee	Cups of coffee consumption (1-2, 3-7, >8) (from survey)	Association study	284 healthy men	LC-ESI-MS/MS (Targeted 363 metabolites)	Serum	• Sphingomyelin SM(OH,COOH)<br>• Sphingomyelin SM(OH)<br>• Long- and medium-chain acylcarnitines	(188)
Coffee	Coffee	Non-coffee consumers (0 mL/day), low-coffee consumers (≤100 mL/day), high-coffee consumers (>100 mL/day) (from 24h recalls and FFQ)	Association study	169 healthy volunteers	FIA-MS/MS, HPLC-MS/MS (Targeted)	Plasma	• 3-, 4-, 5-Caffeoylquinic acid<br>• 5-Feruloylquinic acid<br>• 4-Ethylguaiacol<br>• 4-Vinylguaiacol<br>• Catechol<br>• Pyrogallol<br>• LysoPC C16:0/16:1<br>• LysoPC C18:0/18:1	(189)
Coffee	Coffee and tea	Coffee and tea consumption (from FFQ and 3-day food record)	Association study	57 healthy women	HPLC-MS/MS (Targeted)	Plasma, urine	• Caffeic acid<br>• Chlorogenic acid	(190)
Coffee	Coffee	Preparation: 48 g coffee powder in 900 mL water; 350 mL consumed	Acute intervention	13 healthy volunteers	HILIC-MS/MS (Targeted)	Plasma	• Trigonelline<br>• N-methylpyridinium	(191)
Coffee	Coffee vs. tea vs. water	Dose not specified	Acute intervention	13 healthy volunteers	CE-ESI-TOF-MS, HPLC-	Urine	• Chlorogenic acid<br>• Caffeic acid<br>• Coumaric acid	(192)

Summary of Targeted Studies Presenting Candidate FIBs for Fermented Foods								
**Fermented Food**	**All interventions**	**Dose**	**Study Design**	***n***	**Analytical Method**	**Biosample**	**Candidate FIBs[a]**	**Reference**
					MS/MS (Targeted)			
Coffee	Coffee	Preparation: 40 g coffee powder in 250 mL water' 190 mL consumed	Acute intervention	6 healthy volunteers	HPLC-UV, LC-MS (Targeted)	Plasma, urine	• 3-, 4-, and 5-Caffeoylquinic acid (plasma)<br>• 3,4-, 3,5-, and 4,5-Dicaffeoylquinic acid (plasma)<br>• Feryloylquinic acid (plasma, only 1 volunteer)<br>• Dihydrocaffeic acid (urine)<br>• Gallic acid (urine)<br>• Isoferulic acid (urine)<br>• Ferulic acid (urine)<br>• Vanillic acid (urine)<br>• Caffeic acid (urine)<br>• 5-caffeoylquinic acid (urine)<br>• Sinapic acid (urine)<br>• P-hydroxybenzoic acid (urine)<br>• P-coumaric acid (urine)	(193)
Coffee	Instant coffee with low chlorogenic acids vs. medium chlorogenic acids vs. high chlorogenic acids	3.4g coffee powder in 200 mL water	Acute, randomized, double-blind, controlled, crossover	11 healthy volunteers	HPLC-PDA-MS/MS (Targeted)	Plasma, urine	• 3-Caffeoylquinic acid-O-sulfate<br>• Dihydrocaffeic acid-3-O-sulfate<br>• Dihydrocaffeic acid-3-O-glucuronide<br>• 4-caffeoylquinic acid-O-sulfate<br>• Caffeic acid-4-O-sulfate<br>• Dihydroferulic acid-4-O-sulfate<br>• Caffeic acid-3-O-sulfate<br>• Dihydrocaffeic acid<br>• Dihydroferulic acid-4-O-glucuronide<br>• Ferulic acid-4-O-sulfate<br>• 5-caffeoylquinic acid<br>• 3-feruloylquinic acid<br>• Isoferulic acid-3-O-sulfate<br>• Dihydroisoferulic acid-3-O-glucuronide<br>• Isoferulic acid-3-O-glucuronide<br>• Feruloylglycine<br>• 3-Caffeoylquinic acid lactone-O-sulfate<br>• 4-Caffeoylquinic acid lactone-O-sulfate<br>• 4-Feruloylquinic acid<br>• Dihydroferulic acid<br>• 5-Feruloylquinic acid	(194)
Coffee	Coffee at 3 doses	2, 4, and 8 g coffee powder in 400 mL water	Acute, randomized, crossover	10 healthy volunteers	LC-ESI-MS/MS (Targeted)	Plasma	• 3-Feruloylquinic acid<br>• 4-Feruloylquinic acid<br>• 5-Feruloylquinic acid<br>• 3-Caffeoylquinic acid<br>• 4-Caffeoylquinic acid<br>• 5-Caffeoylquinic acid	(195)

Summary of Targeted Studies Presenting Candidate FIBs for Fermented Foods								
**Fermented Food**	**All interventions**	**Dose**	**Study Design**	***n***	**Analytical Method**	**Biosample**	**Candidate FIBs[a]**	**Reference**
							• Ferulic acid<br>• Caffeic acid<br>• Isoferulic acid<br>• Dihydroferulic acid<br>• Dihydrocaffeic acid	
Coffee	Coffee vs. no coffee	1 cup (acute); daily consumption of coffee	Acute intervention	Healthy volunteers (number not specified)	HPLC-UV (Targeted)	Urine	• N-methyl-2-pyridone-5-carboxamide<br>• N-methyl-4-pyridone-5-carboxamide	(196)
Coffee	Hot coffee consumed slowly vs. cold coffee consumed slowly vs. cold coffee consumed fast vs. sugar-free energy drink consumed fast vs. sugar-free energy drink consumed slowly	4.1 g coffee powder in 450 mL water; 450 mL energy drink	Acute, randomized, crossover	24 healthy volunteers	LC-MS (Targeted)	Plasma	• Caffeine	(197)
Coffee	Coffee vs. soy beverage vs. coffee + soy beverage	4 g coffee powder and/or 20 g soymilk powder in 200 mL water	Acute, randomized, crossover	6 healthy volunteers	HPLC-DAD, LC-MS (Targeted)	Urine	• 3-Caffeoylquinic acid<br>• 4-Caffeoylquinic acid<br>• 5-Caffeoylquinic acid<br>• 3,4-Dicaffeoylquinic acid<br>• 3,5-Dicaffeoylquinic acid<br>• 4,5-Dicaffeoylquinic acid<br>• Ferulic acid<br>• Caffeic acid<br>• Isoferulic acid<br>• Dihydrocaffeic acid<br>• Gallic acid<br>• Vanillic acid<br>• Benzoic acid<br>• P-Hydroxybenzoic acid<br>• Siringic acid<br>• Sinapic acid<br>• 3,4-Dihydroxyphenilacetic acid<br>• Hippuric acid<br>• Trans-3-hydroxycinnamic acid	(198)

Summary of Targeted Studies Presenting Candidate FIBs for Fermented Foods								
**Fermented Food**	**All interventions**	**Dose**	**Study Design**	***n***	**Analytical Method**	**Biosample**	**Candidate FIBs[a]**	**Reference**
							• 3-(4-Hydroxyphenyl) propanoic acid<br>• 2,4-Dihydroxybenzoic acid	
Coffee	Instant coffee	1 cup containing 4 mg caffeine/kg body weight	Acute intervention	6 healthy volunteers	HPLC-MS/MS (Targeted)	Urine	• AAMU/AFMU<br>• 1-Methyluric acid<br>• 1-Methylxanthine<br>• 1,3-Dimethyluric acid<br>• 1,7-Dimethyluric acid<br>• 1,3,7-Trimethyluric acid<br>• 3,7-Dimethylxanthine<br>• Paraxanthine<br>• 1,3-Dimethylxanthine<br>• 1,3,7-Trimethylxanthine	(199)
Coffee	Instant coffee	400 mL	Acute intervention	8 healthy volunteers	HPLC-MS/MS (Targeted)	Plasma	• 3,4-Dimethoxycinnamic acid	(200)
Coffee	Instant coffee	4 g in 400 mL water	Acute intervention	Healthy volunteers (number not specified)	LC-MS/MS (Targeted)	Plasma	• Caffeic acid<br>• Dihydrocaffeic acid<br>• Ferulic acid<br>• Dihydroferulic acid<br>• Isoferulic acid	(201)
Coffee	Instant coffee	3.4 g in 200 mL	Acute intervention	11 healthy volunteers	HPLC-PDA-MS/MS (Targeted)	Plasma, urine	• 3-O-Caffeoylquinic acid-O-sulfate<br>• DIhydrocaffeic acid-3-O-sulfate<br>• DIhydrocaffeic acid-3-O-glucuronide<br>• 4-O-Caffeoylquinic acid-O-sulfate<br>• Caffeic acid-4-O-sulfate<br>• Dihydroferulic acid-4-O-sulfate<br>• Caffeic acid-3-O-sulfate<br>• Dihydrocaffeic acid<br>• Dihydroferulic acid-4-O-glucuronide<br>• Ferulic acid-4-O-sulfate<br>• 5-O-Caffeoylquinic acid<br>• 3-O-Feruloylquinic acid<br>• Isoferulic acid-3-O-sulfate<br>• Dihydro(iso)ferulic acid-3-O-glucuronide<br>• Isoferulic acid-3-O-glucuronide<br>• Feruloylglycine<br>• 3-O-Caffeoylquinic acid lactone-O-sulfate<br>• 4-O-Caffeoylquinic acid lactone-O-sulfate<br>• 4-O-Feruloylquinic acid<br>• Dihydroferulic acid<br>• 5-O-Feruloylquinic acid	(202)

Summary of Targeted Studies Presenting Candidate FIBs for Fermented Foods								
**Fermented Food**	**All interventions**	**Dose**	**Study Design**	***n***	**Analytical Method**	**Biosample**	**Candidate FIBs[a]**	**Reference**
Coffee	Instant coffee in water vs. instant coffee in milk vs. water	4 g in 200 mL water or milk	Acute, randomized, crossover	5 healthy volunteers	HPLC-DAD, LC-MS (Targeted)	Urine	• Hippuric acid<br>• 3,4-Dihydroxyphenylacetic acid<br>• Dihydrocaffeic acid<br>• Vanillic acid<br>• Gallic acid<br>• Isoferulic acid<br>• 4-Hydroxybenzoic acid<br>• 2,4-Hydroxybenzoic acid<br>• Trans-3-hydroxycinnamic acid<br>• P-Coumaric acid<br>• Syringic acid<br>• Sinapinic acid	(203)
Coffee	Freshly brewed coffee	30 g in 500 mL water	Acute intervention	10 healthy volunteers	HPLC-ESI-MS/MS (Targeted)	Urine	• Nicotinic acid<br>• Nicotinamide<br>• N-methylnicotinamide<br>• N-methyl-2-pyridone-5-carboxamide<br>• Nicotinuric acid	(204)
Coffee	Green-roasted coffee	3.5 gin 250 mL water	Acute intervention	12 healthy volunteers	HPLC-DAD, LC-MS-QTOF (Targeted)	Plasma, urine	• Caffeine<br>• Paraxanthine<br>• Theobromine<br>• Theophylline<br>• 1-Methylxanthine<br>• 3-Methylxanthine<br>• 7-Methylxanthine<br>• 1-Methyluric acid<br>• 1,3-Methyluric acid<br>• 1,7-Methyluric acid<br>• 1,3,7-Methyluric acid	(205)
Coffee	Instant coffee	8 g in 400 mL water	Acute intervention	10 healthy volunteers	LC-ESI-MS/MS (Targeted 56 phenolic compounds)	Plasma	• Total phenolic acids, methyls, glucuronides, sulfates, and lactones (ferulic acid, caffeic acid, and all metabolites thereof)	(206)
Coffee	Instant coffee	4 g in 400 mL water	Acute intervention	9 healthy volunteers	ESI-LC-ESI-MS/MS (Targeted)	Plasma	• Ferulic acid<br>• Caffeic acid<br>• Isoferulic acid<br>• Dihydrocaffeic acid<br>• Dihydroferulic acid	(207)
Coffee	Instant coffee vs. green tea	4 g in 400 mL water; 1.25% tea infusion	Acute crossover	9 healthy volunteers	ESI-LC-ESI-MS/MS (Targeted)	Plasma	• Ferulic acid<br>• Caffeic acid<br>• Isoferulic acid<br>• Dihydrocaffeic acid<br>• Dihydroferulic acid	(208)

Summary of Targeted Studies Presenting Candidate FIBs for Fermented Foods								
**Fermented Food**	**All interventions**	**Dose**	**Study Design**	***n***	**Analytical Method**	**Biosample**	**Candidate FIBs[a]**	**Reference**
Coffee	Instant coffee vs. instant coffee in whole milk vs. instant coffee with sugar and non-dairy creamer vs. no coffee	4 g in 400 mL water or 360 mL water + 40 mL whole milk or 30.5 g premixed instant coffee with sugar	Acute, randomized, crossover	9 healthy volunteers	ESI-LC-ESI-MS/MS (Targeted)	Plasma	• Ferulic acid<br>• Caffeic acid<br>• Isoferulic acid	(209)
Coffee	Coffee	0, 1, 2, or 3 cups/day	Cross-sectional	15 healthy men	LC-MS/MS (Targeted)	Urine	• Hippuric acid	(210)
Coffee	Nescafe Green Blend coffee	3.5 g in 240 mL water	Acute intervention	12 healthy volunteers	LC-MS-QTOF (Targeted)	Plasma, urine	• Caffeic acid and metabolites<br>• Ferulic acid and metabolites<br>• Coumaric acid and metabolites<br>• Dimethoxycinnamic acid and metabolites<br>• Lactone derivatives<br>• Phenolic acids	(211)
Coffee	Coffee	Caffeinated coffee, other caffeinated drinks, decaffeinated coffee consumption (never, 1-4X/month, 1-4X/week, 5X/week, ≥1X/day) (from FFQ)	Association study	598 volunteers rom the SKIPOGH cohort	HPLC-MS/MS (Targeted)	Urine	• Caffeine<br>• Paraxanthine<br>• Theophylline	(212)
Coffee	Instant coffee	3.4 g in 200 mL water	Acute intervention	11 healthy volunteers	HPLC-PDA-MS (Targeted)	Urine	• Caffeic acid-3-O-sulfate<br>• Caffeic acid-3-O-sulfate<br>• Ferulic acid-4-O-sulfate<br>• Isoferulic acid-3-O-glucuronide<br>• Isoferulic acid-3-O-sulfate<br>• Dihydrocaffeic acid-3-O-glucuronide<br>• Dihydrocaffeic acid-3-O-sulfate<br>• Dihydroferulic acid<br>• Dihydroferulic acid-4-O-glucuronide<br>• Dihydroferulic acid-4-O-sulfate	(213)
Coffee	Coffee enema vs. ready-to-drink coffee beverage	500 mL (coffee enema); 180 mL (RTD coffee)	Acute crossover	11 healthy men	HPLC-UV (Targeted)	Plasma	• Caffeine	(214)

Summary of Targeted Studies Presenting Candidate FIBs for Fermented Foods								
**Fermented Food**	**All interventions**	**Dose**	**Study Design**	***n***	**Analytical Method**	**Biosample**	**Candidate FIBs[a]**	**Reference**
Coffee	Decaffeinated coffee with high chlorogenic acids vs. medium vs. low	Coffee dose not reported; 4525, 2219, and 1052 umol chlorogenic acids in high, medium, and low doses, respectively	Acute, randomized, double-blind, crossover	5 healthy womn	HPLC-DAD-ESI-MS, ESI-MS/MS (Targeted)	Plasma, ileal effluent, urine	• Chlorogenic acid metabolites (caffeoylquinic acids, feruloylquinic acids, caffeic acids, dihydrocaffeic acids, ferulic acids, isoferulic acids, dihydroferulic acids)	(215)
Coffee	Instant coffee	400 mL (1% w/v)	Acute intervention	10 healthy volunteers	LC-MS (Targeted)	Plasma	• Caffeic acid<br>• Dihydrocaffeic acid<br>• Ferulic acid<br>• Isoferulic acid<br>• Dihydroferulic acid<br>• Dimethoxycinnamic acid<br>• Dimethoxydihydrocinnamic acid	(216)
Coffee	Coffee	2 cups	Acute intervention	14 healthy volunteers	2D-HR-GC-MS (Targeted)	Urine	• 4-Ethylguaiacol<br>• 4-Vinylguaiacol<br>• (E)-Beta-damascenone<br>• Dimethyl trisulfide<br>• Furfuryl alcohol<br>• Guaiacol<br>• Indole<br>• Methional<br>• Oct-1-en-3-one<br>• Skatole<br>• Vanillin	(217)
Coffee	Mocha coffee vs. caffeinated soft drink vs. low-dose caffeine in aqeuous solution vs. high-dose caffeine in aqueous solution	190 mL (soft drink), 50 mL (mocha), 70 mL (aqueous solution	Acute, randomized, crossover	4 healthy men	HPLC (detector not specified) (Targeted)	Plasma	• Caffeine<br>• Theophylline<br>• Paraxanthine<br>• Theobromine<br>• 1,3-Dimethylxanthine<br>• 1,7-Dimethylxanthine<br>• 3,7-Dimethylxanthine	(218)
Coffee	Coffee (brewed or canned)	150 mL (brewed coffee) or 187 mL (canned coffee)	Acute intervention	10 healthy volunteers	FOX-I method (Targeted)	Urine	• Hydrogen peroxide	(219)

Summary of Targeted Studies Presenting Candidate FIBs for Fermented Foods								
**Fermented Food**	**All interventions**	**Dose**	**Study Design**	***n***	**Analytical Method**	**Biosample**	**Candidate FIBs[a]**	**Reference**
Coffee	Instant coffee	2.5 g instant coffee in 200 mL water	Acute intervention	10 healthy volunteers	FOX assay (Targeted)	Urine	• Hydrogen peroxide	(220)
Coffee	Instant coffee	2 cups, each with 4 g instant coffee powder in 250 mL water	Acute intervention	5 healthy men	HPLC-UV (Targeted)	Urine	• Caffeic acid<br>• Ferulic acid<br>• Isoferulic acid<br>• Dihydroferulic acid<br>• 3-4-Hydroxy-3-methoxyphenyl-propanoic acid<br>• Vanillic acid<br>• Hippuric acid<br>• 3-Hydroxyhippuric acid	(221)
Coffee	High roasted coffee vs. low roasted coffee vs. unroasted coffee vs. in vitro hydrolyzed unroasted coffee	3.4 to 4.5 g of instant coffee in 200 mL water	Acute, randomized, double-blind, crossover	12 healthy volunteers	LC-ESI-MS/MS (Targeted)	Plasma	• Dihydroferulic acid<br>• Caffeic acid-3-O-sulfate<br>• Isoferulic-3-O-glucuronide<br>• 5-4-Dihydro-m-coumaric acid	(222)
Coffee	Decaffeinated coffee vs. regular coffee vs. stronger coffee vs. hot water vs. no intervention	17.5 to 25 g of coffee in 300 mL water	Acute, randomized, single-blind, crossover	8 healthy volunteers	HPLC (detector not specified) (Targeted)	Plasma	• Caffeine	(223)
Coffee	Coffee	4X7.5 g coffee pads and 500 mL water	Acute intervention	10 healthy volunteers	HPLC-ESI-MS/MS (Targeted)	Urine	• Pyrazine-2-carboxylic acid<br>• 5-Hydroxypyrazine-2-carboxylic acid<br>• 5-Methylpyrazine-2-carboxylic acid<br>• 6-Methylpyrazine-2-carboxylic acid	(224)
Coffee	Coffee drinkers vs. non-coffee drinkers	350 mL coffee or water	Acute, parallel	6 healthy volunteers	UPLC-HDMS (Untargeted)	Urine	• Trigonelline<br>• N-methylpyridinium<br>• Dimethylxanthines<br>• Monomethylxanthines<br>• 1,3-Dimethyluric acid<br>• 1,7-Dimethyluric acid<br>• Ferulic acid conjugates	(225)
	Caffeinated coffee	48 g coffee powder in 900 mL water	Acute intervention	13 healthy volunteers	UPLC-MS/MS (Targeted)	Plasma	• 5-O-Caffeoyl quinic acid<br>• Ferulic acid<br>• Iso-ferulic acid<br>• Feruloylsulfate<br>• Iso-feruloylsulfate	

Summary of Targeted Studies Presenting Candidate FIBs for Fermented Foods								
**Fermented Food**	**All interventions**	**Dose**	**Study Design**	***n***	**Analytical Method**	**Biosample**	**Candidate FIBs[a]**	**Reference**
							• Feruloylglucuronide<br>• Feruloylglycine<br>• Dihydroferulic acid<br>• Dihydroferuloylsulfate<br>• Dihydroferuloylglucuronide<br>• Dihydrocaffeoylsulfate (sum of isomers)<br>• Catecholsulfate<br>• Catecholglucuronide<br>• Guaiacolsulfate<br>• Guaiacolglucuronide<br>• Trigonelline<br>• N-methylpyridinium<br>• N-methylnicotinamide<br>• N-methyl-4-pyridone-5-carboxamide<br>• N-methyl-2-pyridone-5-carboxamide<br>• Caffeine<br>• Theophylline<br>• Paraxanthine<br>• Theobromine<br>• 3-Methylxanthine<br>• 7-Methylxanthine<br>• 1,7-Dimethyluric acid	
Coffee, cocoa	Green tea vs. grape-skin extract vs. cocoa beverage vs. instant coffee coffee vs. grape fruit juice vs. orange juice vs. hot water	4 g (instant coffee), 10 g (cocoa powder) in 200 mL water	Acute, randomized, crossover	9 healthy volunteers	HPLC-ESI-MS/MS (Targeted)	Urine	• Chlorogenic acid<br>• Caffeic acid<br>• M-coumaric acid<br>• 4-O-methylgallic acid<br>• Epicatechin<br>• Naringenin<br>• Enterodiol<br>• Enterolactone	(226)
Coffee, wine	Coffee, chocolate, wine, dark bread, and other conventional foods	Continuous intake of various foods (from 2-day dietary record)	Association study	53 volunteers from the SU.VI.MAX cohort	HPLC-ESI-MS/MS (Targeted)	Urine	• Chlorogenic acid<br>• Caffeic acid<br>• Gallic acid<br>• 4-O-methylgallic acid<br>• Enterolactone<br>• Enterodiol	(227)
Coffee, wine (polyphenol-rich foods)	Citrus fruits, apple and pear, olives, coffee tea, all wine, red wine	Continuous intake of citrus fruits, apple and pear, olives, coffee tea, all	Association study	475 volunteers from the EPIC cohort	U(H)PLC-ESI-MS/MS (Targeted 34 polyphenols)	Urine	• Protocatechuic acid, 3,4-dihydroxyphenylpropionic acid, ferulic acid, and caffeic acid highly associated with coffee intake (also: gallic acid, apigenin, quercetin, homovanillic acid, protocatechuic acid, m-coumaric acid, hydroxytyrosol, and daidzein, based on ranked method)	(228)

Summary of Targeted Studies Presenting Candidate FIBs for Fermented Foods								
**Fermented Food**	**All interventions**	**Dose**	**Study Design**	***n***	**Analytical Method**	**Biosample**	**Candidate FIBs[a]**	**Reference**
		wine, red wine (from 24-h recalls and dietary questionnaire)					• Hydroxytyrosol, tyrosol, resveratrol, gallic acid, and gallic acid ethyl ester highly associated with wine/red wine intake (also: homovanillic acid, 3-hydroxybenzoic acid, naringenin, 3,4-dihydroxyphenylpropanoic acid, 3,4-dihydroxyphenylacetic acid, p-coumaric acid, enterolactone, and catechin, based on ranked method)	
Coffee, wine, cocoa (polyphenol-rich foods)	Coffee, tea, red wine, citrus fruit, apples and pears, and chocolate products	Continuous intake of coffee, tea, red wine, citrus fruit, apples and pears, and chocolate products (from 24-h recall and FFQ)	Association study	481 volunteers from the EPIC cohort	U(H)PLC-QTOF-MS (Targeted)	Urine	• Dihydroferulic acid sulfate, guaiacol glucuronide, feruloylquinic acid, ferulic acid sulfate, feruloylquinic acid glucuronide, 3-O-caffeoylquinic acid, p-coumaric acid sulfate, caffeic acid sulfate, ferulic acid glucuronide, hydroxyhippuric acid, dihydrocaffeic acid sulfate, m-coumaric acid sulfate, dihydroferulic acid glucuronide, p-hydroxyphenyllactic acid, guaiacol sulfate, ethylcatechol glucuronide associated with coffee intake<br>• M-coumaric acid, gallic acid ethyl ester sulfate, hydroxytyrosol sulfate, dihydroresveratrol glucuronide, syringic acid sulfate, methylgallic acid sulfate, 4-O-methylgallic acid associated with red wine intake<br>• Methyl(epi)catechin sulfate, 4-hydroxy-(3'4'-dihydroxyphenyl)valeric acid sulfate, dihydroxyphenyl-gamma-valerolactone glucuronide, vanillic acid sulfate associated with chocolate intake	(229)
Coffee, bread, cheese (general diet)	Wholegrain bread, non-wholegrain bread, lowfat cheese, highfat cheese, regular coffee, decaffeinated coffee, and other (non-fermented) food groups	Continuous intake of 45 different food groups (from FFQ)	Association study	2380 volunteers from the EPIC-Potsdam cohort	FIA-MS/MS (Targeted 127 metabolites, including acylcarnitines, amino acids, diacyl-phosphatidylcholines, acyl-alkyl-phosphatidylcholines, lyso-phosphatidylcholines, sphingomyelins, and hexoses)	Serum	• Amino acids associated with all dairy<br>• Hexoses with non-wholegrain bread<br>• Acyl-alkyl-phosphatidylcholines and lyso-phosphatidylcholines with high-fat dairy<br>• Sphingomyelins with coffee	(230)
Coffee, wine	Coffee, tea, wine, cereal, fruit, vegetable,	Continuous intake of coffee, tea, wine, cereal, fruit, vegetable, other	Association study	61 volunteers, most with CV risk; 2672	HPLC-UV (Targeted)	Plasma	• Zeaxanthin<br>• Beta-carotene<br>• Alpha-carotene	(231)

Summary of Targeted Studies Presenting Candidate FIBs for Fermented Foods								
**Fermented Food**	**All interventions**	**Dose**	**Study Design**	***n***	**Analytical Method**	**Biosample**	**Candidate FIBs[a]**	**Reference**
	other food intake	foods (from 7-day weighted diet records); from FFQ		volunteers from the NORKOST 2 cohort				
Coffee, cocoa	Espresso coffee vs. espresso coffee + cocoa-based products containing coffee	3 cups espresso vs. 1 cup espresso + 2 cups cocoa-based products containing coffee	1-month, randomized, crossover	21 healthy volunteers	UHPLC-ESI-MS (Targeted)	Plasma, urine	• Trigonelline (coffee)<br>• N-methylpyridinium (coffee)<br>• N-methylnicotinamide (coffee)<br>• N-methyl-4-pyridone-5-carboxamide (coffee)	(232)
Vinegar	Vinegar capsules vs. vinegar drink vs. non-carbonated mineral water	9 capsules (750 mg acetic acid); 100 mL vinegar (750 mg acetic acid); 150 mL water	Acute, randomized, controlled, crossover	30 healthy volunteers	GC-2010 (Targeted)	Serum	• Acetate	(233)
Vinegar	Red wine vinegar vs. apple cider vinegar tablet	4 tablespoons/day (3.6 g acetic acid/day); 2 tablets/day (0.045 g acetic acid/day)	8-week, randomized, controlled, parallel	45 healthy volunteers with high waist circumference and at increased risk for metabolic complications	GC-MS, LC-MS/MS (Targeted)	Plasma	• Amino valerate<br>• Indole-3-acetic acid<br>• dTMP	(234)
Sauerkraut	Sauerkraut	5-6 g/kg body weight	Acute intervention	4 volunteers	LC-MS (Targeted)	Plasma, urine	• D-phenyllactic acid	(235)
Cider	Thatchers Redstreak apple cider	500 mL	Acute intervention	9 healthy and 5 ileostomy volunteers	HPLC-PDA-MS (Targeted)	Plasma, urine, ileal fluid	• Phloretin-2'-glucuronide<br>• Phloretin-O-glucuronide-O-sulfate<br>• Phoretin-O-sulfate	(236)
Fermented orange juice	Fermented orange juice (fermented using yeast: *Pichia kluyveri* var. *kluyveri*) vs. unfermented orange juice	500 mL	Acute, randomized, controlled, crossover	7 healthy volunteers	HPLC-DAD (Targeted)	Plasma	• Beta-cryptoxanthin<br>• Lutein	(237)

Summary of Targeted Studies Presenting Candidate FIBs for Fermented Foods								
**Fermented Food**	**All interventions**	**Dose**	**Study Design**	***n***	**Analytical Method**	**Biosample**	**Candidate FIBs[a]**	**Reference**
Fermented ginseng	Fermented vs. non-fermented ginseng	3 g	Open-label, randomized, single-dose, crossover	24 healthy volunteers	LC-MS/MS (Targeted)	Plasma	• Ginsenoside metabolite IH-901 (20-O-beta-D-glucopyranosyl-20(S)-protopanaxodiol)	(238)
Fermented Red Beet Juice	Fermented red beet juice	200 mL/60 kg body weight	6-week, uncontrolled, intervention	24 healthy volunteers	Micro-HPLC-MS/MS (Targeted)	Plasma, urine	• Betalain and derivatives (isobetanin, isobetanidin, 17-decarboxy-betanin, 17-decarboxy-isobetanin, 17-decarboxy-neobetanin, neobetanin, 2,17-bidecarboxyneobetanin, 2,15,17-tiidecarboxyneobetanin, 2,17-bidecarboxyneobetanin, 2,15,17-tiidecarboxybetanin, 6'-O-feruloyl-betanin/isobetanin, , 2,15,17-tiidecarboxy-2,3-dehydro-neobetanin)	(239)
Salgam, boza, kimiz, kefir	Salgam, boza, kimiz, kefir	300 mL	Acute, crossover	12 healthy volunteers	HS-GC-FID (blood ethanol); LC-MS/MS (urine ethyl glucuronide and sulfate) (Targeted)	Whole blood, urine	• No change in blood alcohol levels or ethanol metabolites in urine	(240)
Fermented red cabbage	Fermented red cabbage vs. fresh red cabbage	240 g	Acute, randomized, controlled, crossover	13 healthy volunteers	HPLC-MS/MS (Targeted)	Plasma, urine	• Cyanidin derivatives	(241)
Fermented rooibos tea	Fermented vs. unfermented rooibos tea	500 mL	Acute, randomized, controlled, crossover	10 healthy volunteers	HPLC-MS (Targeted)	Plasma, urine	• C-linked dihydrochalcone and flavanone glucosides (O-methyl, sulfate, glucuronide metabolites of aspalathin and eriodictyol-O-sulfate	(242)
Pu-erh tea	Pu-erh tea	200 mL (containing 10 g of tea powder)	Acute and 2-week, randomized, controlled	20 healthy volunteers	U(H)PLC-QTOF-MS (Targeted)	Urine	• Inositol<br>• Myristic acid<br>• 5-Hydroxytryptophan<br>• 4-Methyloxyphenylacetic acid<br>• Pyroglutamic acid	(243)

Abbreviations: AR, alkenylresorcinol; CE, capillary electrophoresis; CEAD, coulometric electrode array detector; CVD, cardiovascular disease; DHA, docosahexaenoic acid; DAD, diode array detector; DHBA, 3,5-dihydroxybenzoic acid; DHPPA, 3-(3,5-dihydroxyphenyl)-1-propanoic acid); DHPPTA, 5-(3,5-dihydroxyphenyl) pentanoic acid; DHPV, 5-(3,4-dihydroxyphenyl)-γ-valerolactone; EAD, enzyme activity/affinity detector; ECD, electrochemical detector; ELISA, enzyme-linked immunosorbent assay; EPA, eicosapentaenoic acid; ESI, electrospray ionization; FFQ, food frequency questionnaire; FIA, flow injection analysis; FIB, food intake biomarker; FID, flame ionization detector; FLD, fluorescence/fluorometric detector; FOX, ferrous ion oxidation xylenol orange; GC, gas chromatography; GLC, gas-liquid chromatography; HDMS, high definition mass spectrometry; HILIC, hydrophilic interaction liquid chromatography; HPLC, high-performance liquid chromatography; HR, high resolution; HS, headspace; LC, liquid chromatography; LDL, low-density lipoprotein; MHPV, 3′-methoxy-4′-hydroxyphenylvalerolactone; MS, mass spectrometry; MS/MS, tandem mass spectrometry; MUFA, monounsaturated fatty acid; NMR, nuclear magnetic resonance; PC, phosphatidylcholine; PDA, photometric diode array; PUFA, polyunsaturated fatty acid; QTOF, quadrupole time-of-flight; RP, reverse phase; SCFA, short chain fatty acid; SFA, saturated fatty acid; TOF, time-of-flight; U(H)PLC, ultra-high performance liquid chromatography; UV, ultraviolet; VIS, visible.

[a] Candidate FIBs that are significantly increased compared to control or baseline in each study are reported.

References

1. Monošík R, Dragsted LO. A versatile UHPLC-MSMS method for simultaneous quantification of various alcohol intake related compounds in human urine and blood. Anal Methods. 2016;8(38):6865-71.
2. Rodda LN, Gerostamoulos D, Drummer OH. Pharmacokinetics of reduced iso-alpha-acids in volunteers following clear bottled beer consumption. Forensic Sci Int. 2015;250:37-43.
3. Quifer-Rada P, Martinez-Huelamo M, Jauregui O, Chiva-Blanch G, Estruch R, Lamuela-Raventos RM. Analytical condition setting a crucial step in the quantification of unstable polyphenols in acidic conditions: analyzing prenylflavanoids in biological samples by liquid chromatography-electrospray ionization triple quadruple mass spectrometry. Anal Chem. 2013;85(11):5547-54.
4. Inokuchi T, Ka T, Yamamoto A, Takahashi S, Tsutsumi Z, Moriwaki Y, et al. Effects of allopurinol on beer-induced increases in plasma concentrations of purine bases and uridine. Nucleosides Nucleotides Nucleic Acids. 2008;27(6):601-3.
5. Maldonado MD, Moreno H, Calvo JR. Melatonin present in beer contributes to increase the levels of melatonin and antioxidant capacity of the human serum. Clin Nutr. 2009;28(2):188-91.
6. Lindenthal B, von Bergmann K. Urinary excretion and serum concentration of mevalonic acid during acute intake of alcohol. Metabolism. 2000;49(1):62-6.
7. Rodda LN, Gerostamoulos D, Drummer OH. Pharmacokinetics of Iso-alpha-Acids in Volunteers Following the Consumption of Beer. J Anal Toxicol. 2014;38(6):354-9.
8. Quifer-Rada P, Martinez-Huelamo M, Chiva-Blanch G, Jauregui O, Estruch R, Lamuela-Raventos RM. Urinary isoxanthohumol is a specific and accurate biomarker of beer consumption. J Nutr. 2014;144(4):484-8.
9. Thierauf A, Gnann H, Wohlfarth A, Auwarter V, Perdekamp MG, Buttler K-J, et al. Urine tested positive for ethyl glucuronide and ethyl sulphate after the consumption of "non-alcoholic" beer. Forensic Sci Int. 2010;202(1-3):82-5.
10. Bourne L, Paganga G, Baxter D, Hughes P, Rice-Evans C. Absorption of ferulic acid from low-alcohol beer. Free Radic Res. 2000;32(3):273-80.
11. Dahl H, Stephanson N, Beck O, Helander A. Comparison of urinary excretion characteristics of ethanol and ethyl glucuronide. J Anal Toxicol. 2002;26(4):201-4.
12. Jones AW. Concentration-time profiles of ethanol in capillary blood after ingestion of beer. J Forensic Sci Soc. 1991;31(4):429-39.
13. Neuteboom W, Vis AA. The effects of low alcohol beers on the blood alcohol concentration. Blutalkohol. 1991;28(6):393-6.
14. Roine RP, Gentry RT, Lim RT, Jr., Helkkonen E, Salaspuro M, Lieber CS. Comparison of blood alcohol concentrations after beer and whiskey. Alcohol Clin Exp Res. 1993;17(3):709-11.
15. Tsuchiya H, Yamada K, Todoriki H, Hayashi T. Urinary excretion of tetrahydro-β-carbolines influenced by food and beverage ingestion implies their exogenous supply via dietary sources. J Nutr Biochem. 1996;7(4):237-42.
16. Tsukamoto S, Muto T, Nagoya T, Shimamura M, Saito M, Tainaka H. Determinations of ethanol, acetaldehyde and acetate in blood and urine during alcohol oxidation in man. Alcohol Alcohol. 1989;24(2):101-8.
17. Tsukamoto S, Kanegae T, Uchigasaki S, Kitazawa M, Fujioka T, Fujioka S, et al. Changes in free and bound alcohol metabolites in the urine during ethanol oxidation. Arukoru Kenkyuto Yakubutsu Ison. 1993;28(6):441-52.
18. Lekskulchai V, Rattanawibool S. Blood alcohol concentrations after "one standard drink" in Thai healthy volunteers. J Med Assoc Thai. 2007;90(6):1137-42.
19. Muraguri N, Kaviti JN, Patel HA, Shaja NK. Alcohol changes in blood and urine after the consumption of local beers. East Afr Med J. 1975;52(11):625-30.
20. Sommer T, Göen T, Budnik N, Pischetsrieder M. Absorption, Biokinetics, and Metabolism of the Dopamine D2 Receptor Agonist Hordenine (N, N-Dimethyltyramine) after Beer Consumption in Humans. J Agric Food Chem. 2020;68(7):1998-2006.
21. Du D, Bruno R, Blizzard L, Venn A, Dwyer T, Smith KJ, et al. The metabolomic signatures of alcohol consumption in young adults. Eur J Prev Cardiol. 2020;27(8):840-9.
22. van der Gaag MS, van den Berg R, van den Berg H, Schaafsma G, Hendriks HF. Moderate consumption of beer, red wine and spirits has counteracting effects on plasma antioxidants in middle-aged men. Eur J Clin Nutr. 2000;54(7):586-91.
23. Prickett CD, Lister E, Collins M, Trevithick-Sutton CC, Hirst M, Vinson JA, et al. Alcohol: Friend or Foe? Alcoholic Beverage Hormesis for Cataract and Atherosclerosis is Related to Plasma Antioxidant Activity. Nonlinearity Biol Toxicol Med. 2004;2(4):353-70.
24. di Giuseppe R, de Lorgeril M, Salen P, Laporte F, Di Castelnuovo A, Krogh V, et al. Alcohol consumption and n-3 polyunsaturated fatty acids in healthy men and women from 3 European populations. Am J Clin Nutr. 2009;89(1):354-62.
25. Mitchell MC, Jr., Teigen EL, Ramchandani VA. Absorption and peak blood alcohol concentration after drinking beer, wine, or spirits. Alcohol Clin Exp Res. 2014;38(5):1200-4.

26. Nogueira LC, Couri S, Trugo NF, Lollo PCB. The effect of different alcoholic beverages on blood alcohol levels, plasma insulin and plasma glucose in humans. Food Chem. 2014;158:527-33.
27. Gustafson R, Kallmen H. The blood alcohol curve as a function of time and type of beverage: methodological considerations. Drug Alcohol Depend. 1988;21(3):243-6.
28. Albermann ME, Musshoff F, Doberentz E, Heese P, Banger M, Madea B. Preliminary investigations on ethyl glucuronide and ethyl sulfate cutoffs for detecting alcohol consumption on the basis of an ingestion experiment and on data from withdrawal treatment. Int J Legal Med. 2012;126(5):757-64.
29. Bendtsen P, Jones AW, Helander A. Urinary excretion of methanol and 5-hydroxytryptophol as biochemical markers of recent drinking in the hangover state. Alcohol Alcohol. 1998;33(4):431-8.
30. Boto-Ordonez M, Urpi-Sarda M, Queipo-Ortuno MI, Corella D, Tinahones FJ, Estruch R, et al. Microbial metabolomic fingerprinting in urine after regular dealcoholized red wine consumption in humans. J Agric Food Chem. 2013;61(38):9166-75.
31. Dresen S, Weinmann W, Wurst FM. Forensic confirmatory analysis of ethyl sulfate - A new marker for alcohol consumption - By liquid-chromatography/electrospray ionization/tandem mass spectrometry. J Am Soc Mass Spectrom. 2004;15(11):1644-8.
32. Urpi-Sarda M, Zamora-Ros R, Lamuela-Raventos R, Cherubini A, Jauregui O, de la Torre R, et al. HPLC-tandem mass spectrometric method to characterize resveratrol metabolism in humans. Clin Chem. 2007;53(2):292-9.
33. Rotches-Ribalta M, Urpi-Sarda M, Llorach R, Boto-Ordonez M, Jauregui O, Chiva-Blanch G, et al. Gut and microbial resveratrol metabolite profiling after moderate long-term consumption of red wine versus dealcoholized red wine in humans by an optimized ultra-high-pressure liquid chromatography tandem mass spectrometry method. J Chromatogr A. 2012;1265:105-13.
34. Urpi-Sarda M, Boto-Ordonez M, Queipo-Ortuno MI, Tulipani S, Corella D, Estruch R, et al. Phenolic and microbial-targeted metabolomics to discovering and evaluating wine intake biomarkers in human urine and plasma. Electrophoresis. 2015;36(18):2259-68.
35. Gresele P, Pignatelli P, Guglielmini G, Carnevale R, Mezzasoma AM, Ghiselli A, et al. Resveratrol, at concentrations attainable with moderate wine consumption, stimulates human platelet nitric oxide production. J Nutr. 2008;138(9):1602-8.
36. de la Torre R, Covas MI, Pujadas MA, Fito M, Farre M. Is dopamine behind the health benefits of red wine? Eur J Nutr. 2006;45(5):307-10.
37. Munoz-Gonzalez I, Jimenez-Giron A, Martin-Alvarez PJ, Bartolome B, Moreno-Arribas MV. Profiling of microbial-derived phenolic metabolites in human feces after moderate red wine intake. J Agric Food Chem. 2013;61(39):9470-9.
38. Zamora-Ros R, Rothwell JA, Achaintre D, Ferrari P, Boutron-Ruault M-C, Mancini FR, et al. Evaluation of urinary resveratrol as a biomarker of dietary resveratrol intake in the European Prospective Investigation into Cancer and Nutrition (EPIC) study. Br J Nutr. 2017;117(11):1596-602.
39. Nardini M, Forte M, Vrhovsek U, Mattivi F, Viola R, Scaccini C. White wine phenolics are absorbed and extensively metabolized in humans. J Agric Food Chem. 2009;57(7):2711-8.
40. Halter CC, Dresen S, Auwaerter V, Wurst FM, Weinmann W. Kinetics in serum and urinary excretion of ethyl sulfate and ethyl glucuronide after medium dose ethanol intake. Int J Legal Med. 2008;122(2):123-8.
41. Boronat A, Martinez-Huelamo M, Cobos A, de la Torre R. Wine and Olive Oil Phenolic Compounds Interaction in Humans. Diseases. 2018;6(3).
42. Simonetti P, Gardana C, Pietta P. Caffeic acid as biomarker of red wine intake. Methods Enzymol. 2001;335:122-30.
43. Vitaglione P, Sforza S, Galaverna G, Ghidini C, Caporaso N, Vescovi PP, et al. Bioavailability of trans-resveratrol from red wine in humans. Mol Nutr Food Res. 2005;49(5):495-504.
44. Jimenez-Giron A, Queipo-Ortuno MI, Boto-Ordonez M, Munoz-Gonzalez I, Sanchez-Patan F, Monagas M, et al. Comparative study of microbial-derived phenolic metabolites in human feces after intake of gin, red wine, and dealcoholized red wine. J Agric Food Chem. 2013;61(16):3909-15.
45. Donovan JL, Kasim-Karakas S, German JB, Waterhouse AL. Urinary excretion of catechin metabolites by human subjects after red wine consumption. Br J Nutr. 2002;87(1):31-7.
46. Kechagias S, Dernroth DN, Blomgren A, Hansson T, Isaksson A, Walther L, et al. Phosphatidylethanol Compared with Other Blood Tests as a Biomarker of Moderate Alcohol Consumption in Healthy Volunteers: A Prospective Randomized Study. Alcohol Alcohol. 2015;50(4):399-406.
47. Spaak J, Merlocco AC, Soleas GJ, Tomlinson G, Morris BL, Picton P, et al. Dose-related effects of red wine and alcohol on hemodynamics, sympathetic nerve activity, and arterial diameter. Am J Physiol Heart Circ Physiol. 2008;294(2):H605-12.
48. Regueiro J, Vallverdu-Queralt A, Simal-Gandara J, Estruch R, Lamuela-Raventos RM. Urinary tartaric acid as a potential biomarker for the dietary assessment of moderate wine consumption: a randomised controlled trial. Br J Nutr. 2014;111(9):1680-5.

49. Urquiaga I, Strobel P, Perez D, Martinez C, Cuevas A, Castillo O, et al. Mediterranean diet and red wine protect against oxidative damage in young volunteers. Atherosclerosis. 2010;211(2):694-9.
50. Zamora-Ros R, Urpi-Sarda M, Lamuela-Raventos RM, Estruch R, Martinez-Gonzalez MA, Bullo M, et al. Resveratrol metabolites in urine as a biomarker of wine intake in free-living subjects: The PREDIMED Study. Free Radic Biol Med. 2009;46(12):1562-6.
51. Regal P, Porto-Arias JJ, Lamas A, Paz L, Barreiro F, Cepeda A. LC-MS as a Tool to Overcome the Limitations of Self-Reported Dietary Assessments in the Determination of Wine Intake. Separations. 2017;4(2):7.
52. Zamora-Ros R, Urpi-Sarda M, Lamuela-Raventos RM, Estruch R, Vazquez-Agell M, Serrano-Martinez M, et al. Diagnostic performance of urinary resveratrol metabolites as a biomarker of moderate wine consumption. Clin Chem. 2006;52(7):1373-80.
53. Donovan JL, Bell JR, Kasim-Karakas S, German JB, Walzem RL, Hansen RJ, et al. Catechin is present as metabolites in human plasma after consumption of red wine. J Nutr. 1999;129(9):1662-8.
54. Bell JR, Donovan JL, Wong R, Waterhouse AL, German JB, Walzem RL, et al. (+)-Catechin in human plasma after ingestion of a single serving of reconstituted red wine. Am J Clin Nutr. 2000;71(1):103-8.
55. Schroder H, de la Torre R, Estruch R, Corella D, Martinez-Gonzalez MA, Salas-Salvado J, et al. Alcohol consumption is associated with high concentrations of urinary hydroxytyrosol. Am J Clin Nutr. 2009;90(5):1329-35.
56. Ortuño J, Covas MI, Farre M, Pujadas M, Fito M, Khymenets O, et al. Matrix effects on the bioavailability of resveratrol in humans. Food Chem. 2010;120(4):1123-30.
57. Regueiro J, Vallverdu-Queralt A, Simal-Gandara J, Estruch R, Lamuela-Raventos R. Development of a LC-ESI-MS/MS approach for the rapid quantification of main wine organic acids in human urine. J Agric Food Chem. 2013;61(27):6763-8.
58. Zamora-Ros R, Urpi-Sarda M, Lamuela-Raventos RM, Martinez-Gonzalez MA, Salas-Salvado J, Aros F, et al. High urinary levels of resveratrol metabolites are associated with a reduction in the prevalence of cardiovascular risk factors in high-risk patients. Pharmacol Res. 2012;65(6):615-20.
59. Urpí-Sardà M, Jáuregui O, Lamuela-Raventós RM, Jaeger W, Miksits M, Covas MI, et al. Uptake of diet resveratrol into the human low-density lipoprotein. Identification and quantification of resveratrol metabolites by liquid chromatography coupled with tandem mass spectrometry. Anal Chem. 2005;77(10):3149-55.
60. Ruidavets J, Teissedre P, Ferrieres J, Carando S, Bougard G, Cabanis J. Catechin in the Mediterranean diet: vegetable, fruit or wine? Atherosclerosis. 2000;153(1):107-17.
61. Tsang C, Higgins S, Duthie GG, Duthie SJ, Howie M, Mullen W, et al. The influence of moderate red wine consumption on antioxidant status and indices of oxidative stress associated with CHD in healthy volunteers. Br J Nutr. 2005;93(2):233-40.
62. Simonetti P, Gardana C, Pietta P. Plasma levels of caffeic acid and antioxidant status after red wine intake. J Agric Food Chem. 2001;49(12):5964-8.
63. Fernandes I, Marques C, Evora A, Cruz L, de Freitas V, Calhau C, et al. Pharmacokinetics of table and Port red wine anthocyanins: a crossover trial in healthy men. Food Funct. 2017;8(5):2030-7.
64. Cerda B, Tomas-Barberan FA, Espin JC. Metabolism of antioxidant and chemopreventive ellagitannins from strawberries, raspberries, walnuts, and oak-aged wine in humans: identification of biomarkers and individual variability. J Agric Food Chem. 2005;53(2):227-35.
65. Perez-Mana C, Farre M, Rodriguez-Morato J, Papaseit E, Pujadas M, Fito M, et al. Moderate consumption of wine, through both its phenolic compounds and alcohol content, promotes hydroxytyrosol endogenous generation in humans. A randomized controlled trial. Mol Nutr Food Res. 2015;59(6):1213-6.
66. Kronstrand R, Brinkhagen L, Nystrom FH. Ethyl glucuronide in human hair after daily consumption of 16 or 32 g of ethanol for 3 months. Forensic Sci Int. 2012;215(1-3):51-5.
67. Bitsch R, Netzel M, Frank T, Strass G, Bitsch I. Bioavailability and Biokinetics of Anthocyanins From Red Grape Juice and Red Wine. J Biomed Biotechnol. 2004;2004(5):293-8.
68. Bub A, Watzl B, Heeb D, Rechkemmer G, Briviba K. Malvidin-3-glucoside bioavailability in humans after ingestion of red wine, dealcoholized red wine and red grape juice. Eur J Nutr. 2001;40(3):113-20.
69. Duthie GG, Pedersen MW, Gardner PT, Morrice PC, Jenkinson AM, McPhail DB, et al. The effect of whisky and wine consumption on total phenol content and antioxidant capacity of plasma from healthy volunteers. Eur J Clin Nutr. 1998;52(10):733-6.
70. Frank T, Netzel M, Strass G, Bitsch R, Bitsch I. Bioavailability of anthocyanidin-3-glucosides following consumption of red wine and red grape juice. Can J Physiol Pharmacol. 2003;81(5):423-35.
71. Goll M, Schmitt G, Ganssmann B, Aderjan RE. Excretion profiles of ethyl glucuronide in human urine after internal dilution. J Anal Toxicol. 2002;26(5):262-6.

72. Hoiseth G, Yttredal B, Karinen R, Gjerde H, Christophersen A. Levels of ethyl glucuronide and ethyl sulfate in oral fluid, blood, and urine after use of mouthwash and ingestion of nonalcoholic wine. J Anal Toxicol. 2010;34(2):84-8.
73. Murdock HR, Jr. Blood glucose and alcohol levels after administration of wine to human subjects. Am J Clin Nutr. 1971;24(4):394-6.
74. Mizoi Y, Hishida S, Ijiri I, Maruyama J, Asakura S, Kijima T, et al. Individual differences in blood and breath acetaldehyde levels and urinary excretion of catecholamines after alcohol intake. Alcohol Clin Exp Res. 1980;4(4):354-60.
75. Teague C, Holmes E, Maibaum E, Nicholson J, Tang H, Chan Q, et al. Ethyl glucoside in human urine following dietary exposure: detection by 1H NMR spectroscopy as a result of metabonomic screening of humans. Analyst. 2004;129(3):259-64.
76. Pimentel G, Burton KJ, Rosikiewicz M, Freiburghaus C, von Ah U, Munger LH, et al. Blood lactose after dairy product intake in healthy men. Br J Nutr. 2017;118(12):1070-7.
77. Matsumoto M, Aranami A, Ishige A, Watanabe K, Benno Y. LKM512 yogurt consumption improves the intestinal environment and induces the T-helper type 1 cytokine in adult patients with intractable atopic dermatitis. Clin Exp Allergy. 2007;37(3):358-70.
78. Alvaro E, Andrieux C, Rochet V, Rigottier-Gois L, Lepercq P, Sutren M, et al. Composition and metabolism of the intestinal microbiota in consumers and non-consumers of yogurt. Br J Nutr. 2007;97(1):126-33.
79. Matsumoto M, Benno Y. Anti-inflammatory and antimutagenic activity of polyamines produced by Bifidobacterium lactis LKM512. Curr Top Nutraceutical Res. 2004;2(4):219-26.
80. Rizkalla SW, Luo J, Kabir M, Chevalier A, Pacher N, Slama G. Chronic consumption of fresh but not heated yogurt improves breath-hydrogen status and short-chain fatty acid profiles: a controlled study in healthy men with or without lactose maldigestion. Am J Clin Nutr. 2000;72(6):1474-9.
81. Marteau P, Flourie B, Pochart P, Chastang C, Desjeux JF, Rambaud JC. Effect of the microbial lactase (EC 3.2.1.23) activity in yoghurt on the intestinal absorption of lactose: an in vivo study in lactase-deficient humans. Br J Nutr. 1990;64(1):71-9.
82. Santaren ID, Watkins SM, Liese AD, Wagenknecht LE, Rewers MJ, Haffner SM, et al. Serum pentadecanoic acid (15:0), a short-term marker of dairy food intake, is inversely associated with incident type 2 diabetes and its underlying disorders. Am J Clin Nutr. 2014;100(6):1532-40.
83. Wolk A, Vessby B, Ljung H, Barrefors P. Evaluation of a biological marker of dairy fat intake. Am J Clin Nutr. 1998;68(2):291-5.
84. Dougkas A, Minihane AM, Givens DI, Reynolds CK, Yaqoob P. Differential effects of dairy snacks on appetite, but not overall energy intake. Br J Nutr. 2012;108(12):2274-85.
85. Abdullah MMH, Cyr A, Lepine M-C, Labonte M-E, Couture P, Jones PJH, et al. Recommended dairy product intake modulates circulating fatty acid profile in healthy adults: a multi-centre cross-over study. Br J Nutr. 2015;113(3):435-44.
86. Albani V, Celis-Morales C, O'Donovan CB, Walsh MC, Woolhead C, Forster H, et al. Within-person reproducibility and sensitivity to dietary change of C15:0 and C17:0 levels in dried blood spots: Data from the European Food4Me Study. Mol Nutr Food Res. 2017;61(10).
87. Rohrmann S, Linseisen J, Allenspach M, von Eckardstein A, Muller D. Plasma Concentrations of Trimethylamine-N-oxide Are Directly Associated with Dairy Food Consumption and Low-Grade Inflammation in a German Adult Population. J Nutr. 2016;146(2):283-9.
88. Hruby A, Dennis C, Jacques PF. Dairy Intake in 2 American Adult Cohorts Associates with Novel and Known Targeted and Nontargeted Circulating Metabolites. J Nutr. 2020;150(5):1272-83.
89. Warensjo E, Jansson J-H, Cederholm T, Boman K, Eliasson M, Hallmans G, et al. Biomarkers of milk fat and the risk of myocardial infarction in men and women: a prospective, matched case-control study. Am J Clin Nutr. 2010;92(1):194-202.
90. Ohya T. Identification of 4-methylspinaceamine, a pictet-spengler condensation reaction product of histamine with acetaldehyde, in fermented foods and its metabolite in human urine. J Agric Food Chem. 2006;54(18):6909-15.
91. Sharafedtinov KK, Plotnikova OA, Alexeeva RI, Sentsova TB, Songisepp E, Stsepetova J, et al. Hypocaloric diet supplemented with probiotic cheese improves body mass index and blood pressure indices of obese hypertensive patients - a randomized double-blind placebo-controlled pilot study. Nutr J. 2013;12:11.
92. Intorre F, Venneria E, Finotti E, Foddai MS, Toti E, Catasta G, et al. Fatty acid content of serum lipid fractions and blood lipids in normolipidaemic volunteers fed two types of cheese having different fat compositions: a pilot study. Int J Food Sci Nutr. 2013;64(2):185-93.
93. Kano M, Takayanagi T, Harada K, Sawada S, Ishikawa F. Bioavailability of isoflavones after ingestion of soy beverages in healthy adults. J Nutr. 2006;136(9):2291-6.
94. Xu X, Wang HJ, Murphy PA, Hendrich S. Neither background diet nor type of soy food affects short-term isoflavone bioavailability in women. J Nutr. 2000;130(4):798-801.
95. Schurgers LJ, Vermeer C. Determination of phylloquinone and menaquinones in food. Effect of food matrix on circulating vitamin K concentrations. Haemostasis. 2000;30(6):298-307.

96. Hutchins AM, Slavin JL, Lampe JW. Urinary isoflavonoid phytoestrogen and lignan excretion after consumption of fermented and unfermented soy products. J Am Diet Assoc. 1995;95(5):545-51.
97. Tsangalis D, Wilcox G, Shah NP, Stojanovska L. Bioavailability of isoflavone phytoestrogens in postmenopausal women consuming soya milk fermented with probiotic bifidobacteria. Br J Nutr. 2005;93(6):867-77.
98. Tsangalis D, Wilcox G, Shah NP, McGill AEJ, Stojanovska L. Urinary excretion of equol by postmenopausal women consuming soymilk fermented by probiotic bifidobacteria. Eur J Clin Nutr. 2007;61(3):438-41.
99. Chang Y, Choue R. Plasma pharmacokinetics and urinary excretion of isoflavones after ingestion of soy products with different aglycone/glucoside ratios in South Korean women. Nutr Res Pract. 2013;7(5):393-9.
100. Miura A, Sugiyama C, Sakakibara H, Simoi K, Goda T. Bioavailability of isoflavones from soy products in equol producers and non-producers in Japanese women. J Nutr Intermed Metab. 2016;6:41-7.
101. Okabe Y, Shimazu T, Tanimoto H. Higher bioavailability of isoflavones after a single ingestion of aglycone-rich fermented soybeans compared with glucoside-rich non-fermented soybeans in Japanese postmenopausal women. J Sci Food Agric. 2011;91(4):658-63.
102. Jou HJ, Tsai PJ, Tu JH, Wu WH. Stinky tofu as a rich source of bioavailable S-equol in Asian diets. J Funct Foods. 2013;5(2):651-9.
103. Nagino T, Kano M, Masuoka N, Kaga C, Anbe M, Miyazaki K, et al. Intake of a fermented soymilk beverage containing moderate levels of isoflavone aglycones enhances bioavailability of isoflavones in healthy premenopausal Japanese women: a double-blind, placebo-controlled, single-dose, crossover trial. Biosci Microbiota Food Health. 2016;35(1):9-17.
104. Kaneki M, Hodges SJ, Hosoi T, Fujiwara S, Lyons A, Crean SJ, et al. Japanese fermented soybean food as the major determinant of the large geographic difference in circulating levels of vitamin K2: possible implications for hip-fracture risk. Nutrition. 2001;17(4):315-21.
105. Maskarinec G, Watts K, Kagihara J, Hebshi SM, Franke AA. Urinary isoflavonoid excretion is similar after consuming soya milk and miso soup in Japanese-American women. Br J Nutr. 2008;100(2):424-9.
106. Slavin JL, Karr SC, Hutchins AM, Lampe JW. Influence of soybean processing, habitual diet, and soy dose on urinary isoflavonoid excretion. Am J Clin Nutr. 1998;68(6 Suppl):1492S-5S.
107. Koh E, Mitchell AE. Characterization of urinary isoflavone metabolites excreted after the consumption of soy flour or soybean paste using lc-(esi)ms/ms. J Food Biochem. 2011;35(5):1474-85.
108. Homma K, Wakana N, Suzuki Y, Nukui M, Daimatsu T, Tanaka E, et al. Treatment of natto, a fermented soybean preparation, to prevent excessive plasma vitamin K concentrations in patients taking warfarin. J Nutr Sci Vitaminol (Tokyo). 2006;52(5):297-301.
109. Mori M, Okabe Y, Tanimoto H, Shimazu T, Mori H, Yamori Y. Isoflavones as putative anti-aging food factors in Asia and effects of isoflavone aglycone-rich fermented soybeans on bone and glucose metabolisms in post-menopausal women. Geriatr Gerontol Int. 2008;8(SUPPL. 1):S8-S15.
110. Frankenfeld CL, Lampe JW, Shannon J, Gao DL, Ray RM, Prunty J, et al. Frequency of soy food consumption and serum isoflavone concentrations among Chinese women in Shanghai. Public Health Nutr. 2004;7(6):765-72.
111. Tsukamoto Y, Ichise H, Kakuda H, Yamaguchi M. Intake of fermented soybean (natto) increases circulating vitamin K2 (menaquinone-7) and gamma-carboxylated osteocalcin concentration in normal individuals. J Bone Miner Metab. 2000;18(4):216-22.
112. de Oliveira Silva F, Lemos TC, Sandora D, Monteiro M, Perrone D. Fermentation of soybean meal improves isoflavone metabolism after soy biscuit consumption by adults. J Sci Food Agric. 2020;100(7):2991-8.
113. Jang HH, Noh H, Kim HW, Cho SY, Kim HJ, Lee SH, et al. Metabolic tracking of isoflavones in soybean products and biosamples from healthy adults after fermented soybean consumption. Food Chem. 2020;330:127317.
114. Kim MJ, Lee DH, Ahn J, Jang YJ, Ha TY, Do E, et al. Nutrikinetic study of fermented soybean paste (Cheonggukjang) isoflavones according to the Sasang typology. Nutr Res Pract. 2020;14(2):102-8.
115. Lappi J, Salojärvi J, Kolehmainen M, Mykkänen H, Poutanen K, de Vos WM, et al. Intake of whole-grain and fiber-rich rye bread versus refined wheat bread does not differentiate intestinal microbiota composition in finnish adults with metabolic syndrome. J Nutr. 2013;143(5):648-55.
116. Landberg R, Kamal-Eldin A, Åman P, Christensen J, Overvad K, Tjønneland A, et al. Determinants of plasma alkylresorcinol concentration in Danish post-menopausal women. Eur J Clin Nutr. 2011;65(1):94-101.

117. Söderholm PP, Koskela AH, Lundin JE, Tikkanen MJ, Adlercreutz HC. Plasma pharmacokinetics of alkylresorcinol metabolites: New candidate biomarkers for whole-grain rye and wheat intake. Am J Clin Nutr. 2009;90(5):1167-71.
118. Jansson E, Landberg R, Kamal-Eldin A, Wolk A, Vessby B, Aman P. Presence of alkylresorcinols, potential whole grain biomarkers, in human adipose tissue. Brit J Nutr. 2010;104(5):633-6.
119. Shi L, Brunius C, Lindelöf M, Shameh SA, Wu H, Lee I, et al. Targeted metabolomics reveals differences in the extended postprandial plasma metabolome of healthy subjects after intake of whole-grain rye porridges versus refined wheat bread. Mol Nutr Food Res. 2017;61(7).
120. Harder H, Tetens I, Let MB, Meyer AS. Rye bran bread intake elevates urinary excretion of ferulic acid in humans, but does not affect the susceptibility of LDL to oxidation ex vivo. Eur J Nutr. 2004;43(4):230-6.
121. Pantophlet AJ, Wopereis S, Eelderink C, Vonk RJ, Stroeve JH, Bijlsma S, et al. Metabolic profiling reveals differences in plasma concentrations of arabinose and xylose after consumption of fiber-rich pasta and wheat bread with differential rates of systemic appearance of exogenous glucose in healthy men. J Nutr. 2017;147(2):152-60.
122. Bresciani L, Scazzina F, Leonardi R, Dall'Aglio E, Newell M, Dall'Asta M, et al. Bioavailability and metabolism of phenolic compounds from wholegrain wheat and aleurone-rich wheat bread. Mol Nutr Food Res. 2016;60(11):2343-54.
123. Linko-Parvinen AM, Landberg R, Tikkanen MJ, Adlercreutz H, Peñalvo JL. Alkylresorcinols from whole-grain wheat and rye are transported in human plasma lipoproteins. J Nutr. 2007;137(5):1137-42.
124. Linko AM, Adlercreutz H. Whole-grain rye and wheat alkylresorcinols are incorporated into human erythrocyte membranes. Brit J Nutr. 2005;93(1):11-3.
125. Söderholm PP, Lundin JE, Koskela AH, Tikkanen MJ, Adlercreutz HC. Pharmacokinetics of alkylresorcinol metabolites in human urine. Brit J Nutr. 2011;106(7):1040-4.
126. Meija L, Krams I, Cauce V, Samaletdin A, Söderholm P, Meija R, et al. Alkylresorcinol metabolites in urine and plasma as potential biomarkers of rye and wheat fiber consumption in prostate cancer patients and controls. Nutr Cancer. 2015;67(2):258-65.
127. Kyrø C, Kristensen M, Jakobsen MU, Halkjær J, Landberg R, Bueno-De-Mesquita HB, et al. Dietary intake of whole grains and plasma alkylresorcinol concentrations in relation to changes in anthropometry: The Danish diet, cancer and health cohort study. Eur J Clin Nutr. 2017;71(8):944-52.
128. Vitaglione P, Mennella I, Ferracane R, Rivellese AA, Giacco R, Ercolini D, et al. Whole-grain wheat consumption reduces inflammation in a randomized controlled trial on overweight and obese subjects with unhealthy dietary and lifestyle behaviors: role of polyphenols bound to cereal dietary fiber. Am J Clin Nutr. 2015;101(2):251-61.
129. Zhu Y, Shurlknight KL, Chen X, Sang S. Identification and pharmacokinetics of novel alkylresorcinol metabolites in human urine, new candidate biomarkers for whole-grain wheat and rye intake. J Nutr. 2014;144(2):114-22.
130. Linko AM, Juntunen KS, Mykkänen HM, Adlercreutz H. Whole-grain rye bread consumption by women correlates with plasma alkylresorcinols and increases their concentration compared with low-fiber wheat bread. J Nutr. 2005;135(3):580-3.
131. Ross AB, Kamal-Eldin A, Lundin EA, Zhang JX, Hallmans G, Aman P. Cereal alkylresorcinols are absorbed by humans. J Nutr. 2003;133(7):2222-4.
132. Rizzello CG, Portincasa P, Montemurro M, Di Palo DM, Lorusso MP, De Angelis M, et al. Sourdough Fermented Breads are More Digestible than Those Started with Baker's Yeast Alone: An In Vivo Challenge Dissecting Distinct Gastrointestinal Responses. Nutrients. 2019;11(12).
133. Sandberg JC, Bjorck IME, Nilsson AC. Impact of rye-based evening meals on cognitive functions, mood and cardiometabolic risk factors: a randomized controlled study in healthy middle-aged subjects. Nutr J. 2018;17(1):102.
134. Schramm DD, Karim M, Schrader HR, Holt RR, Kirkpatrick NJ, Polagruto JA, et al. Food effects on the absorption and pharmacokinetics of cocoa flavanols. Life Sci. 2003;73(7):857-69.
135. Rein D, Lotito S, Holt RR, Keen CL, Schmitz HH, Fraga CG. Epicatechin in human plasma: in vivo determination and effect of chocolate consumption on plasma oxidation status. J Nutr. 2000;130(8S Suppl):2109S-14S.
136. Actis-Goretta L, Lévèques A, Giuffrida F, Romanov-Michailidis F, Viton F, Barron D, et al. Elucidation of (-)-epicatechin metabolites after ingestion of chocolate by healthy humans. Free Radic Biol Med. 2012;53(4):787-95.
137. Urpi-Sarda M, Monagas M, Khan N, Llorach R, Lamuela-Raventos RM, Jauregui O, et al. Targeted metabolic profiling of phenolics in urine and plasma after regular consumption of cocoa by liquid chromatography-tandem mass spectrometry. J Chromatogr A. 2009;1216(43):7258-67.
138. Roura E, Andres-Lacueva C, Estruch R, Bilbao MLM, Izquierdo-Pulido M, Lamuela-Raventos RM. The effects of milk as a food matrix for polyphenols on the excretion profile of cocoa (-)-epicatechin metabolites in healthy human subjects. Brit J Nutr. 2008;100(4):846-51.

139. Neilson AP, George JC, Janle EM, Mattes RD, Rudolph R, Matusheski NV, et al. Influence of chocolate matrix composition on cocoa flavan-3-ol bioaccessibility in vitro and bioavailability in humans. J Agric Food Chem. 2009;57(20):9418-26.
140. von Kanel R, Meister RE, Stutz M, Kummer P, Arpagaus A, Huber S, et al. Effects of dark chocolate consumption on the prothrombotic response to acute psychosocial stress in healthy men. Thromb Haemost. 2014;112(6):1151-8.
141. Rodriguez-Mateos A, Oruna-Concha MJ, Kwik-Uribe C, Vidal A, Spencer JPE. Influence of sugar type on the bioavailability of cocoa flavanols. Br J Nutr. 2012;108(12):2243-50.
142. Roura E, Andres-Lacueva C, Estruch R, Lamuela-Raventos RM. Total polyphenol intake estimated by a modified Folin-Ciocalteu assay of urine. Clin Chem. 2006;52(4):749-52.
143. Urpi-Sarda M, Monagas M, Khan N, Lamuela-Raventos RM, Santos-Buelga C, Sacanella E, et al. Epicatechin, procyanidins, and phenolic microbial metabolites after cocoa intake in humans and rats. Anal Bioanal Chem. 2009;394(6):1545-56.
144. Spadafranca A, Martinez Conesa C, Sirini S, Testolin G. Effect of dark chocolate on plasma epicatechin levels, DNA resistance to oxidative stress and total antioxidant activity in healthy subjects. Br J Nutr. 2010;103(7):1008-14.
145. Mullen W, Borges G, Donovan JL, Edwards CA, Serafini M, Lean MEJ, et al. Milk decreases urinary excretion but not plasma pharmacokinetics of cocoa flavan-3-ol metabolites in humans. Am J Clin Nutr. 2009;89(6):1784-91.
146. Resman BH, Blumenthal P, Jusko WJ. Breast milk distribution of theobromine from chocolate. J Pediatr. 1977;91(3):477-80.
147. Taubert D, Roesen R, Lehmann C, Jung N, Schomig E. Effects of low habitual cocoa intake on blood pressure and bioactive nitric oxide - A randomized controlled trial. JAMA-J Am Med Assoc. 2007;298(1):49-60.
148. Martínez-López S, Sarriá B, Gómez-Juaristi M, Goya L, Mateos R, Bravo-Clemente L. Theobromine, caffeine, and theophylline metabolites in human plasma and urine after consumption of soluble cocoa products with different methylxanthine contents. Food Res Int. 2014;63:446-55.
149. Khan N, Monagas M, Andres-Lacueva C, Casas R, Urpi-Sarda M, Lamuela-Raventos RM, et al. Regular consumption of cocoa powder with milk increases HDL cholesterol and reduces oxidized LDL levels in subjects at high-risk of cardiovascular disease. Nutr Metab Cardiovasc Dis. 2012;22(12):1046-53.
150. Carnevale R, Loffredo L, Pignatelli P, Nocella C, Bartimoccia S, Di Santo S, et al. Dark chocolate inhibits platelet isoprostanes via NOX2 down-regulation in smokers. J Thromb Haemost. 2012;10(1):125-32.
151. Holt RR, Lazarus SA, Sullards MC, Zhu QY, Schramm DD, Hammerstone JF, et al. Procyanidin dimer B2 [epicatechin-(4beta-8)-epicatechin] in human plasma after the consumption of a flavanol-rich cocoa. Am J Clin Nutr. 2002;76(4):798-804.
152. Saha S, Hollands W, Needs PW, Ostertag LM, de Roos B, Duthie GG, et al. Human O-sulfated metabolites of (-)-epicatechin and methyl-(-)-epicatechin are poor substrates for commercial aryl-sulfatases: implications for studies concerned with quantifying epicatechin bioavailability. Pharmacol Res. 2012;65(6):592-602.
153. Roura E, Andres-Lacueva C, Estruch R, Mata-Bilbao ML, Izquierdo-Pulido M, Waterhouse AL, et al. Milk does not affect the bioavailability of cocoa powder flavonoid in healthy human. Ann Nutr Metab. 2007;51(6):493-8.
154. Rodriguez-Mateos A, Cifuentes-Gomez T, Gonzalez-Salvador I, Ottaviani JI, Schroeter H, Kelm M, et al. Influence of age on the absorption, metabolism, and excretion of cocoa flavanols in healthy subjects. Mol Nutr Food Res. 2015;59(8):1504-12.
155. Schroeter H, Heiss C, Balzer J, Kleinbongard P, Keen CL, Hollenberg NK, et al. (-)-Epicatechin mediates beneficial effects of flavanol-rich cocoa on vascular function in humans. Proc Natl Acad Sci U S A. 2006;103(4):1024-9.
156. Actis-Goretta L, Leveques A, Giuffrida F, Destaillats F, Nagy K. Identification of O-methyl-(-)-epicatechin-O-sulphate metabolites by mass-spectrometry after O-methylation with trimethylsilyldiazomethane. J Chromatogr A. 2012;1245:150-7.
157. Mogollon JA, Bujold E, Lemieux S, Bourdages M, Blanchet C, Bazinet L, et al. Blood pressure and endothelial function in healthy, pregnant women after acute and daily consumption of flavanol-rich chocolate: a pilot, randomized controlled trial. Nutr J. 2013;12:41.
158. Roura E, Andres-Lacueva C, Jauregui O, Badia E, Estruch R, Izquierdo-Pulido M, et al. Rapid liquid chromatography tandem mass spectrometry assay to quantify plasma (-)-epicatechin metabolites after ingestion of a standard portion of cocoa beverage in humans. J Agric Food Chem. 2005;53(16):6190-4.
159. Stark T, Lang R, Keller D, Hensel A, Hofmann T. Absorption of N-phenylpropenoyl-L-amino acids in healthy humans by oral administration of cocoa (Theobroma cacao). Mol Nutr Food Res. 2008;52(10):1201-14.

160. Rios LY, Gonthier M-P, Remesy C, Mila I, Lapierre C, Lazarus SA, et al. Chocolate intake increases urinary excretion of polyphenol-derived phenolic acids in healthy human subjects. Am J Clin Nutr. 2003;77(4):912-8.
161. Urpi-Sarda M, Llorach R, Khan N, Monagas M, Rotches-Ribalta M, Lamuela-Raventos R, et al. Effect of milk on the urinary excretion of microbial phenolic acids after cocoa powder consumption in humans. J Agric Food Chem. 2010;58(8):4706-11.
162. Roura E, Almajano MP, Bilbao MLM, Andres-Lacueva C, Estruch R, Lamuela-Raventos RM. Human urine: epicatechin metabolites and antioxidant activity after cocoa beverage intake. Free Radic Res. 2007;41(8):943-9.
163. Wang JF, Schramm DD, Holt RR, Ensunsa JL, Fraga CG, Schmitz HH, et al. A dose-response effect from chocolate consumption on plasma epicatechin and oxidative damage. J Nutr. 2000;130(8S Suppl):2115S-9S.
164. Baba S, Osakabe N, Yasuda A, Natsume M, Takizawa T, Nakamura T, et al. Bioavailability of (-)-epicatechin upon intake of chocolate and cocoa in human volunteers. Free Radic Res. 2000;33(5):635-41.
165. Balzer JC, Rassaf T, Heiss C, Lauer T, Merx MW, Heussen N, et al. Sustained benefits in vascular function through flavanol-containing cocoa in medicated diabetic patients: a double-masked, randomized, controlled trial. Eur Heart J. 2008;29:225-6.
166. Engler MB, Engler MM, Chen CY, Malloy MJ, Browne A, Chiu EY, et al. Flavonoid-rich dark chocolate improves endothelial function and increases plasma epicatechin concentrations in healthy adults. J Am Coll Nutr. 2004;23(3):197-204.
167. Loffredo L, Perri L, Catasca E, Pignatelli P, Brancorsini M, Nocella C, et al. Dark chocolate acutely improves walking autonomy in patients with peripheral artery disease. J Am Heart Assoc. 2014;3(4).
168. Mumford GK, Benowitz NL, Evans SM, Kaminski BJ, Preston KL, Sannerud CA, et al. Absorption rate of methylxanthines following capsules, cola and chocolate. Eur J Clin Pharmacol. 1996;51(3-4):319-25.
169. Osakabe N, Baba S, Yasuda A, Iwamoto T, Kamiyama M, Takizawa T, et al. Daily cocoa intake reduces the susceptibility of low-density lipoprotein to oxidation as demonstrated in healthy human volunteers. Free Radic Res. 2001;34(1):93-9.
170. Richelle M, Tavazzi I, Enslen M, Offord EA. Plasma kinetics in man of epicatechin from black chocolate. Eur J Clin Nutr. 1999;53(1):22-6.
171. de O G Mendonca C, Martini LA, Baxmann AC, Nishiura JL, Cuppari L, Sigulem DM, et al. Effects of an oxalate load on urinary oxalate excretion in calcium stone formers. J Ren Nutr. 2003;13(1):39-46.
172. Pruijm M, Hofmann L, Charollais-Thoenig J, Forni V, Maillard M, Coristine A, et al. Effect of dark chocolate on renal tissue oxygenation as measured by BOLD-MRI in healthy volunteers. Clin Nephrol. 2013;80(3):211-7.
173. Shively CA, Tarka SM, Jr., Arnaud MJ, Dvorchik BH, Passananti GT, Vesell ES. High levels of methylxanthines in chocolate do not alter theobromine disposition. Clin Pharmacol Ther. 1985;37(4):415-24.
174. Costa-Bauza A, Grases F, Calvo P, Rodriguez A, Prieto RM. Effect of Consumption of Cocoa-Derived Products on Uric Acid Crystallization in Urine of Healthy Volunteers. Nutrients. 2018;10(10).
175. Ellinger S, Reusch A, Henckes L, Ritter C, Zimmermann BF, Ellinger J, et al. Low Plasma Appearance of (+)-Catechin and (-)-Catechin Compared with Epicatechin after Consumption of Beverages Prepared from Nonalkalized or Alkalized Cocoa-A Randomized, Double-Blind Trial. Nutrients. 2020;12(1).
176. Gomez-Juaristi M, Sarria B, Martinez-Lopez S, Bravo Clemente L, Mateos R. Flavanol Bioavailability in Two Cocoa Products with Different Phenolic Content. A Comparative Study in Humans. Nutrients. 2019;11(7).
177. Loffredo L, Perri L, Battaglia S, Nocella C, Menichelli D, Cammisotto V, et al. Hazelnut and cocoa spread improves flow-mediated dilatation in smokers. Intern Emerg Med. 2018;13(8):1211-7.
178. Montagnana M, Danese E, Angelino D, Mena P, Rosi A, Benati M, et al. Dark chocolate modulates platelet function with a mechanism mediated by flavan-3-ol metabolites. Medicine (Baltimore). 2018;97(49):e13432.
179. Stellingwerff T, Godin J-P, Chou CJ, Grathwohl D, Ross AB, Cooper KA, et al. The effect of acute dark chocolate consumption on carbohydrate metabolism and performance during rest and exercise. Appl Physiol Nutr Metab. 2014;39(2):173-82.
180. Midttun O, Ulvik A, Nygard O, Ueland PM. Performance of plasma trigonelline as a marker of coffee consumption in an epidemiologic setting. Am J Clin Nutr. 2018;107(6):941-7.

181. Stalmach A, Steiling H, Williamson G, Crozier A. Bioavailability of chlorogenic acids following acute ingestion of coffee by humans with an ileostomy. Arch Biochem Biophys. 2010;501(1):98-105.
182. Fumeaux R, Menozzi-Smarrito C, Stalmach A, Munari C, Kraehenbuehl K, Steiling H, et al. First synthesis, characterization, and evidence for the presence of hydroxycinnamic acid sulfate and glucuronide conjugates in human biological fluids as a result of coffee consumption. Org Biomol Chem. 2010;8(22):5199-211.
183. Grant DM, Tang BK, Kalow W. A simple test for acetylator phenotype using caffeine. Br J Clin Pharmacol. 1984;17(4):459-64.
184. Mills CE, Flury A, Marmet C, Poquet L, Rimoldi SF, Sartori C, et al. Mediation of coffee-induced improvements in human vascular function by chlorogenic acids and its metabolites: Two randomized, controlled, crossover intervention trials. Clin Nutr. 2017;36(6):1520-9.
185. Piters KM, Colombo A, Olson HG, Butman SM. Effect of coffee on exercise-induced angina pectoris due to coronary artery disease in habitual coffee drinkers. Am J Cardiol. 1985;55(4):277-80.
186. Hodgson JM, Chan SY, Puddey IB, Devine A, Wattanapenpaiboon N, Wahlqvist ML, et al. Phenolic acid metabolites as biomarkers for tea- and coffee-derived polyphenol exposure in human subjects. Br J Nutr. 2004;91(2):301-6.
187. Kempf K, Herder C, Erlund I, Kolb H, Martin S, Carstensen M, et al. Effects of coffee consumption on subclinical inflammation and other risk factors for type 2 diabetes: A clinical trial. Am J Clin Nutr. 2010;91(4):950-7.
188. Altmaier E, Kastenmuller G, Romisch-Margl W, Thorand B, Weinberger KM, Adamski J, et al. Variation in the human lipidome associated with coffee consumption as revealed by quantitative targeted metabolomics. Mol Nutr Food Res. 2009;53(11):1357-65.
189. Miranda AM, Carioca AAF, Steluti J, da Silva IDCG, Fisberg RM, Marchioni DM. The effect of coffee intake on lysophosphatidylcholines: A targeted metabolomic approach. Clin Nutr. 2017;36(6):1635-41.
190. Takechi R, Alfonso H, Harrison A, Hiramatsu N, Ishisaka A, Tanaka A, et al. Assessing self-reported green tea and coffee consumption by food frequency questionnaire and food record and their association with polyphenol biomarkers in Japanese women. Asia Pac J Clin Nutr. 2018;27(2):460-5.
191. Lang R, Wahl A, Skurk T, Yagar EF, Schmiech L, Eggers R, et al. Development of a hydrophilic liquid interaction chromatography-high-performance liquid chromatography-tandem mass spectrometry based stable isotope dilution analysis and pharmacokinetic studies on bioactive pyridines in human plasma and urine after coffee consumption. Anal Chem. 2010;82(4):1486-97.
192. Allard E, Backstrom D, Danielsson R, Sjobberg JR, Bergquist J. Comparing Capillary Electrophoresis - Mass Spectrometry Fingerprints of Urine Samples Obtained after Intake of Coffee, Tea, or Water. Anal Chem. 2008;80(23):8946-55.
193. Monteiro M, Farah A, Perrone D, Trugo LC, Donangelo C. Chlorogenic acid compounds from coffee are differentially absorbed and metabolized in humans. J Nutr. 2007;137(10):2196-201.
194. Stalmach A, Williamson G, Crozier A. Impact of dose on the bioavailability of coffee chlorogenic acids in humans. Food Funct. 2014;5(8):1727-37.
195. Renouf M, Marmet C, Giuffrida F, Lepage M, Barron D, Beaumont M, et al. Dose-response plasma appearance of coffee chlorogenic and phenolic acids in adults. Mol Nutr Food Res. 2014;58(2):301-9.
196. Wong P, Bachki A, Banerjee K, Leyland-Jones B. Identification of N1-methyl-2-pyridone-5-carboxamide and N1-methyl-4-pyridone-5-carboxamide as components in urine extracts of individuals consuming coffee. J Pharm Biomed Anal. 2002;30(3):773-80.
197. White JR, Jr., Padowski JM, Zhong Y, Chen G, Luo S, Lazarus P, et al. Pharmacokinetic analysis and comparison of caffeine administered rapidly or slowly in coffee chilled or hot versus chilled energy drink in healthy young adults. Clin Toxicol (Phila). 2016;54(4):308-12.
198. Felberg I, Farah A, Monteiro MC, Godoy RLDO, Pacheco S, Calado V, et al. Effect of simultaneous consumption of soymilk and coffee on the urinary excretion of isoflavones, chlorogenic acids and metabolites in healthy adults. J Funct Foods. 2015;19:688-99.
199. Schneider H, Ma L, Glatt H. Extractionless method for the determination of urinary caffeine metabolites using high-performance liquid chromatography coupled with tandem mass spectrometry. J Chromatogr B Analyt Technol Biomed Life Sci. 2003;789(2):227-37.
200. Farrell TL, Gomez-Juaristi M, Poquet L, Redeuil K, Nagy K, Renouf M, et al. Absorption of dimethoxycinnamic acid derivatives in vitro and pharmacokinetic profile in human plasma following coffee consumption. Mol Nutr Food Res. 2012;56(9):1413-23.
201. Guy PA, Renouf M, Barron D, Cavin C, Dionisi F, Kochhar S, et al. Quantitative analysis of plasma caffeic and ferulic acid equivalents by liquid chromatography tandem mass spectrometry. J Chromatogr B Analyt Technol Biomed Life Sci. 2009;877(31):3965-74.

202. Stalmach A, Mullen W, Barron D, Uchida K, Yokota T, Cavin C, et al. Metabolite profiling of hydroxycinnamate derivatives in plasma and urine after the ingestion of coffee by humans: identification of biomarkers of coffee consumption. Drug Metab Dispos. 2009;37(8):1749-58.
203. Duarte GS, Farah A. Effect of simultaneous consumption of milk and coffee on chlorogenic acids' bioavailability in humans. J Agric Food Chem. 2011;59(14):7925-31.
204. Kremer JI, Gompel K, Bakuradze T, Eisenbrand G, Richling E. Urinary Excretion of Niacin Metabolites in Humans After Coffee Consumption. Mol Nutr Food Res. 2018;62(7):e1700735.
205. Martinez-Lopez S, Sarria B, Baeza G, Mateos R, Bravo-Clemente L. Pharmacokinetics of caffeine and its metabolites in plasma and urine after consuming a soluble green/roasted coffee blend by healthy subjects. Food Res Int. 2014;64:125-33.
206. Marmet C, Actis-Goretta L, Renouf M, Giuffrida F. Quantification of phenolic acids and their methylates, glucuronides, sulfates and lactones metabolites in human plasma by LC-MS/MS after oral ingestion of soluble coffee. J Pharm Biomed Anal. 2014;88:617-25.
207. Renouf M, Guy PA, Marmet C, Fraering A-L, Longet K, Moulin J, et al. Measurement of caffeic and ferulic acid equivalents in plasma after coffee consumption: small intestine and colon are key sites for coffee metabolism. Mol Nutr Food Res. 2010;54(6):760-6.
208. Renouf M, Guy P, Marmet C, Longet K, Fraering A-L, Moulin J, et al. Plasma appearance and correlation between coffee and green tea metabolites in human subjects. Br J Nutr. 2010;104(11):1635-40.
209. Renouf M, Marmet C, Guy P, Fraering A-L, Longet K, Moulin J, et al. Nondairy creamer, but not milk, delays the appearance of coffee phenolic acid equivalents in human plasma. J Nutr. 2010;140(2):259-63.
210. Ogawa M, Suzuki Y, Endo Y, Kawamoto T, Kayama F. Influence of coffee intake on urinary hippuric acid concentration. Ind Health. 2011;49(2):195-202.
211. Gomez-Juaristi M, Martinez-Lopez S, Sarria B, Bravo L, Mateos R. Bioavailability of hydroxycinnamates in an instant green/roasted coffee blend in humans. Identification of novel colonic metabolites. Food Funct. 2018;9(1):331-43.
212. Petrovic D, Estoppey Younes S, Pruijm M, Ponte B, Ackermann D, Ehret G, et al. Relation of 24-hour urinary caffeine and caffeine metabolite excretions with self-reported consumption of coffee and other caffeinated beverages in the general population. Nutr Metab (Lond). 2016;13:81.
213. Wong CC, Meinl W, Glatt H-R, Barron D, Stalmach A, Steiling H, et al. In vitro and in vivo conjugation of dietary hydroxycinnamic acids by UDP-glucuronosyltransferases and sulfotransferases in humans. J Nutr Biochem. 2010;21(11):1060-8.
214. Teekachunhatean S, Tosri N, Rojanasthien N, Srichairatanakool S, Sangdee C. Pharmacokinetics of Caffeine following a Single Administration of Coffee Enema versus Oral Coffee Consumption in Healthy Male Subjects. ISRN Pharmacol. 2013;2013:147238.
215. Erk T, Williamson G, Renouf M, Marmet C, Steiling H, Dionisi F, et al. Dose-dependent absorption of chlorogenic acids in the small intestine assessed by coffee consumption in ileostomists. Mol Nutr Food Res. 2012;56(10):1488-500.
216. Nagy K, Redeuil K, Williamson G, Rezzi S, Dionisi F, Longet K, et al. First identification of dimethoxycinnamic acids in human plasma after coffee intake by liquid chromatography-mass spectrometry. J Chromatogr A. 2011;1218(3):491-7.
217. Wagenstaller M, Buettner A. Coffee aroma constituents and odorant metabolites in human urine. Metabolomics. 2014;10(2):225-40.
218. Bonati M, Latini R, Galletti F, Young JF, Tognoni G, Garattini S. Caffeine disposition after oral doses. Clin Pharmacol Ther. 1982;32(1):98-106.
219. Hiramoto K, Kida T, Kikugawa K. Increased urinary hydrogen peroxide levels caused by coffee drinking. Biol Pharm Bull. 2002;25(11):1467-71.
220. Long LH, Halliwell B. Coffee drinking increases levels of urinary hydrogen peroxide detected in healthy human volunteers. Free Radic Res. 2000;32(5):463-7.
221. Rechner AR, Spencer JP, Kuhnle G, Hahn U, Rice-Evans CA. Novel biomarkers of the metabolism of caffeic acid derivatives in vivo. Free Radic Biol Med. 2001;30(11):1213-22.
222. Sanchez-Bridge B, Renouf M, Sauser J, Beaumont M, Actis-Goretta L. The roasting process does not influence the extent of conjugation of coffee chlorogenic and phenolic acids. Biofactors. 2016;42(3):259-67.
223. Smits P, Thien T, van't Laar A. Circulatory effects of coffee in relation to the pharmacokinetics of caffeine. Am J Cardiol. 1985;56(15):958-63.
224. Kremer JI, Pickard S, Stadlmair LF, Glass-Theis A, Buckel L, Bakuradze T, et al. Alkylpyrazines from Coffee are Extensively Metabolized to Pyrazine Carboxylic Acids in the Human Body. Mol Nutr Food Res. 2019:e1801341.
225. Lang R, Dieminger N, Beusch A, Lee Y-M, Dunkel A, Suess B, et al. Bioappearance and pharmacokinetics of bioactives upon coffee consumption. Anal Bioanal Chem. 2013;405(26):8487-503.
226. Ito H, Gonthier MP, Manach C, Morand C, Mennen L, Remesy C, et al. Polyphenol levels in human urine after intake of six different polyphenol-rich beverages. Br J Nutr. 2005;94(4):500-9.

227. Mennen LI, Sapinho D, Ito H, Bertrais S, Galan P, Hercberg S, et al. Urinary flavonoids and phenolic acids as biomarkers of intake for polyphenol-rich foods. Br J Nutr. 2006;96(1):191-8.
228. Noh H, Freisling H, Assi N, Zamora-Ros R, Achaintre D, Affret A, et al. Identification of Urinary Polyphenol Metabolite Patterns Associated with Polyphenol-Rich Food Intake in Adults from Four European Countries. Nutrients. 2017;9(8).
229. Edmands WM, Ferrari P, Rothwell JA, Rinaldi S, Slimani N, Barupal DK, et al. Polyphenol metabolome in human urine and its association with intake of polyphenol-rich foods across European countries. Am J Clin Nutr. 2015;102(4):905-13.
230. Floegel A, von Ruesten A, Drogan D, Schulze MB, Prehn C, Adamski J, et al. Variation of serum metabolites related to habitual diet: a targeted metabolomic approach in EPIC-Potsdam. Eur J Clin Nutr. 2013;67(10):1100-8.
231. Svilaas A, Sakhi AK, Andersen LF, Svilaas T, Strom EC, Jacobs DR, Jr., et al. Intakes of antioxidants in coffee, wine, and vegetables are correlated with plasma carotenoids in humans. J Nutr. 2004;134(3):562-7.
232. Bresciani L, Tassotti M, Rosi A, Martini D, Antonini M, Dei Cas A, et al. Absorption, Pharmacokinetics, and Urinary Excretion of Pyridines After Consumption of Coffee and Cocoa-Based Products Containing Coffee in a Repeated Dose, Crossover Human Intervention Study. Mol Nutr Food Res. 2020:e2000489.
233. Sugiyama S, Fushimi T, Kishi M, Irie S, Tsuji S, Hosokawa N, et al. Bioavailability of acetate from two vinegar supplements: capsule and drink. J Nutr Sci Vitaminol (Tokyo). 2010;56(4):266-9.
234. Jasbi P, Baker O, Shi X, Gonzalez LA, Wang S, Anderson S, et al. Daily red wine vinegar ingestion for eight weeks improves glucose homeostasis and affects the metabolome but does not reduce adiposity in adults. Food Funct. 2019;10(11):7343-55.
235. Peters A, Krumbholz P, Jager E, Heintz-Buschart A, Cakir MV, Rothemund S, et al. Metabolites of lactic acid bacteria present in fermented foods are highly potent agonists of human hydroxycarboxylic acid receptor 3. PLoS Genet. 2019;15(5):e1008145.
236. Marks SC, Mullen W, Borges G, Crozier A. Absorption, metabolism, and excretion of cider dihydrochalcones in healthy humans and subjects with an ileostomy. J Agric Food Chem. 2009;57(5):2009-15.
237. Hornero-Mendez D, Cerrillo I, Ortega A, Rodriguez-Grinolo M-R, Escudero-Lopez B, Martin F, et al. beta-Cryptoxanthin is more bioavailable in humans from fermented orange juice than from orange juice. Food Chem. 2018;262:215-20.
238. Jin H, Seo J-H, Uhm Y-K, Jung C-Y, Lee S-K, Yim S-V. Pharmacokinetic comparison of ginsenoside metabolite IH-901 from fermented and non-fermented ginseng in healthy Korean volunteers. J Ethnopharmacol. 2012;139(2):664-7.
239. Sawicki T, Topolska J, Romaszko E, Wiczkowski W. Profile and Content of Betalains in Plasma and Urine of Volunteers after Long-Term Exposure to Fermented Red Beet Juice. J Agric Food Chem. 2018;66(16):4155-63.
240. Tümer AR, Lale A, Gürler M, Yıldırım MŞ, Kaynak AD, Akçan R. The effects of traditional fermented beverages on ethanol, ethyl glucuronide and ethyl sulphate levels. Egypt J Forensic Sci. 2018;8(1).
241. Wiczkowski W, Szawara-Nowak D, Romaszko J. The impact of red cabbage fermentation on bioavailability of anthocyanins and antioxidant capacity of human plasma. Food Chem. 2016;190:730-40.
242. Stalmach A, Mullen W, Pecorari M, Serafini M, Crozier A. Bioavailability of C-linked dihydrochalcone and flavanone glucosides in humans following ingestion of unfermented and fermented rooibos teas. J Agric Food Chem. 2009;57(15):7104-11.
243. Xie G, Ye M, Wang Y, Ni Y, Su M, Huang H, et al. Characterization of pu-erh tea using chemical and metabolic profiling approaches. J Agric Food Chem. 2009;57(8):3046-54.
